# Supplementary figures and images for: Stage-specific TRIM10 expression regulates erythroid maturation (part 1 of 3)
Source: EMBO Rep. 2025 Oct 30;26(23):5982–6014. doi: 10.1038/s44319-025-00616-0 (PMC12678476; doi:10.1038/s44319-025-00616-0)

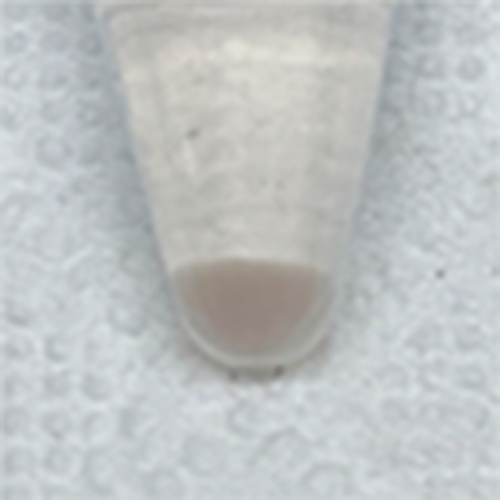

Supplement: Supplementary file 2 — Source data Fig. 1 [file 44319_2025_616_MOESM2_ESM.zip › 1E/Fig1E_pellet_D0.tif]

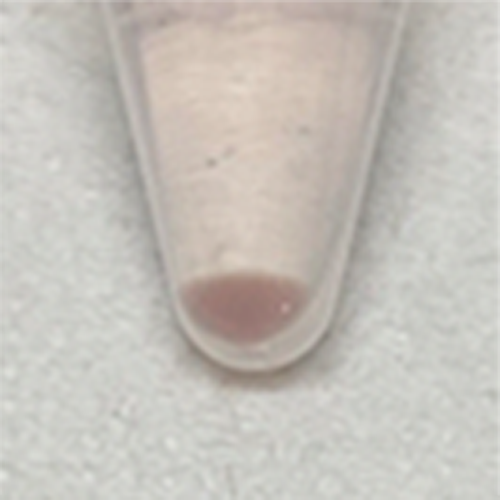

Supplement: Supplementary file 2 — Source data Fig. 1 [file 44319_2025_616_MOESM2_ESM.zip › 1E/Fig1E_pellet_D2.tif]

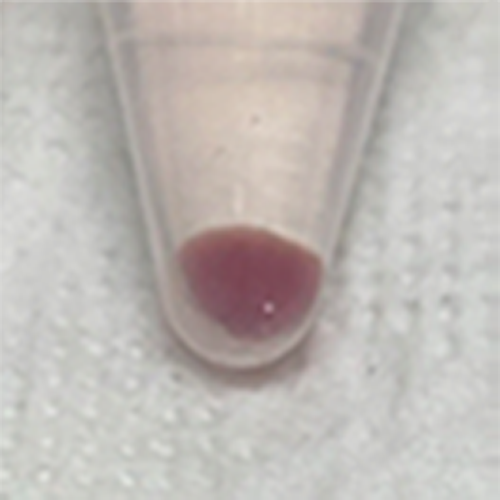

Supplement: Supplementary file 2 — Source data Fig. 1 [file 44319_2025_616_MOESM2_ESM.zip › 1E/Fig1E_pellet_D4.tif]

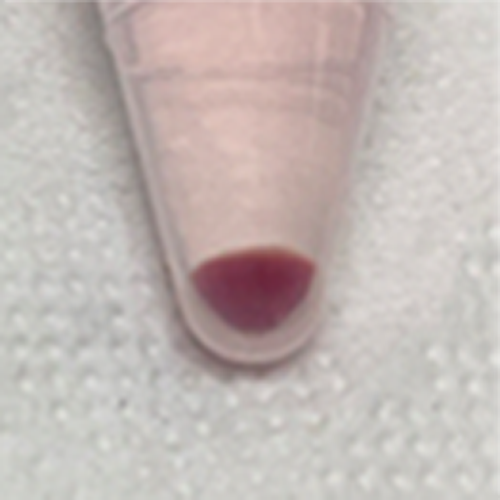

Supplement: Supplementary file 2 — Source data Fig. 1 [file 44319_2025_616_MOESM2_ESM.zip › 1E/Fig1E_pellet_D6.tif]

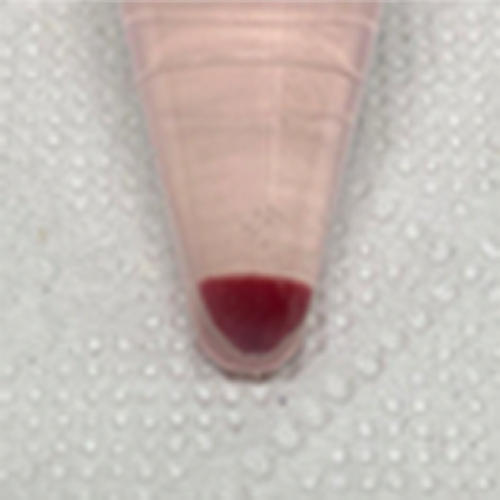

Supplement: Supplementary file 2 — Source data Fig. 1 [file 44319_2025_616_MOESM2_ESM.zip › 1E/Fig1E_pellet_D8.tif]

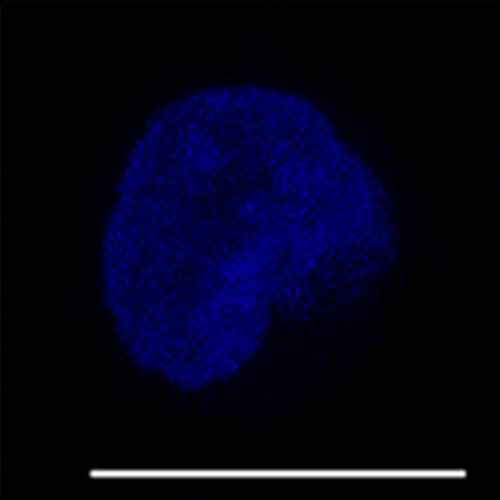

Supplement: Supplementary file 3 — Source data Fig. 2 [file 44319_2025_616_MOESM3_ESM.zip › 2A/Fig2A_D0_DAPI.tif]

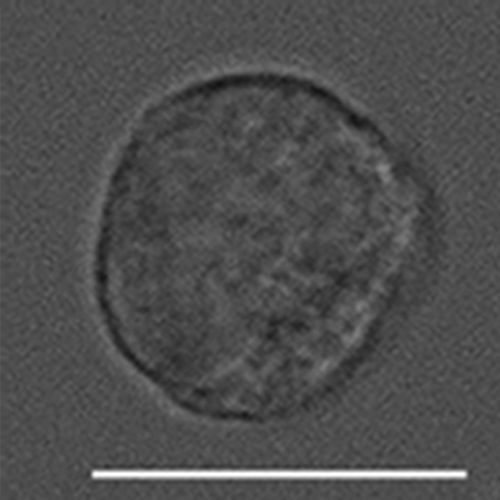

Supplement: Supplementary file 3 — Source data Fig. 2 [file 44319_2025_616_MOESM3_ESM.zip › 2A/Fig2A_D0_DIC.tif]

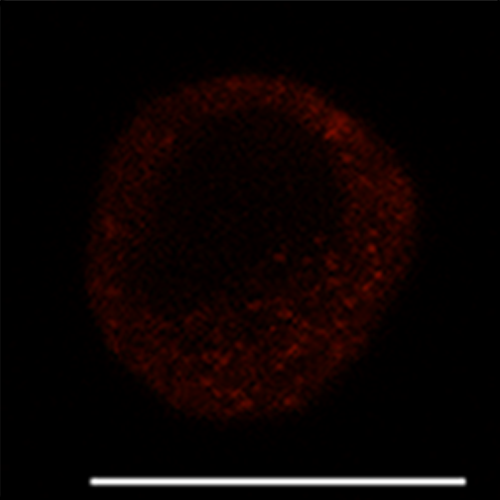

Supplement: Supplementary file 3 — Source data Fig. 2 [file 44319_2025_616_MOESM3_ESM.zip › 2A/Fig2A_D0_HbA.tif]

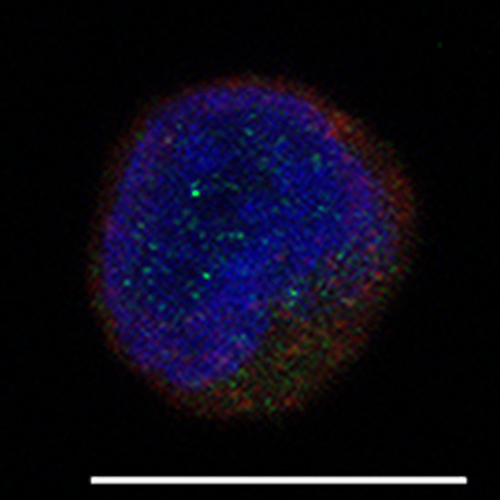

Supplement: Supplementary file 3 — Source data Fig. 2 [file 44319_2025_616_MOESM3_ESM.zip › 2A/Fig2A_D0_Merged.tif]

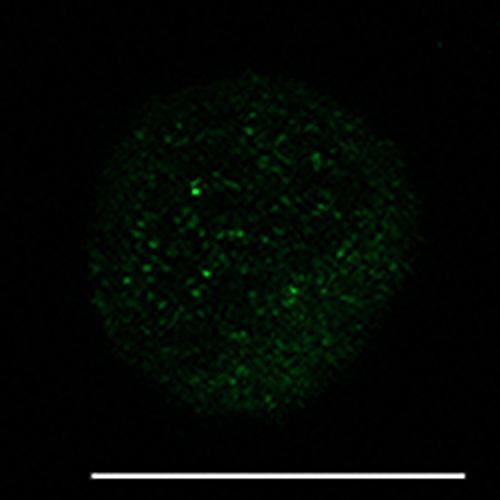

Supplement: Supplementary file 3 — Source data Fig. 2 [file 44319_2025_616_MOESM3_ESM.zip › 2A/Fig2A_D0_TRIM10alpha.tif]

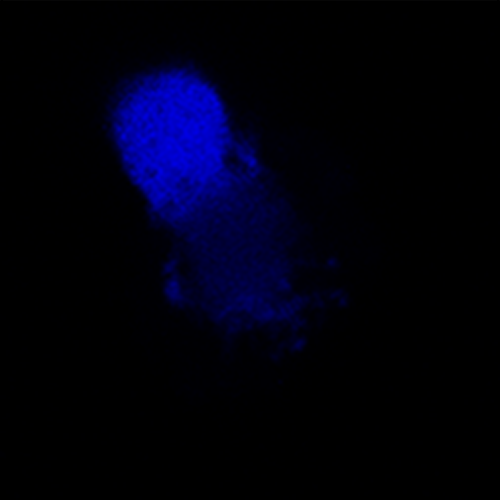

Supplement: Supplementary file 3 — Source data Fig. 2 [file 44319_2025_616_MOESM3_ESM.zip › 2A/Fig2A_D12_Ortho_DAPI.tif]

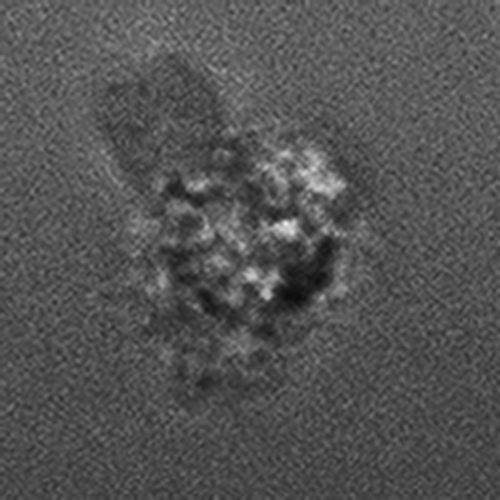

Supplement: Supplementary file 3 — Source data Fig. 2 [file 44319_2025_616_MOESM3_ESM.zip › 2A/Fig2A_D12_Ortho_DIC.tif]

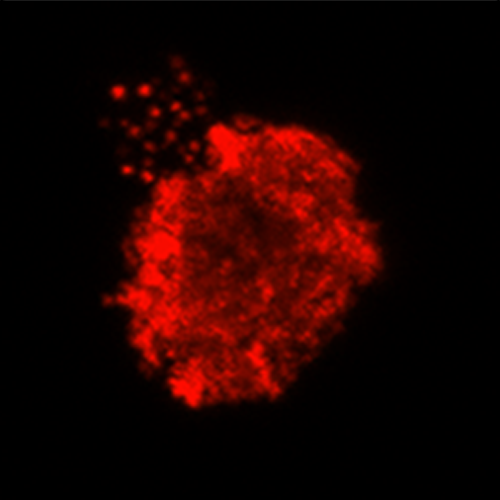

Supplement: Supplementary file 3 — Source data Fig. 2 [file 44319_2025_616_MOESM3_ESM.zip › 2A/Fig2A_D12_Ortho_HbA.tif]

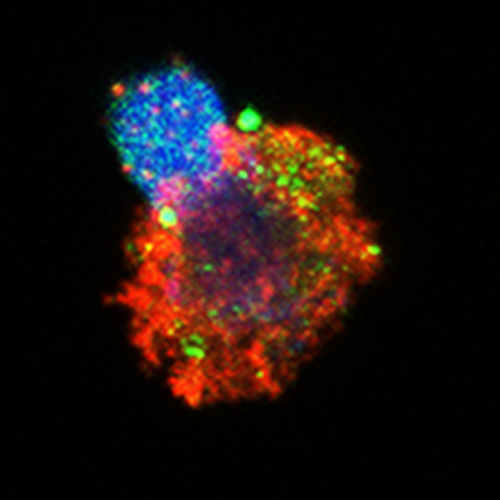

Supplement: Supplementary file 3 — Source data Fig. 2 [file 44319_2025_616_MOESM3_ESM.zip › 2A/Fig2A_D12_Ortho_Merged.tif]

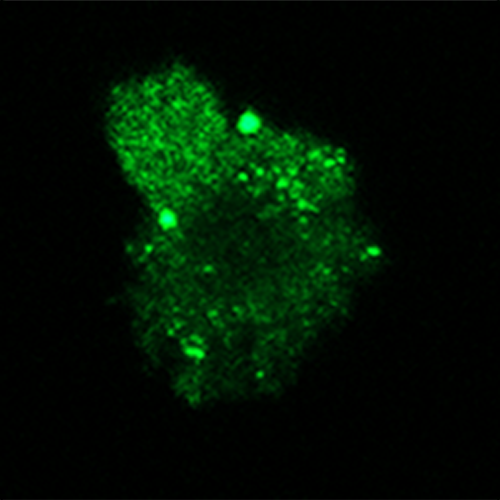

Supplement: Supplementary file 3 — Source data Fig. 2 [file 44319_2025_616_MOESM3_ESM.zip › 2A/Fig2A_D12_Ortho_TRIM10alpha.tif]

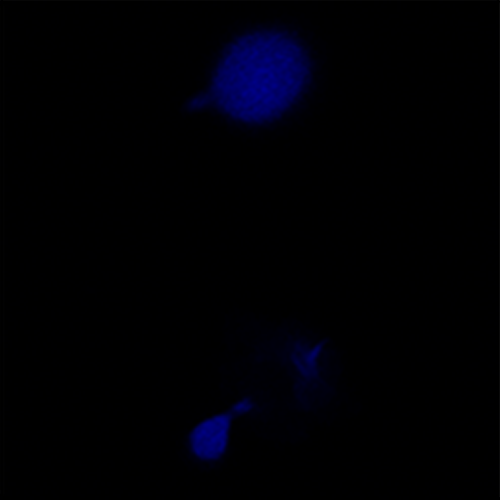

Supplement: Supplementary file 3 — Source data Fig. 2 [file 44319_2025_616_MOESM3_ESM.zip › 2A/Fig2A_D12_Reticulocyte_DAPI.tif]

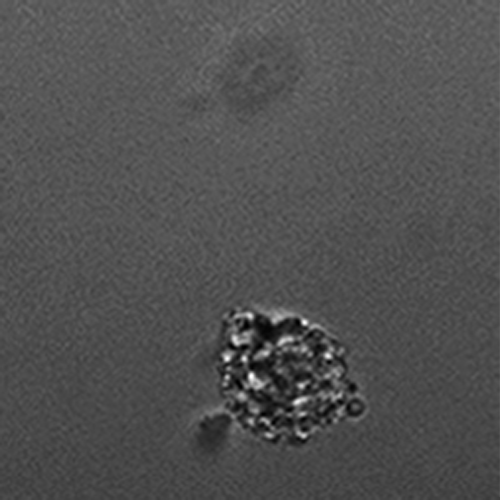

Supplement: Supplementary file 3 — Source data Fig. 2 [file 44319_2025_616_MOESM3_ESM.zip › 2A/Fig2A_D12_Reticulocyte_DIC.tif]

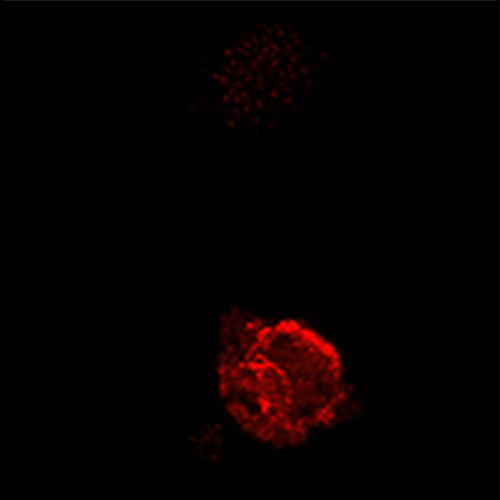

Supplement: Supplementary file 3 — Source data Fig. 2 [file 44319_2025_616_MOESM3_ESM.zip › 2A/Fig2A_D12_Reticulocyte_HbA.tif]

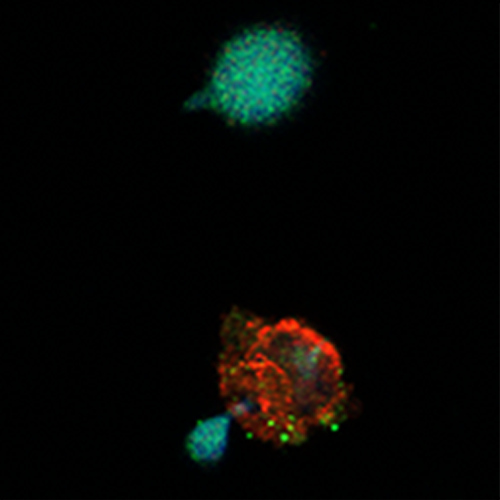

Supplement: Supplementary file 3 — Source data Fig. 2 [file 44319_2025_616_MOESM3_ESM.zip › 2A/Fig2A_D12_Reticulocyte_Merged.tif]

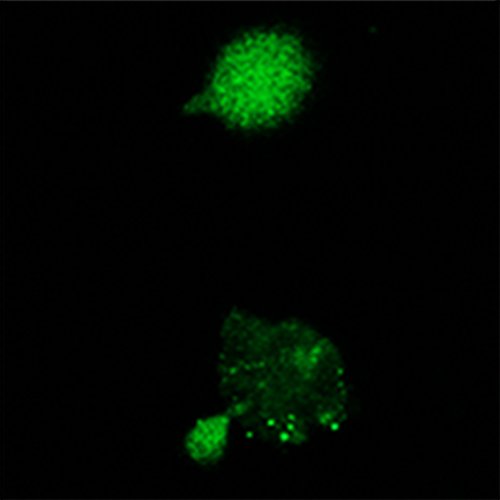

Supplement: Supplementary file 3 — Source data Fig. 2 [file 44319_2025_616_MOESM3_ESM.zip › 2A/Fig2A_D12_Reticulocyte_TRIM10alpha.tif]

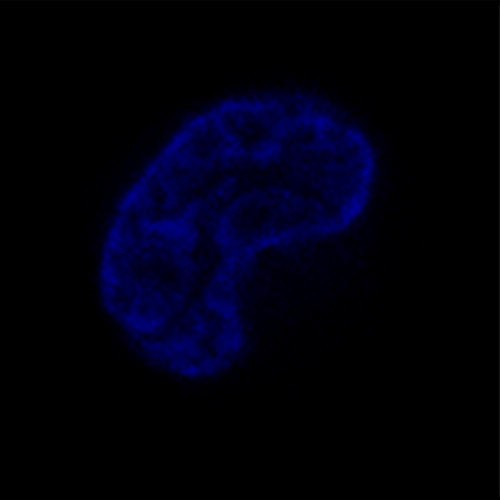

Supplement: Supplementary file 3 — Source data Fig. 2 [file 44319_2025_616_MOESM3_ESM.zip › 2A/Fig2A_D4_DAPI.tif]

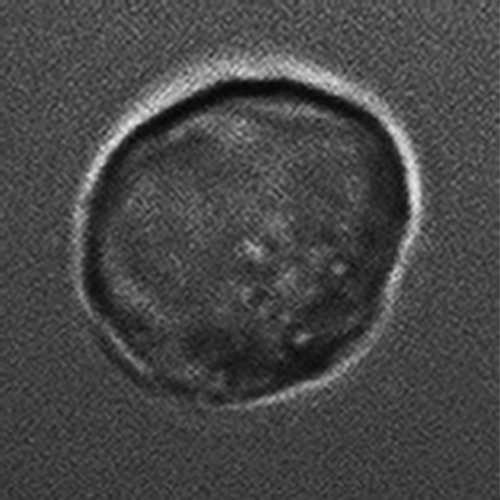

Supplement: Supplementary file 3 — Source data Fig. 2 [file 44319_2025_616_MOESM3_ESM.zip › 2A/Fig2A_D4_DIC.tif]

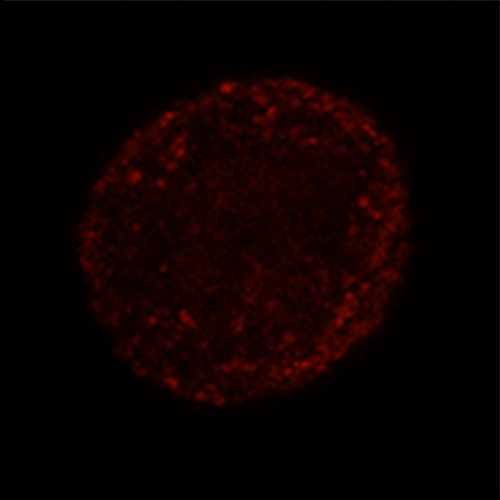

Supplement: Supplementary file 3 — Source data Fig. 2 [file 44319_2025_616_MOESM3_ESM.zip › 2A/Fig2A_D4_HbA.tif]

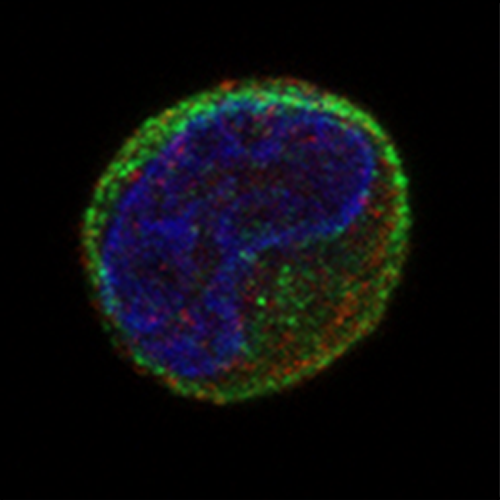

Supplement: Supplementary file 3 — Source data Fig. 2 [file 44319_2025_616_MOESM3_ESM.zip › 2A/Fig2A_D4_Merged.tif]

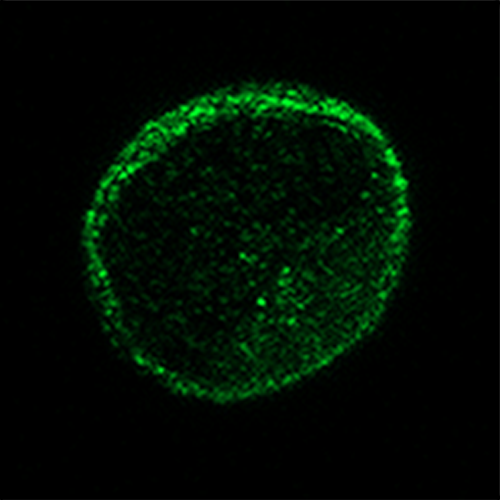

Supplement: Supplementary file 3 — Source data Fig. 2 [file 44319_2025_616_MOESM3_ESM.zip › 2A/Fig2A_D4_TRIM10alpha.tif]

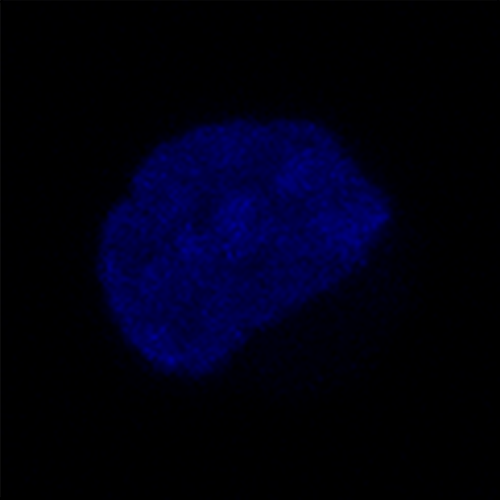

Supplement: Supplementary file 3 — Source data Fig. 2 [file 44319_2025_616_MOESM3_ESM.zip › 2A/Fig2A_D8_DAPI.tif]

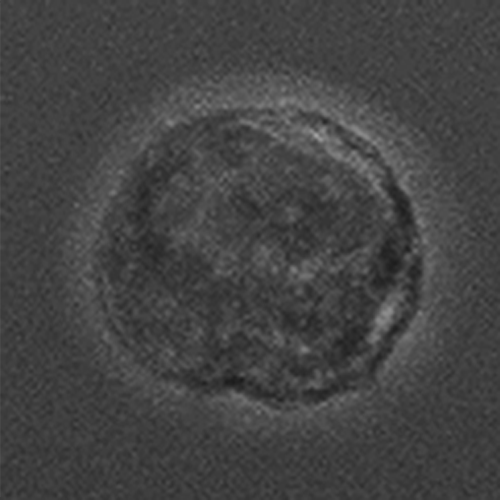

Supplement: Supplementary file 3 — Source data Fig. 2 [file 44319_2025_616_MOESM3_ESM.zip › 2A/Fig2A_D8_DIC.tif]

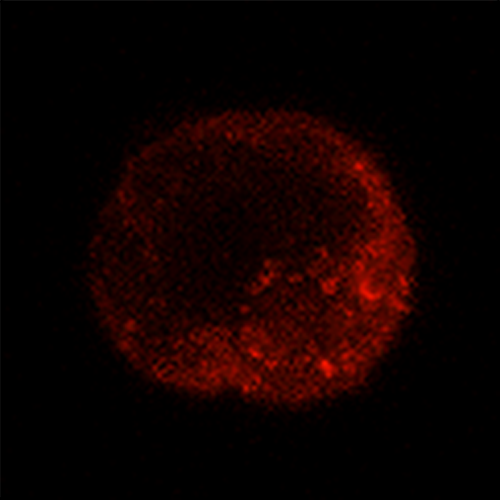

Supplement: Supplementary file 3 — Source data Fig. 2 [file 44319_2025_616_MOESM3_ESM.zip › 2A/Fig2A_D8_HbA.tif]

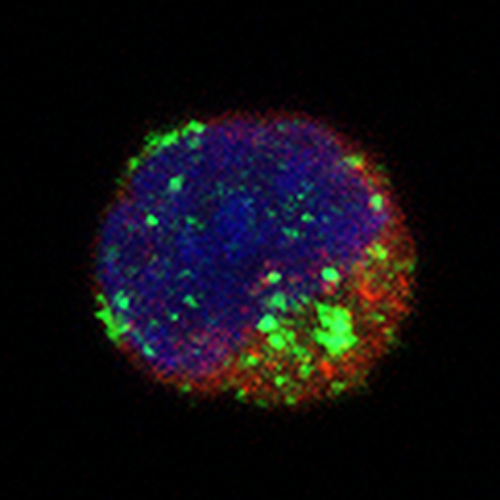

Supplement: Supplementary file 3 — Source data Fig. 2 [file 44319_2025_616_MOESM3_ESM.zip › 2A/Fig2A_D8_Merged.tif]

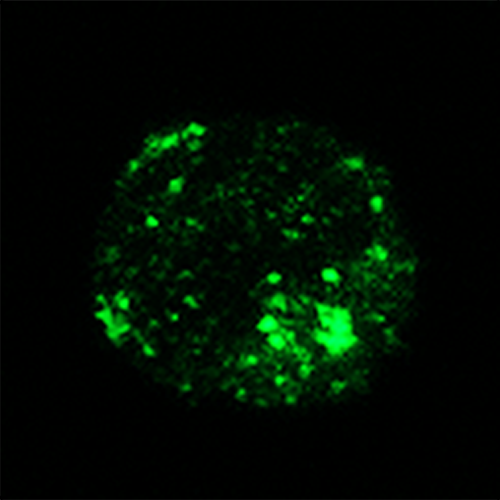

Supplement: Supplementary file 3 — Source data Fig. 2 [file 44319_2025_616_MOESM3_ESM.zip › 2A/Fig2A_D8_TRIM10alpha.tif]

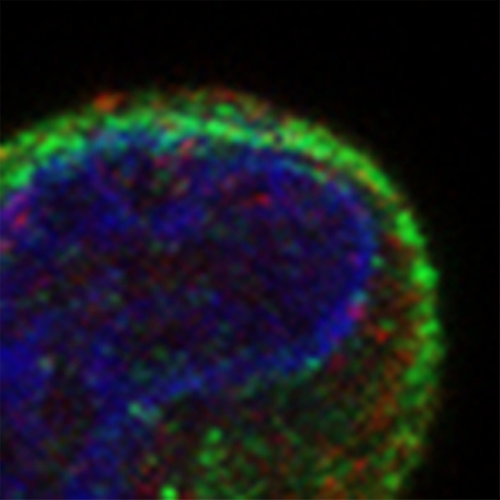

Supplement: Supplementary file 3 — Source data Fig. 2 [file 44319_2025_616_MOESM3_ESM.zip › 2A/Fig2A_Enlarged_image1.tif]

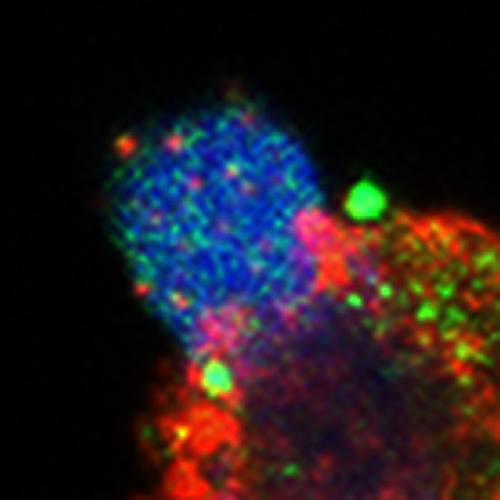

Supplement: Supplementary file 3 — Source data Fig. 2 [file 44319_2025_616_MOESM3_ESM.zip › 2A/Fig2A_Enlarged_image2.tif]

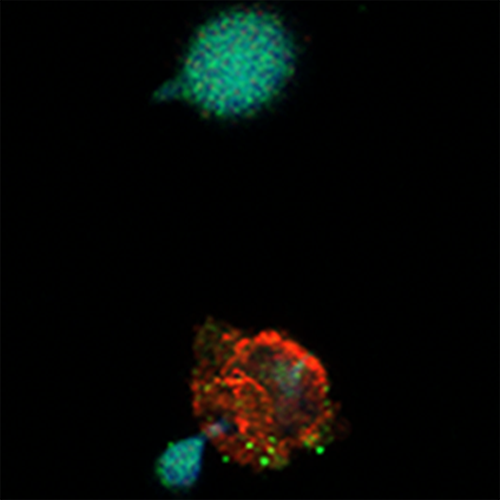

Supplement: Supplementary file 3 — Source data Fig. 2 [file 44319_2025_616_MOESM3_ESM.zip › 2A/Fig2A_Enlarged_image3.tif]

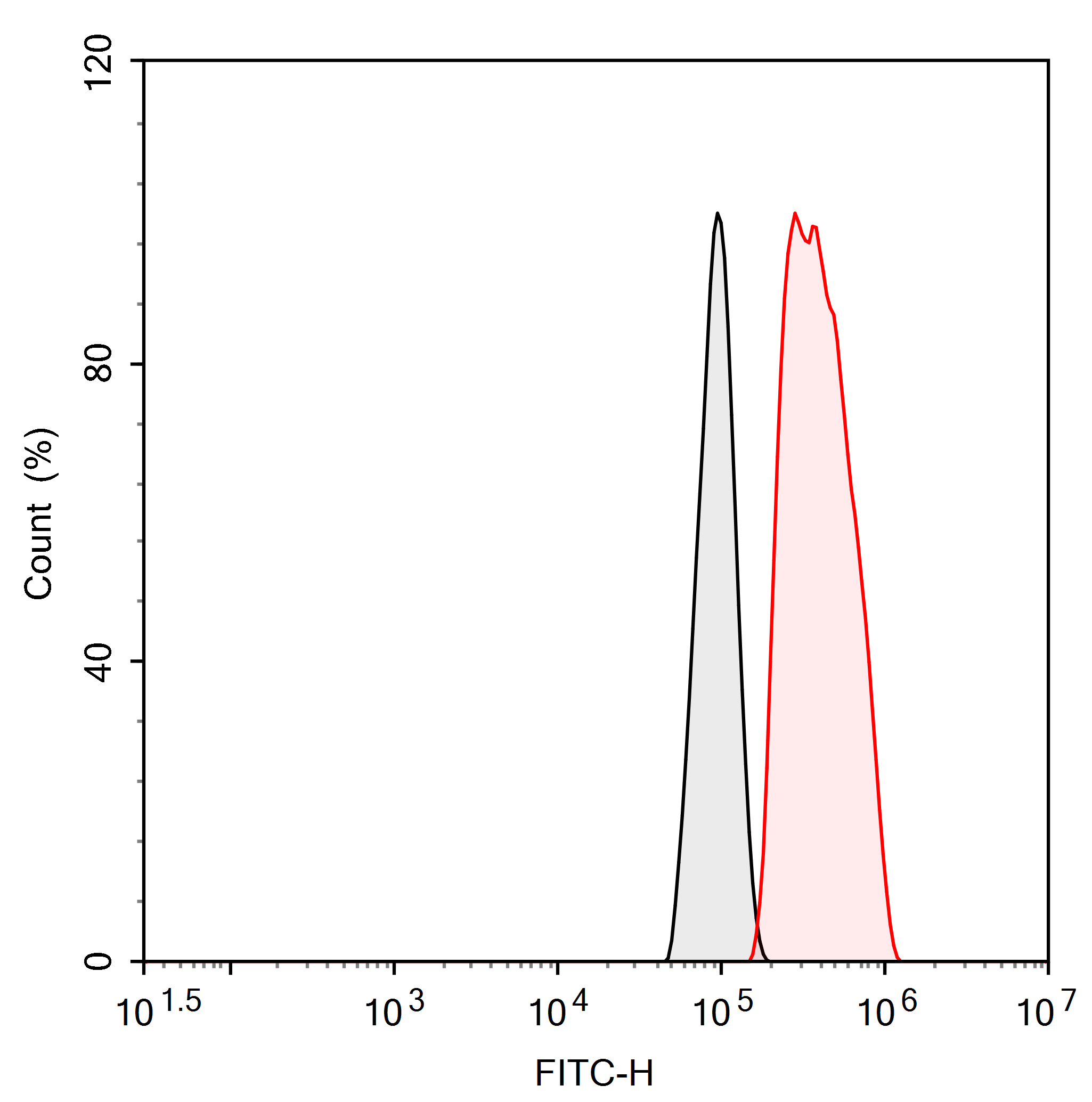

Supplement: Supplementary file 3 — Source data Fig. 2 [file 44319_2025_616_MOESM3_ESM.zip › 2D/Fig2D_Graph_noperm.tiff]

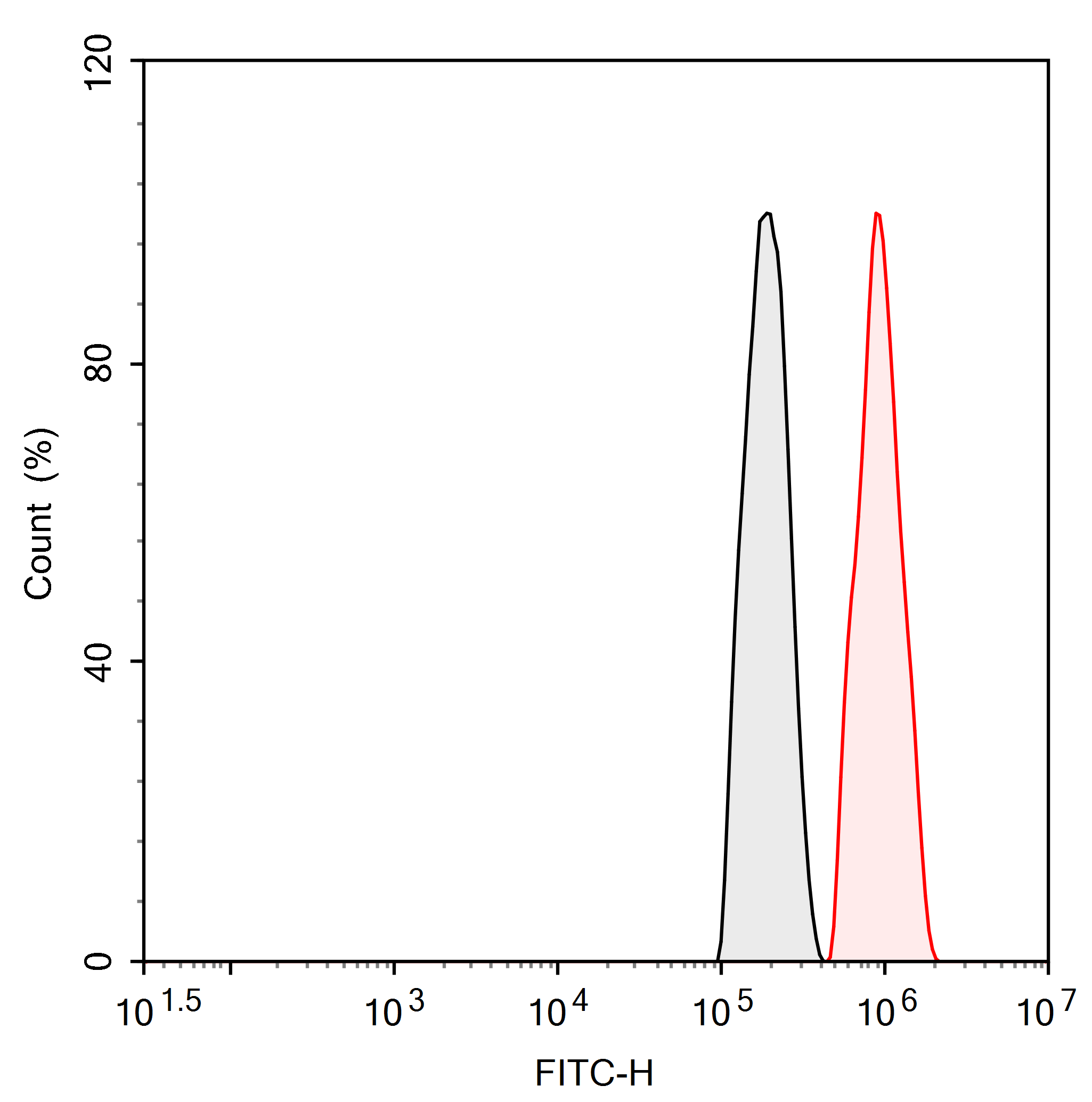

Supplement: Supplementary file 3 — Source data Fig. 2 [file 44319_2025_616_MOESM3_ESM.zip › 2D/Fig2D_Graph_perm.tiff]

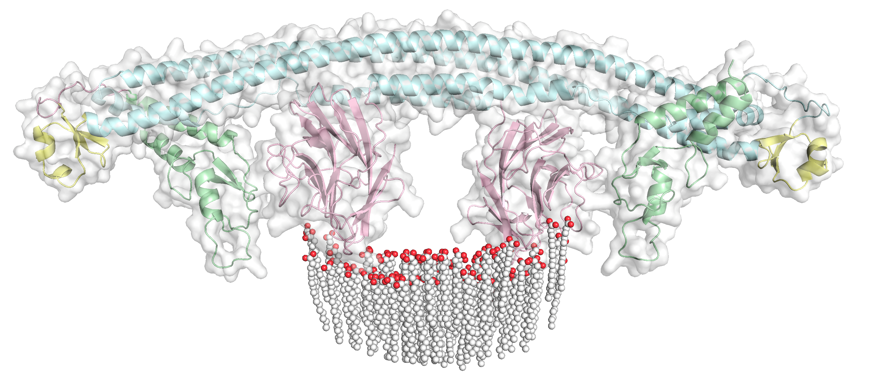

Supplement: Supplementary file 3 — Source data Fig. 2 [file 44319_2025_616_MOESM3_ESM.zip › 2E/Fig2E_TRIM10alpha_Structure.tif]

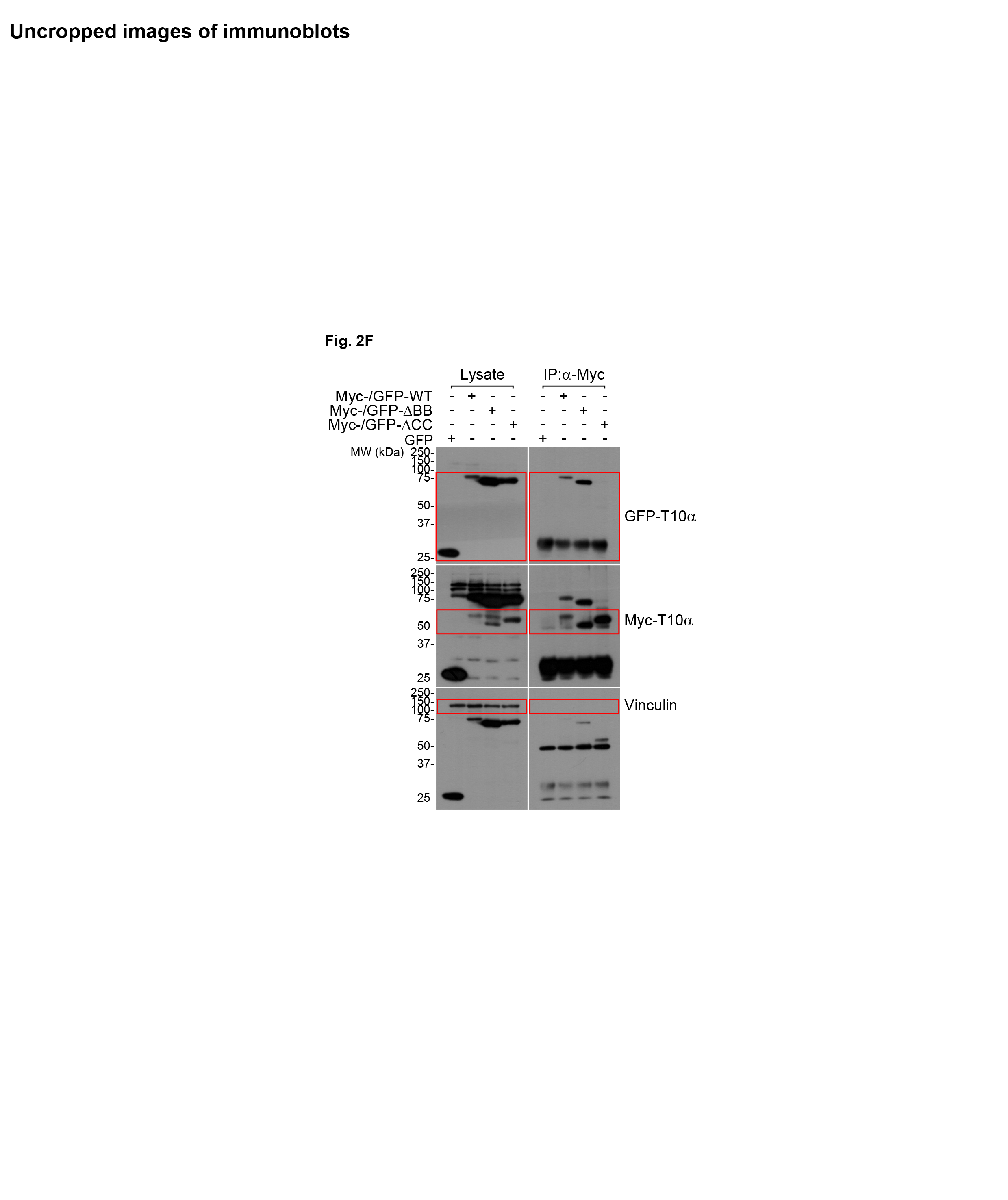

Supplement: Supplementary file 3 — Source data Fig. 2 [file 44319_2025_616_MOESM3_ESM.zip › 2F/Fig2F_Blot_data.tif]

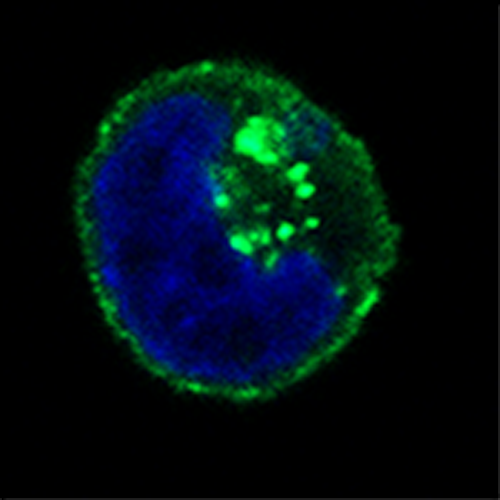

Supplement: Supplementary file 3 — Source data Fig. 2 [file 44319_2025_616_MOESM3_ESM.zip › 2G/Fig2G_dBB_Merged.tif]

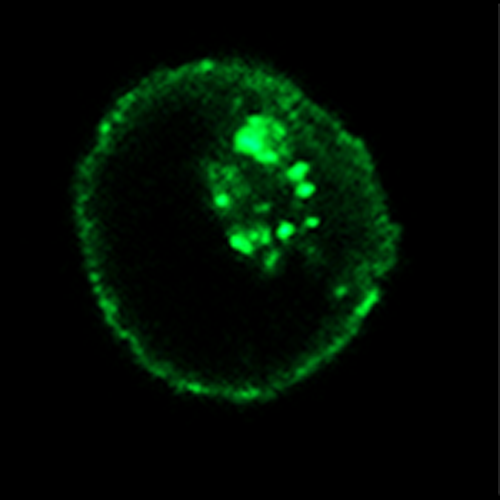

Supplement: Supplementary file 3 — Source data Fig. 2 [file 44319_2025_616_MOESM3_ESM.zip › 2G/Fig2G_dBB_Myc.tif]

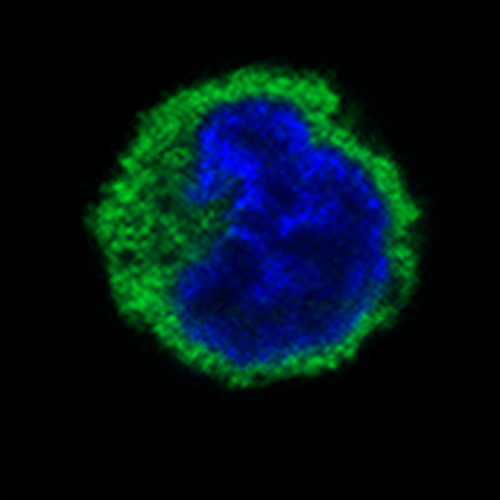

Supplement: Supplementary file 3 — Source data Fig. 2 [file 44319_2025_616_MOESM3_ESM.zip › 2G/Fig2G_dCC_Merged.tif]

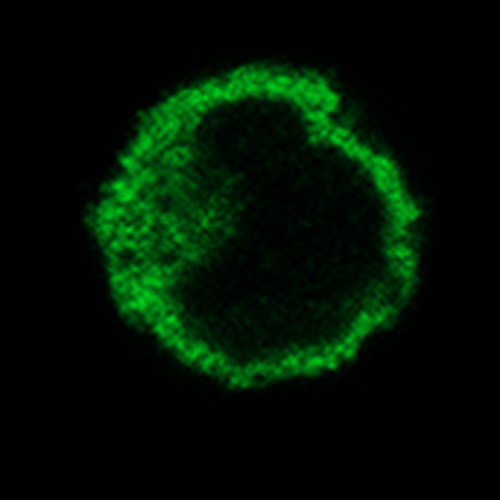

Supplement: Supplementary file 3 — Source data Fig. 2 [file 44319_2025_616_MOESM3_ESM.zip › 2G/Fig2G_dCC_Myc.tif]

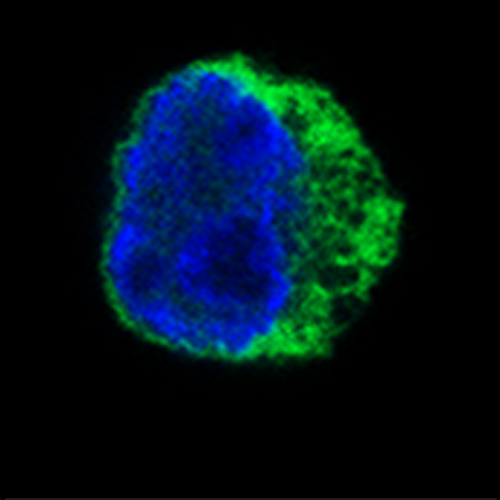

Supplement: Supplementary file 3 — Source data Fig. 2 [file 44319_2025_616_MOESM3_ESM.zip › 2G/Fig2G_dPRYSPRY_Merged.tif]

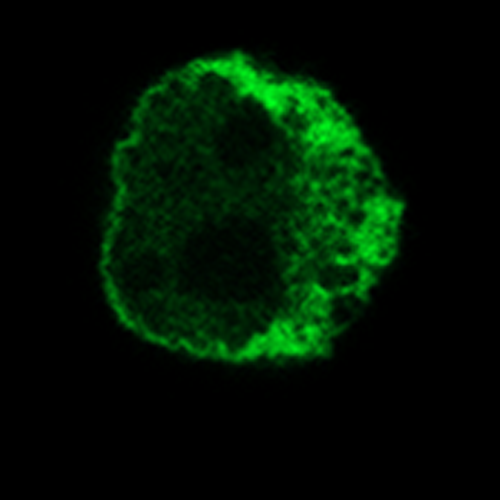

Supplement: Supplementary file 3 — Source data Fig. 2 [file 44319_2025_616_MOESM3_ESM.zip › 2G/Fig2G_dPRYSPRY_Myc.tif]

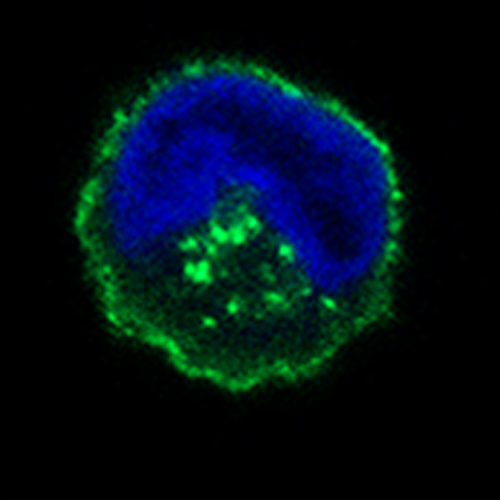

Supplement: Supplementary file 3 — Source data Fig. 2 [file 44319_2025_616_MOESM3_ESM.zip › 2G/Fig2G_dRING_Merged.tif]

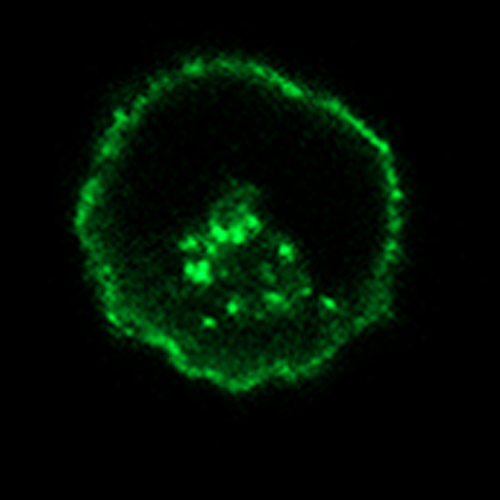

Supplement: Supplementary file 3 — Source data Fig. 2 [file 44319_2025_616_MOESM3_ESM.zip › 2G/Fig2G_dRING_Myc.tif]

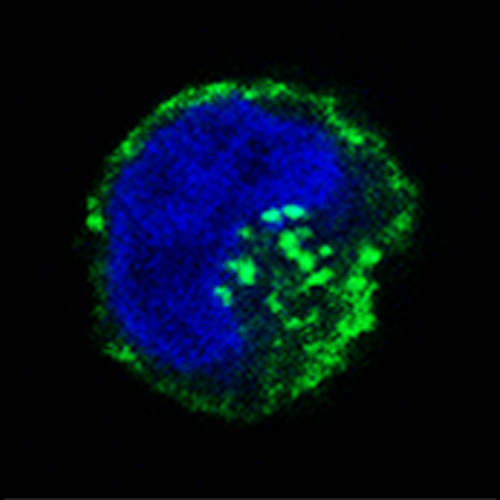

Supplement: Supplementary file 3 — Source data Fig. 2 [file 44319_2025_616_MOESM3_ESM.zip › 2G/Fig2G_WT_Merged.tif]

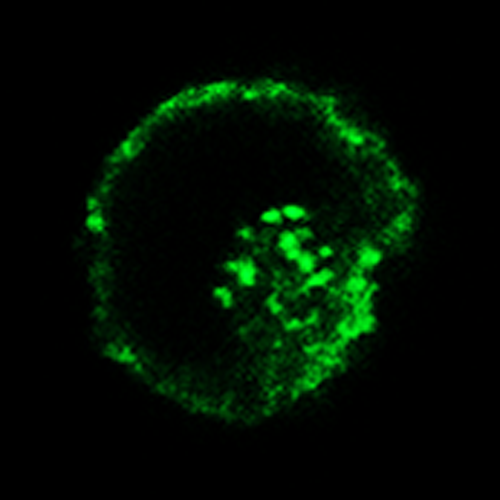

Supplement: Supplementary file 3 — Source data Fig. 2 [file 44319_2025_616_MOESM3_ESM.zip › 2G/Fig2G_WT_Myc.tif]

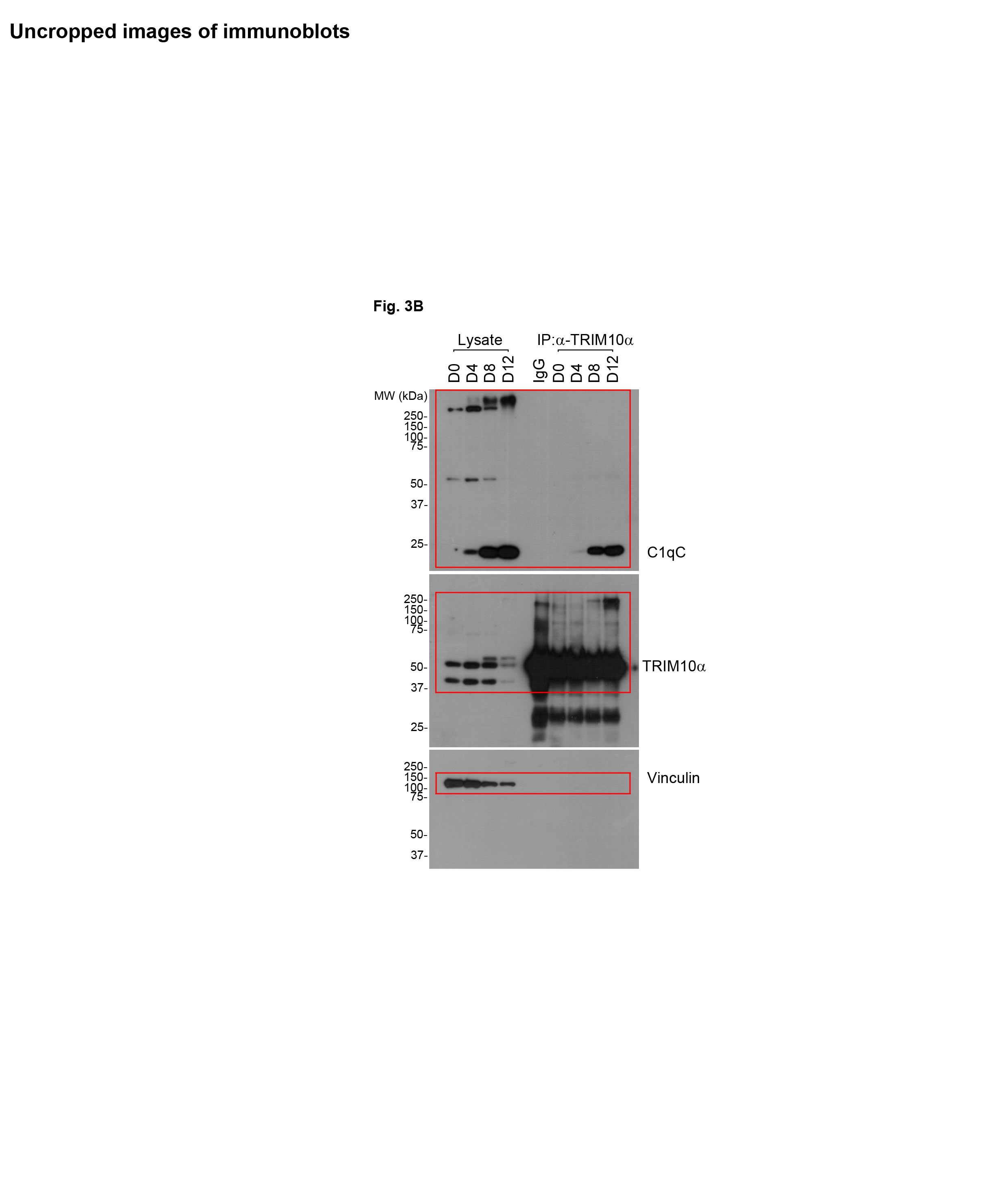

Supplement: Supplementary file 4 — Source data Fig. 3 [file 44319_2025_616_MOESM4_ESM.zip › 3B/Fig3B_Blot_data.tif]

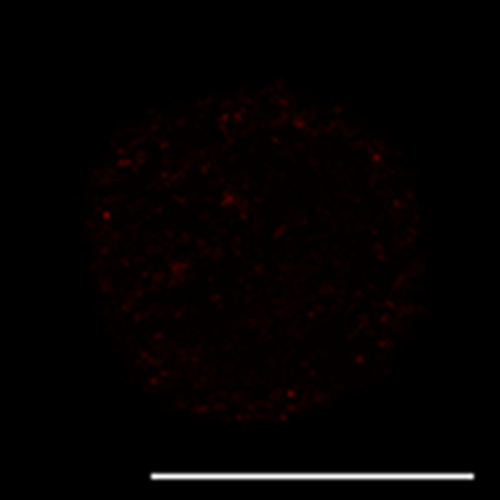

Supplement: Supplementary file 4 — Source data Fig. 3 [file 44319_2025_616_MOESM4_ESM.zip › 3C/Fig3C_D0_C1qC.tif]

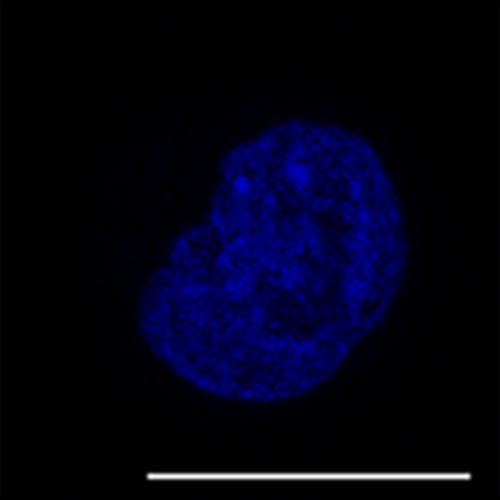

Supplement: Supplementary file 4 — Source data Fig. 3 [file 44319_2025_616_MOESM4_ESM.zip › 3C/Fig3C_D0_DAPI.tif]

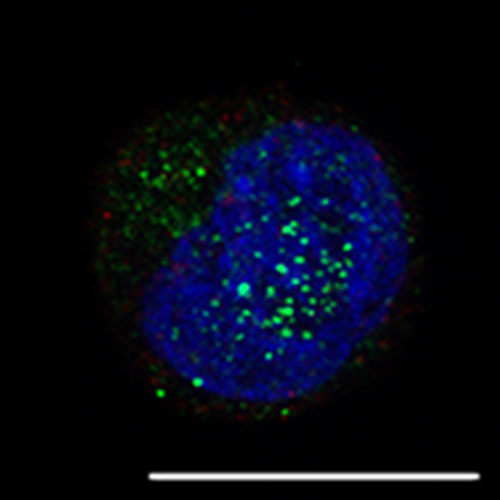

Supplement: Supplementary file 4 — Source data Fig. 3 [file 44319_2025_616_MOESM4_ESM.zip › 3C/Fig3C_D0_Merged.tif]

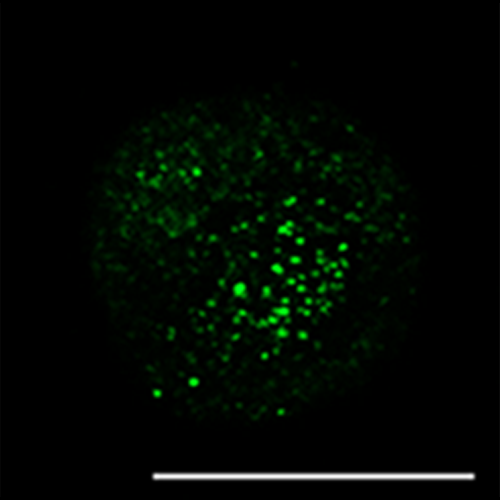

Supplement: Supplementary file 4 — Source data Fig. 3 [file 44319_2025_616_MOESM4_ESM.zip › 3C/Fig3C_D0_TRIM10alpha.tif]

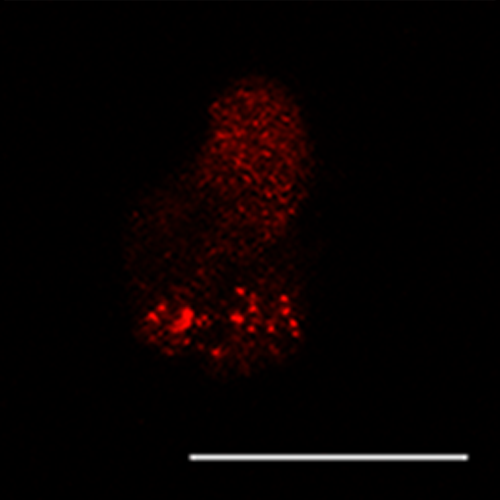

Supplement: Supplementary file 4 — Source data Fig. 3 [file 44319_2025_616_MOESM4_ESM.zip › 3C/Fig3C_D12_C1qC.tif]

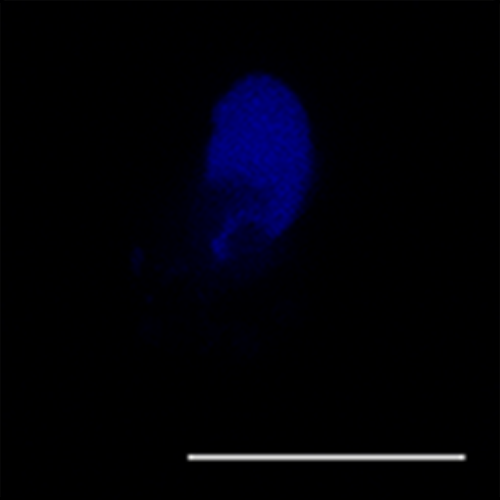

Supplement: Supplementary file 4 — Source data Fig. 3 [file 44319_2025_616_MOESM4_ESM.zip › 3C/Fig3C_D12_DAPI.tif]

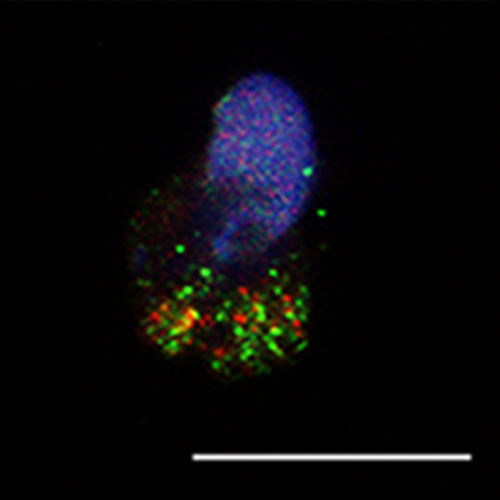

Supplement: Supplementary file 4 — Source data Fig. 3 [file 44319_2025_616_MOESM4_ESM.zip › 3C/Fig3C_D12_Merged.tif]

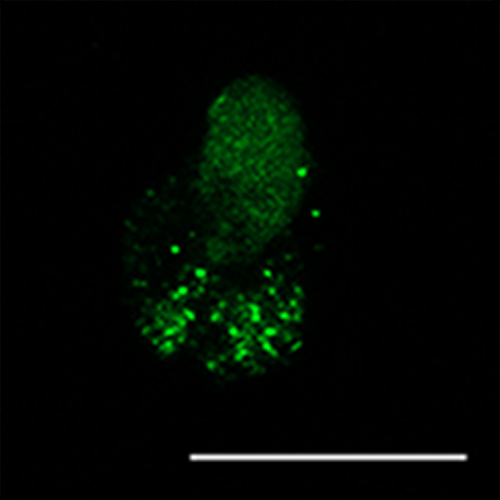

Supplement: Supplementary file 4 — Source data Fig. 3 [file 44319_2025_616_MOESM4_ESM.zip › 3C/Fig3C_D12_TRIM10alpha.tif]

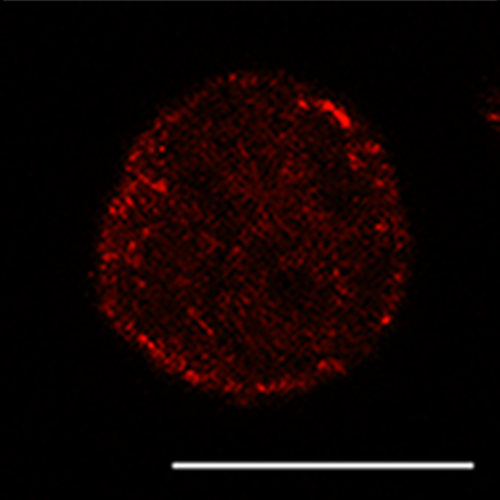

Supplement: Supplementary file 4 — Source data Fig. 3 [file 44319_2025_616_MOESM4_ESM.zip › 3C/Fig3C_D4_C1qC.tif]

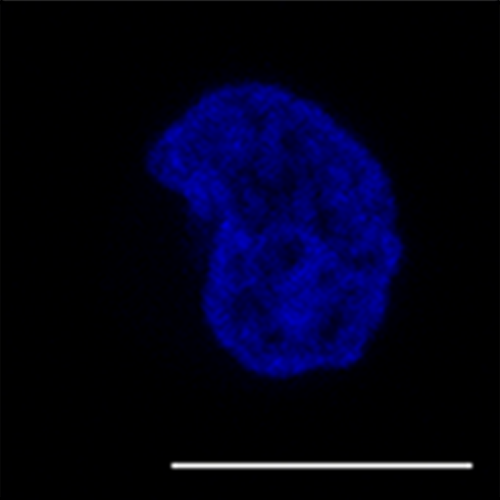

Supplement: Supplementary file 4 — Source data Fig. 3 [file 44319_2025_616_MOESM4_ESM.zip › 3C/Fig3C_D4_DAPI.tif]

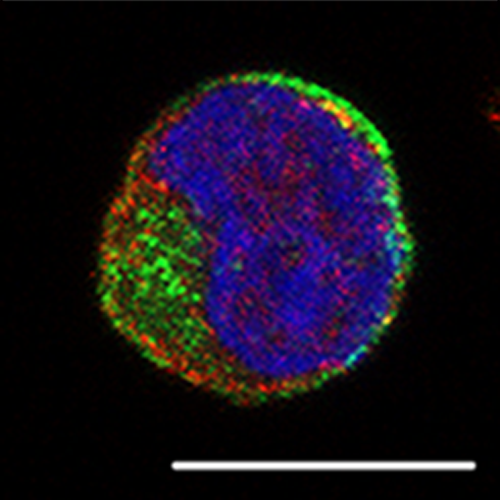

Supplement: Supplementary file 4 — Source data Fig. 3 [file 44319_2025_616_MOESM4_ESM.zip › 3C/Fig3C_D4_Merged.tif]

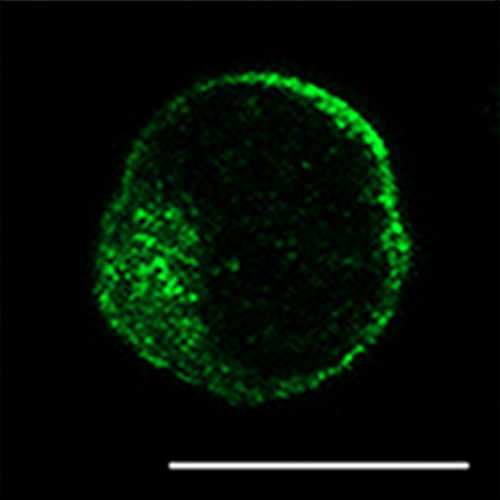

Supplement: Supplementary file 4 — Source data Fig. 3 [file 44319_2025_616_MOESM4_ESM.zip › 3C/Fig3C_D4_TRIM10alpha.tif]

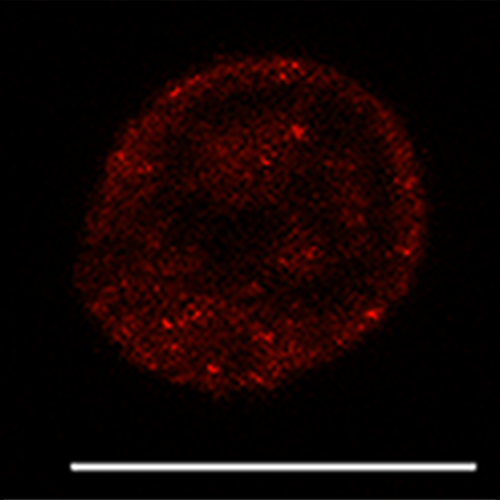

Supplement: Supplementary file 4 — Source data Fig. 3 [file 44319_2025_616_MOESM4_ESM.zip › 3C/Fig3C_D8_C1qC.tif]

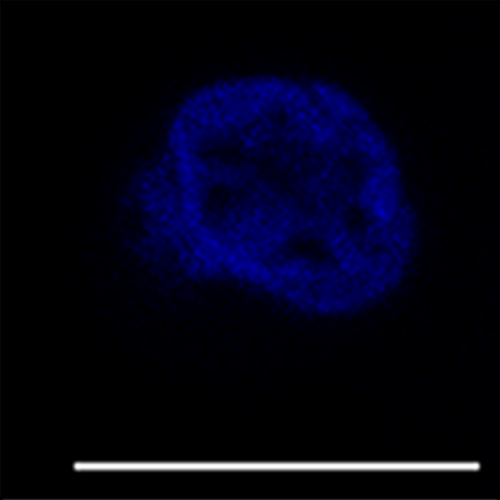

Supplement: Supplementary file 4 — Source data Fig. 3 [file 44319_2025_616_MOESM4_ESM.zip › 3C/Fig3C_D8_DAPI.tif]

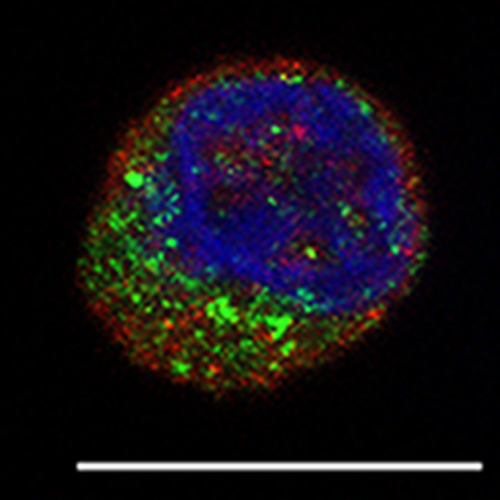

Supplement: Supplementary file 4 — Source data Fig. 3 [file 44319_2025_616_MOESM4_ESM.zip › 3C/Fig3C_D8_Merged.tif]

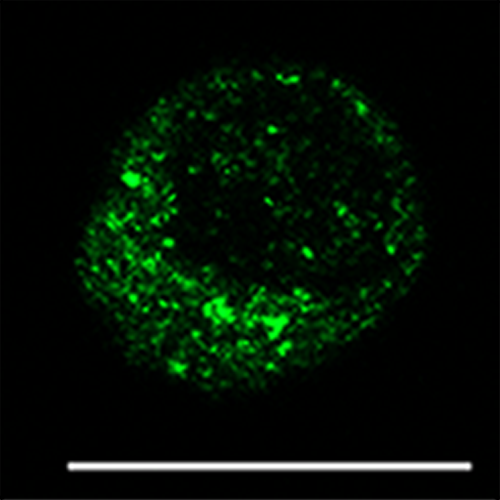

Supplement: Supplementary file 4 — Source data Fig. 3 [file 44319_2025_616_MOESM4_ESM.zip › 3C/Fig3C_D8_TRIM10alpha.tif]

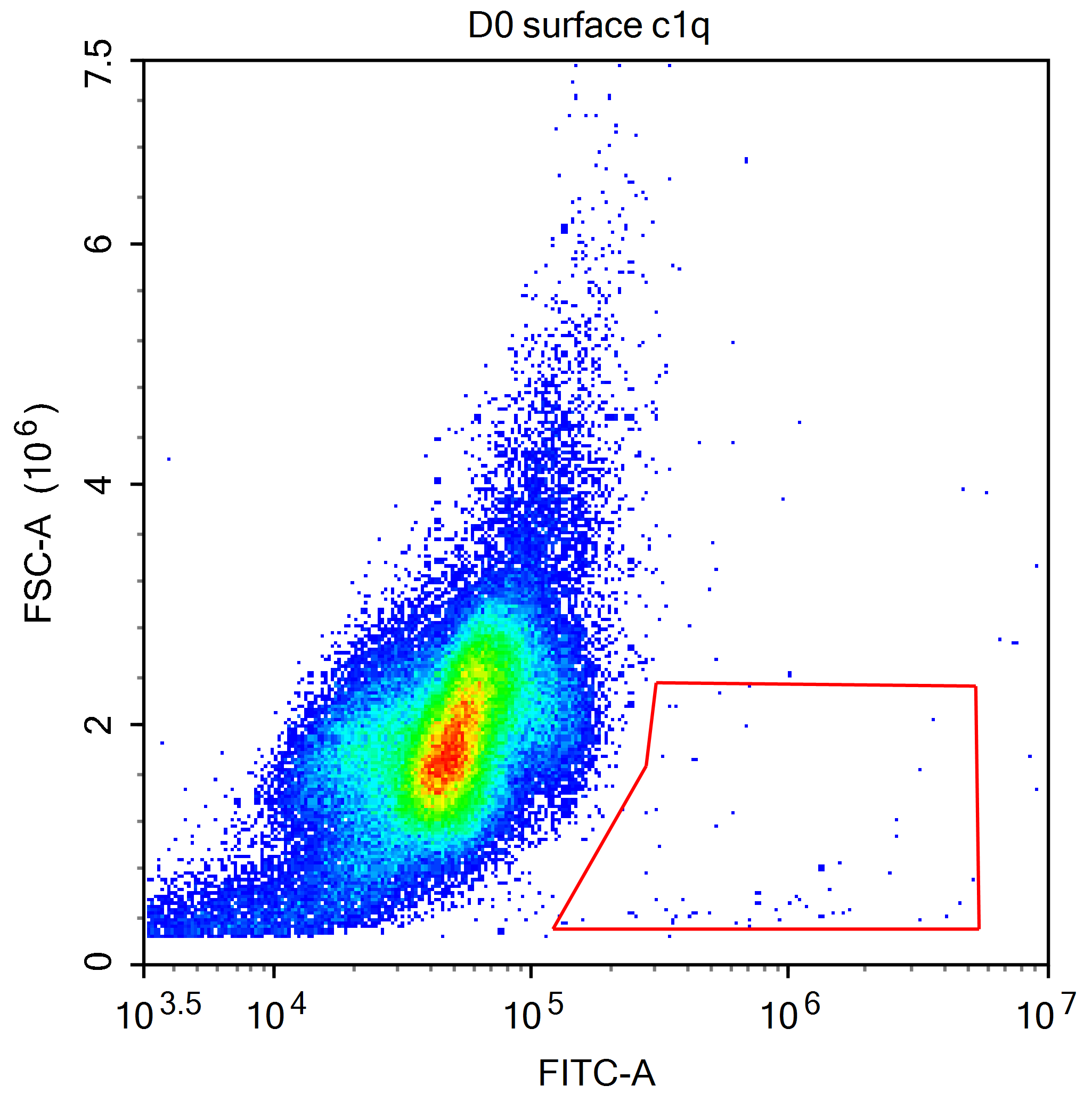

Supplement: Supplementary file 4 — Source data Fig. 3 [file 44319_2025_616_MOESM4_ESM.zip › 3D/Fig3D_D0_Anti-C1q_Surface.tiff]

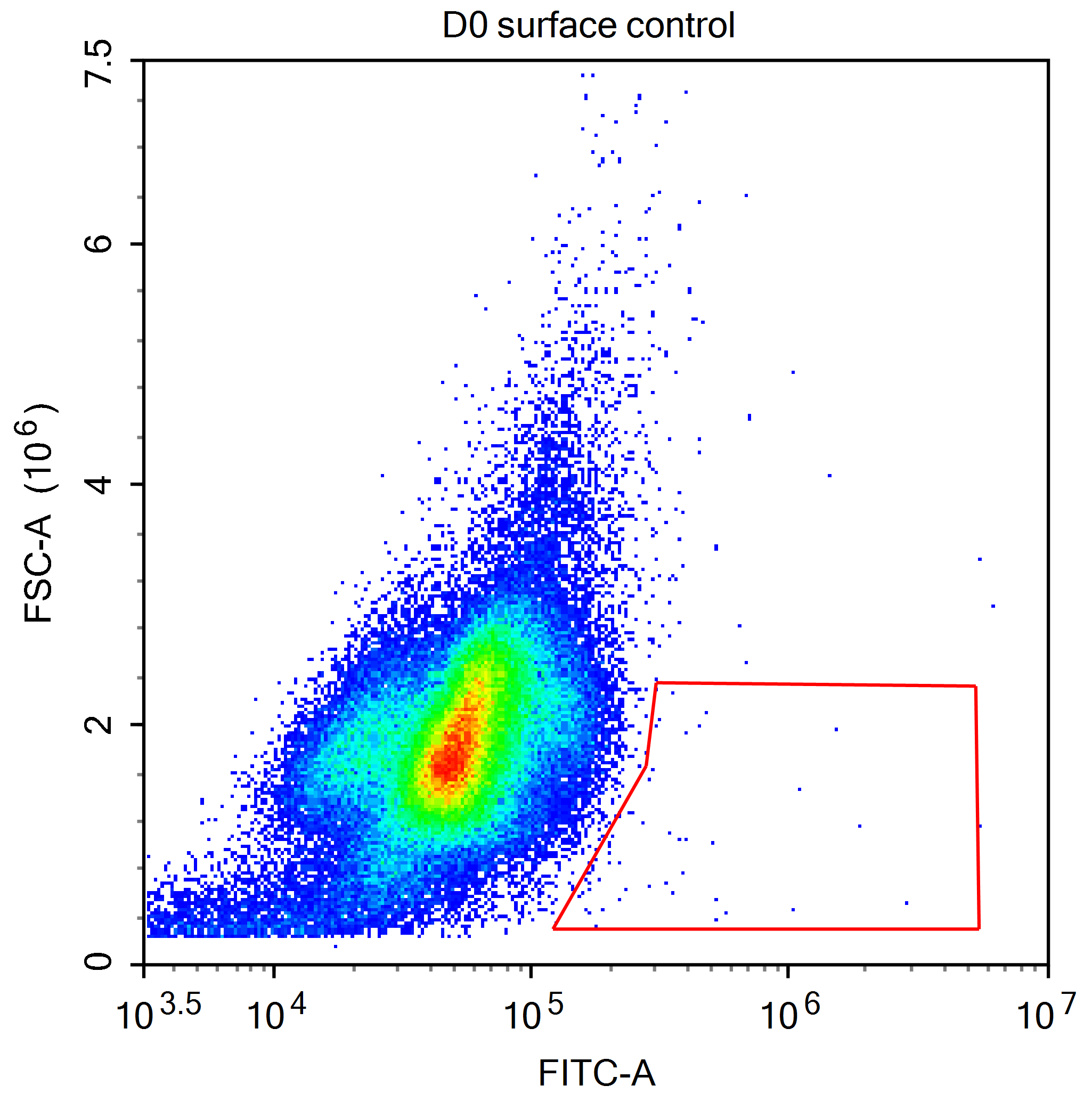

Supplement: Supplementary file 4 — Source data Fig. 3 [file 44319_2025_616_MOESM4_ESM.zip › 3D/Fig3D_D0_Isotype_Control_Surface.tiff]

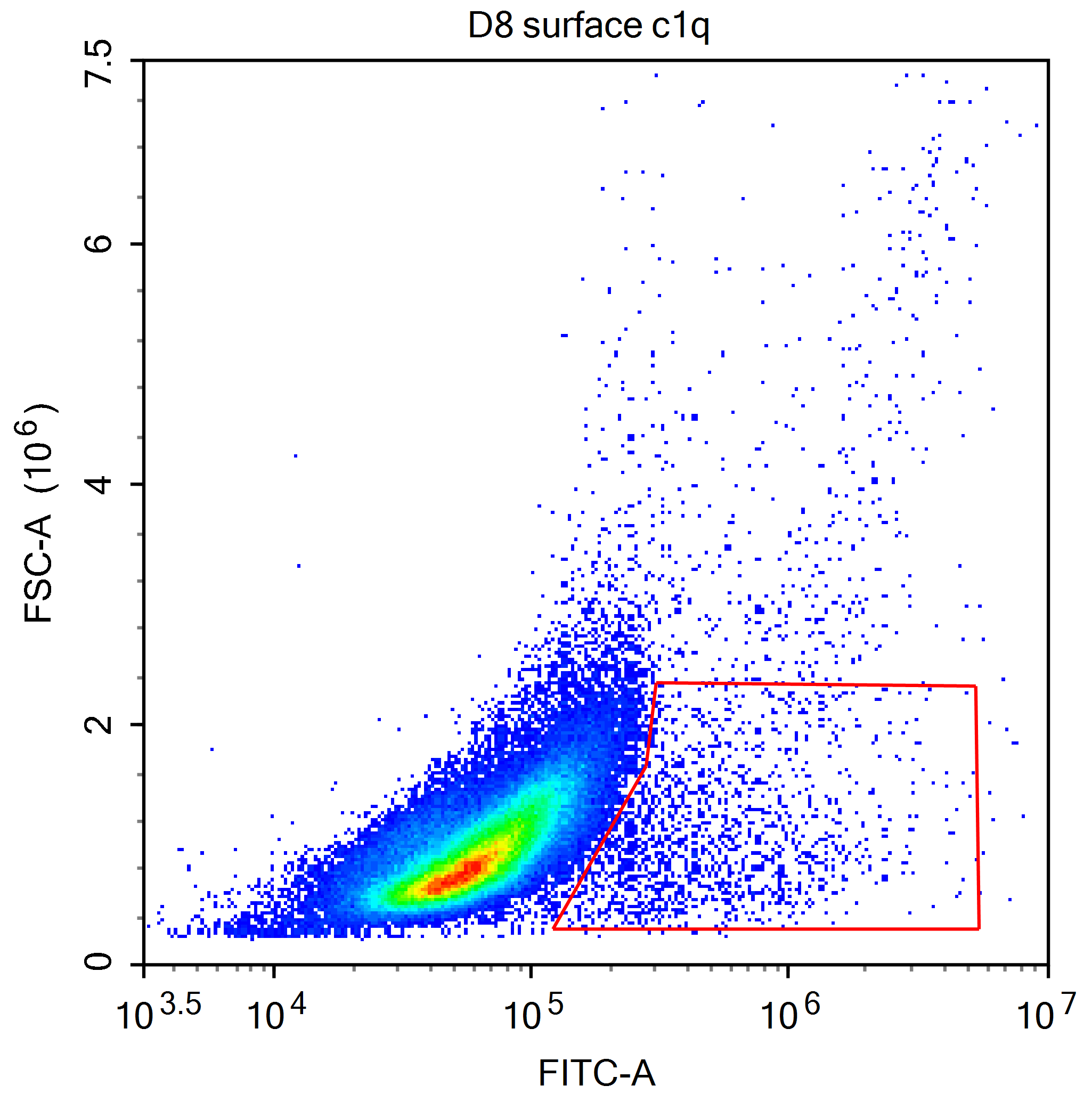

Supplement: Supplementary file 4 — Source data Fig. 3 [file 44319_2025_616_MOESM4_ESM.zip › 3D/Fig3D_D8_Anti-C1q_Surface.tiff]

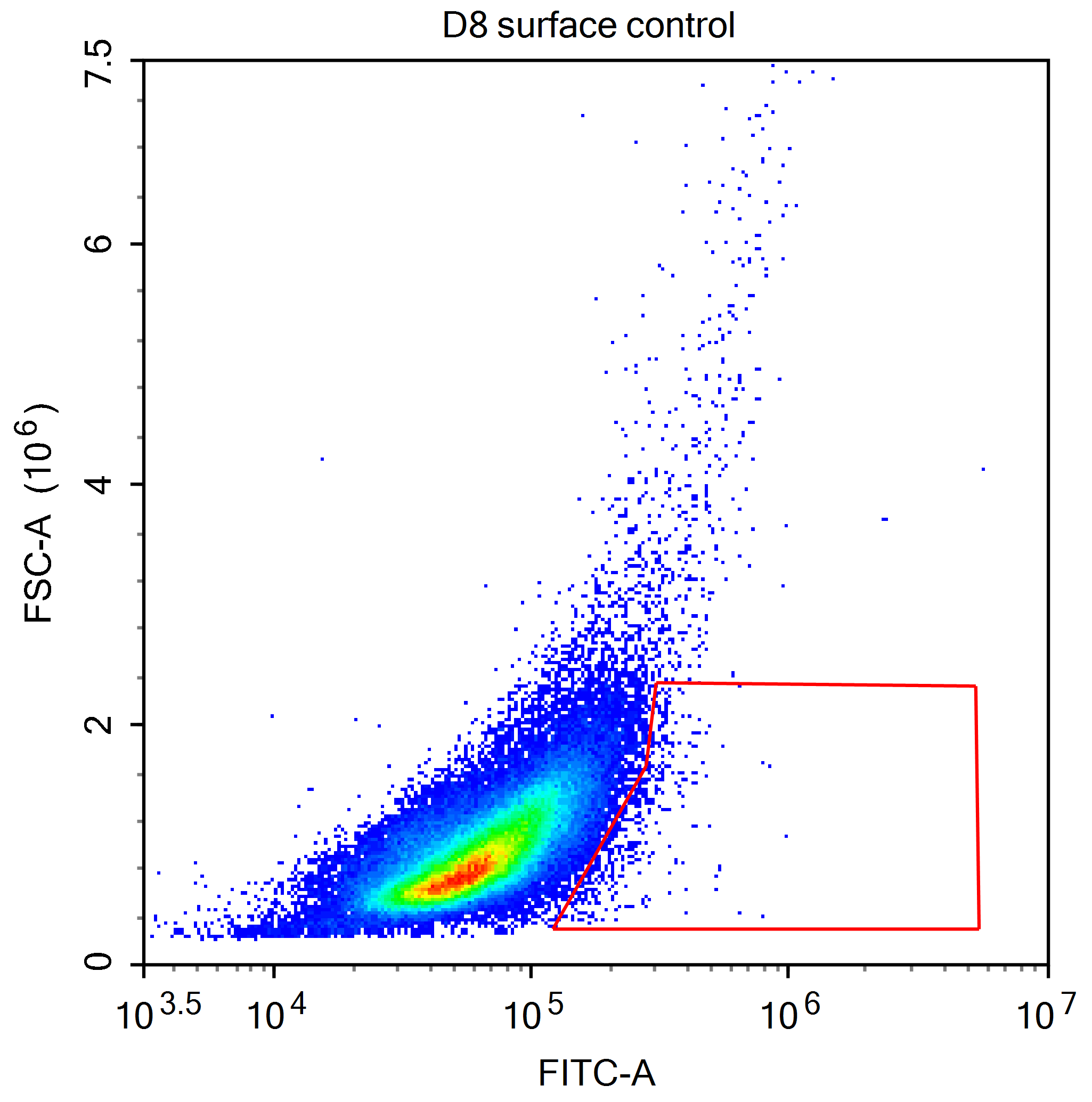

Supplement: Supplementary file 4 — Source data Fig. 3 [file 44319_2025_616_MOESM4_ESM.zip › 3D/Fig3D_D8_Isotype_Control_Surface.tiff]

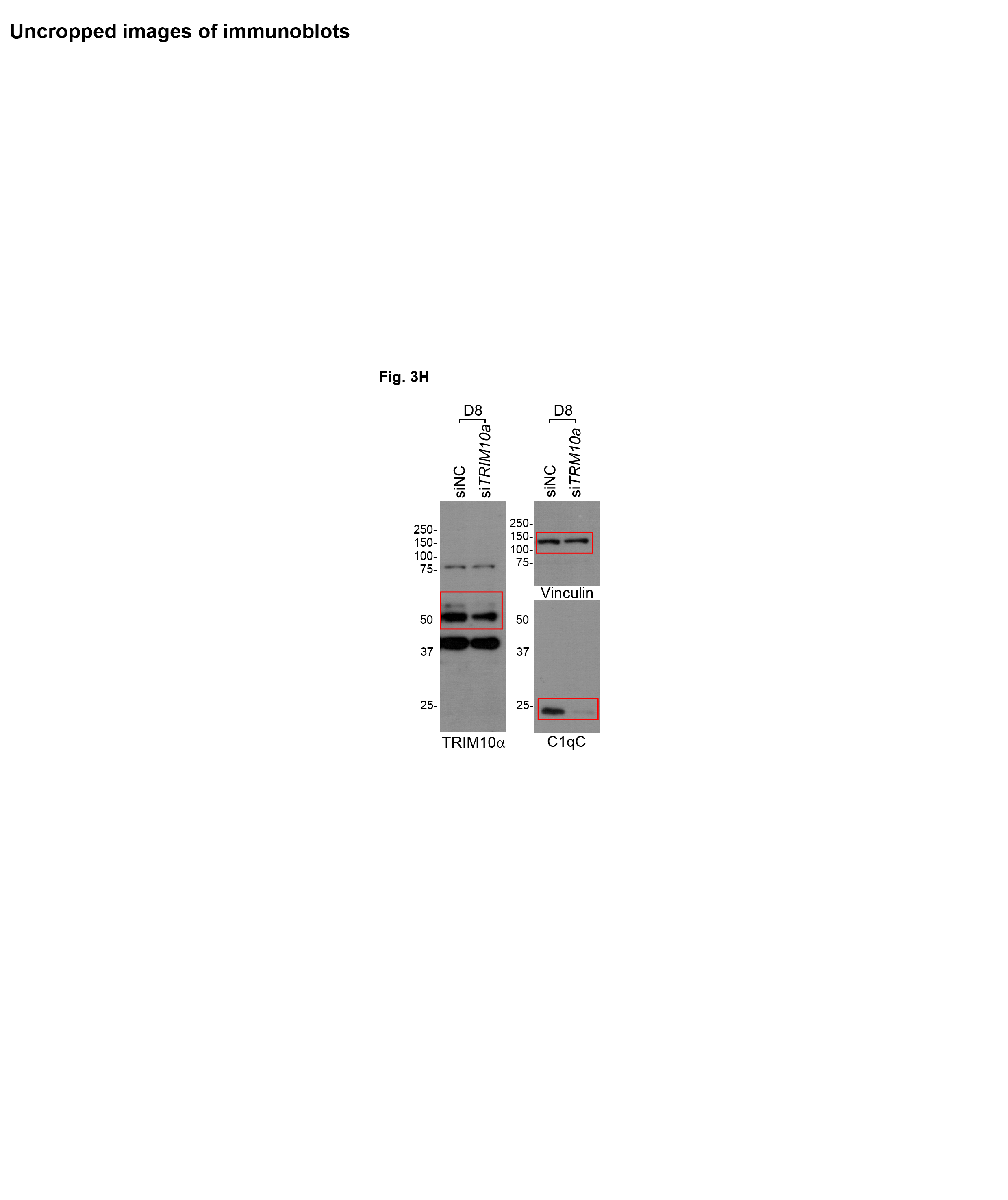

Supplement: Supplementary file 4 — Source data Fig. 3 [file 44319_2025_616_MOESM4_ESM.zip › 3H/Fig3H_Blot_data.tif]

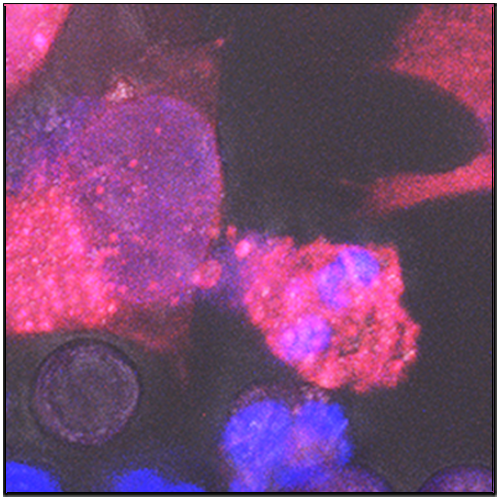

Supplement: Supplementary file 5 — Source data Fig. 4 [file 44319_2025_616_MOESM5_ESM.zip › 4B/Fig4B_coculture_2D.tif]

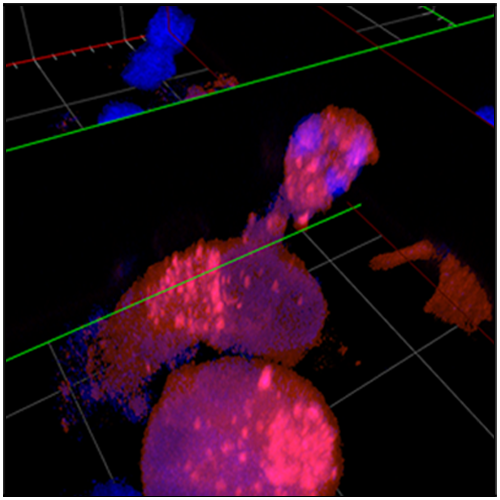

Supplement: Supplementary file 5 — Source data Fig. 4 [file 44319_2025_616_MOESM5_ESM.zip › 4B/Fig4B_coculture_3D.tif]

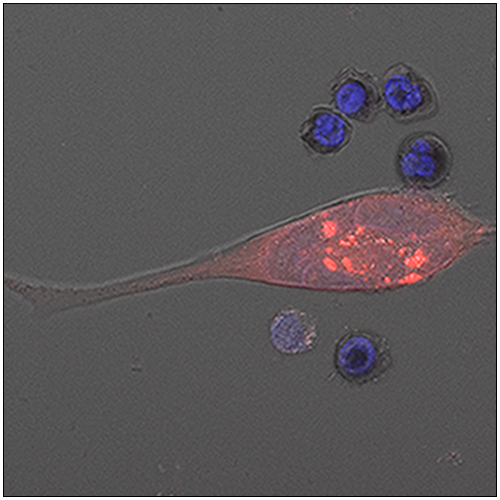

Supplement: Supplementary file 5 — Source data Fig. 4 [file 44319_2025_616_MOESM5_ESM.zip › 4D/Fig4D_coculture_C1qdpl.tif]

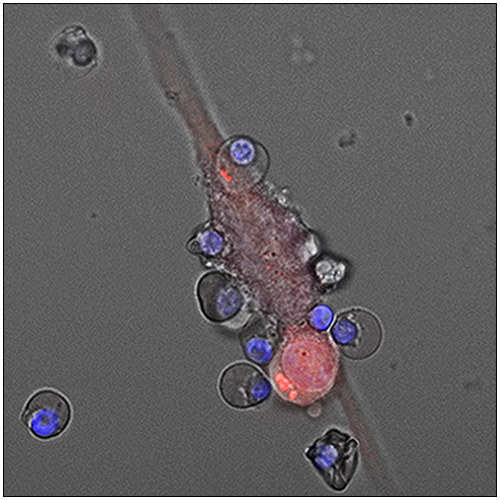

Supplement: Supplementary file 5 — Source data Fig. 4 [file 44319_2025_616_MOESM5_ESM.zip › 4D/Fig4D_coculture_Normal.tif]

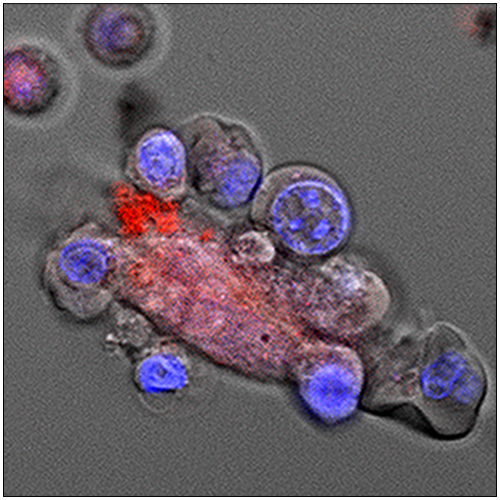

Supplement: Supplementary file 5 — Source data Fig. 4 [file 44319_2025_616_MOESM5_ESM.zip › 4E/Fig4E_coculture_siNC.tif]

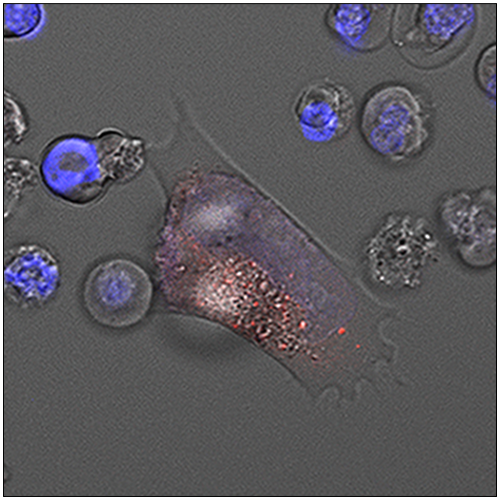

Supplement: Supplementary file 5 — Source data Fig. 4 [file 44319_2025_616_MOESM5_ESM.zip › 4E/Fig4E_coculture_siTRIM10a.tif]

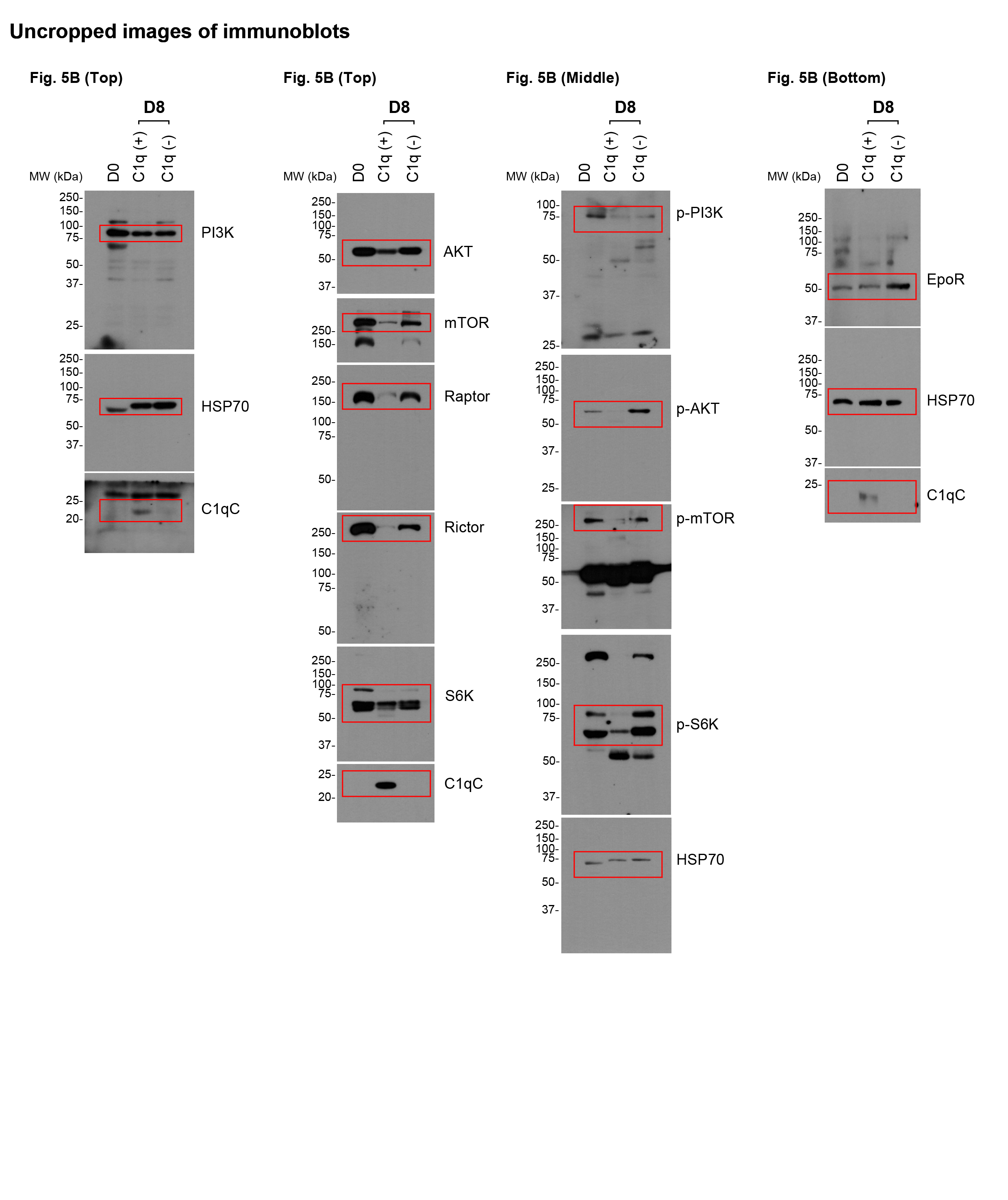

Supplement: Supplementary file 6 — Source data Fig. 5 [file 44319_2025_616_MOESM6_ESM.zip › 5B/Fig5B_Blot_data.tif]

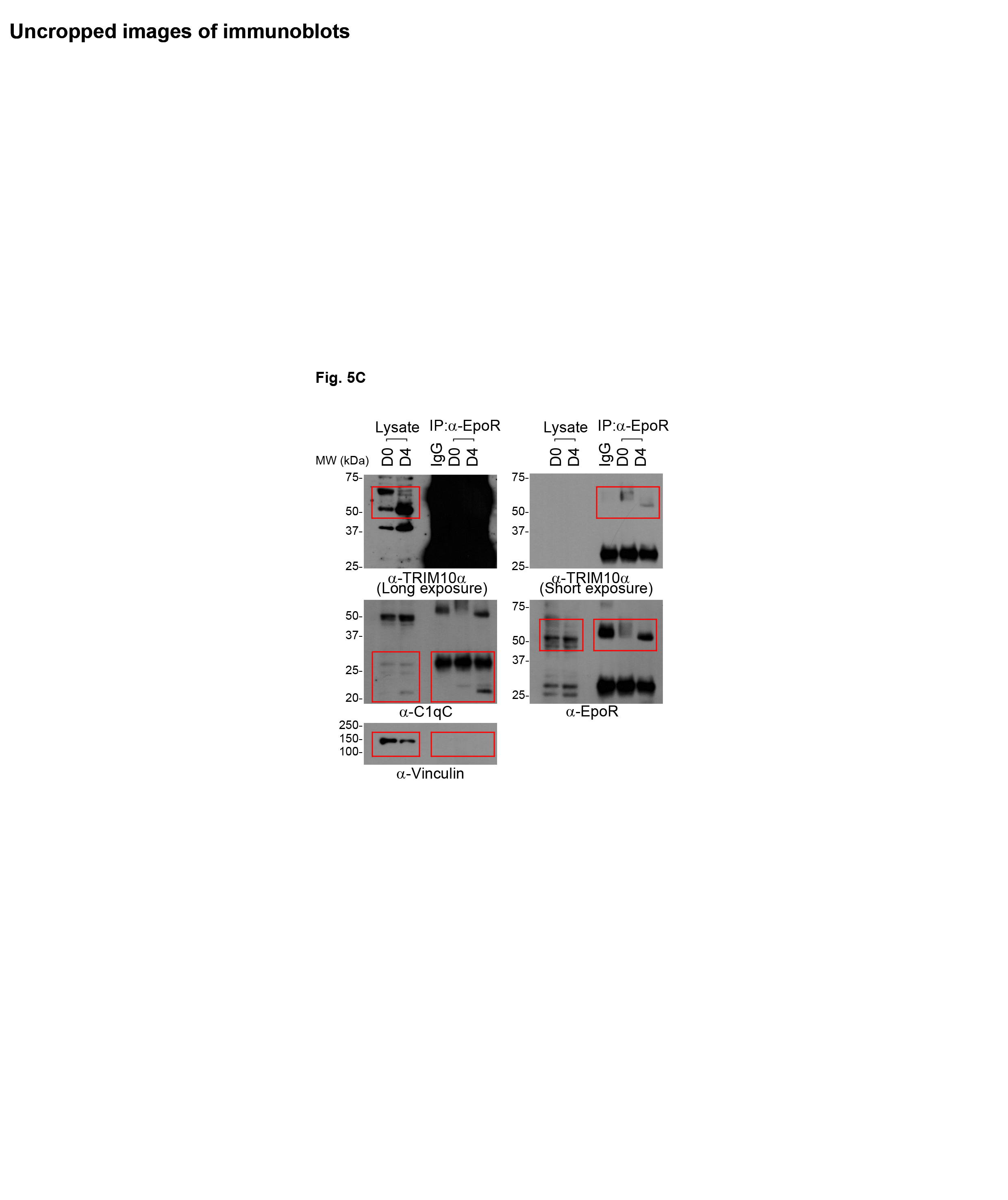

Supplement: Supplementary file 6 — Source data Fig. 5 [file 44319_2025_616_MOESM6_ESM.zip › 5C/Fig5C_Blot_data.tif]

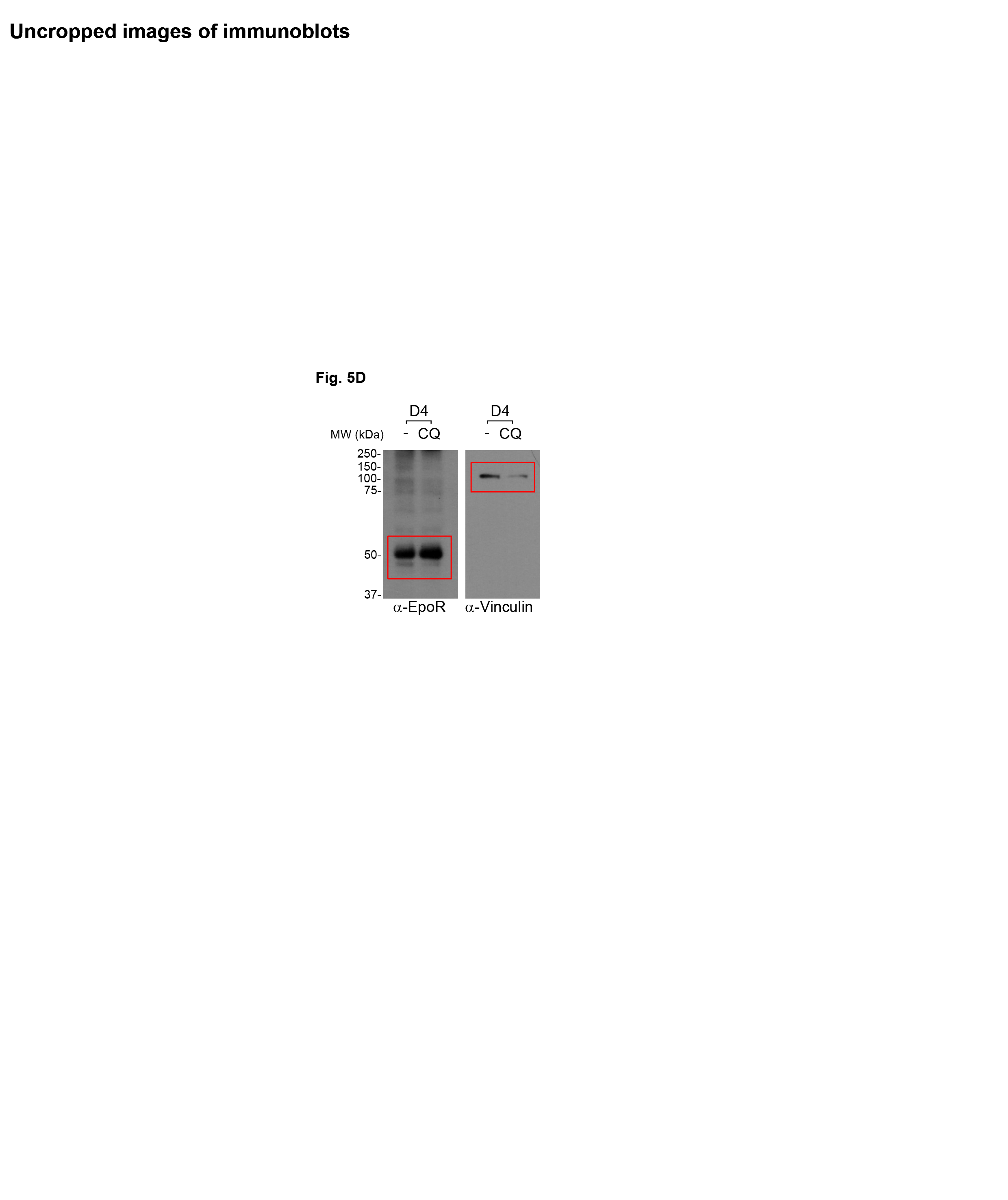

Supplement: Supplementary file 6 — Source data Fig. 5 [file 44319_2025_616_MOESM6_ESM.zip › 5D/Fig5D_Blot_data.tif]

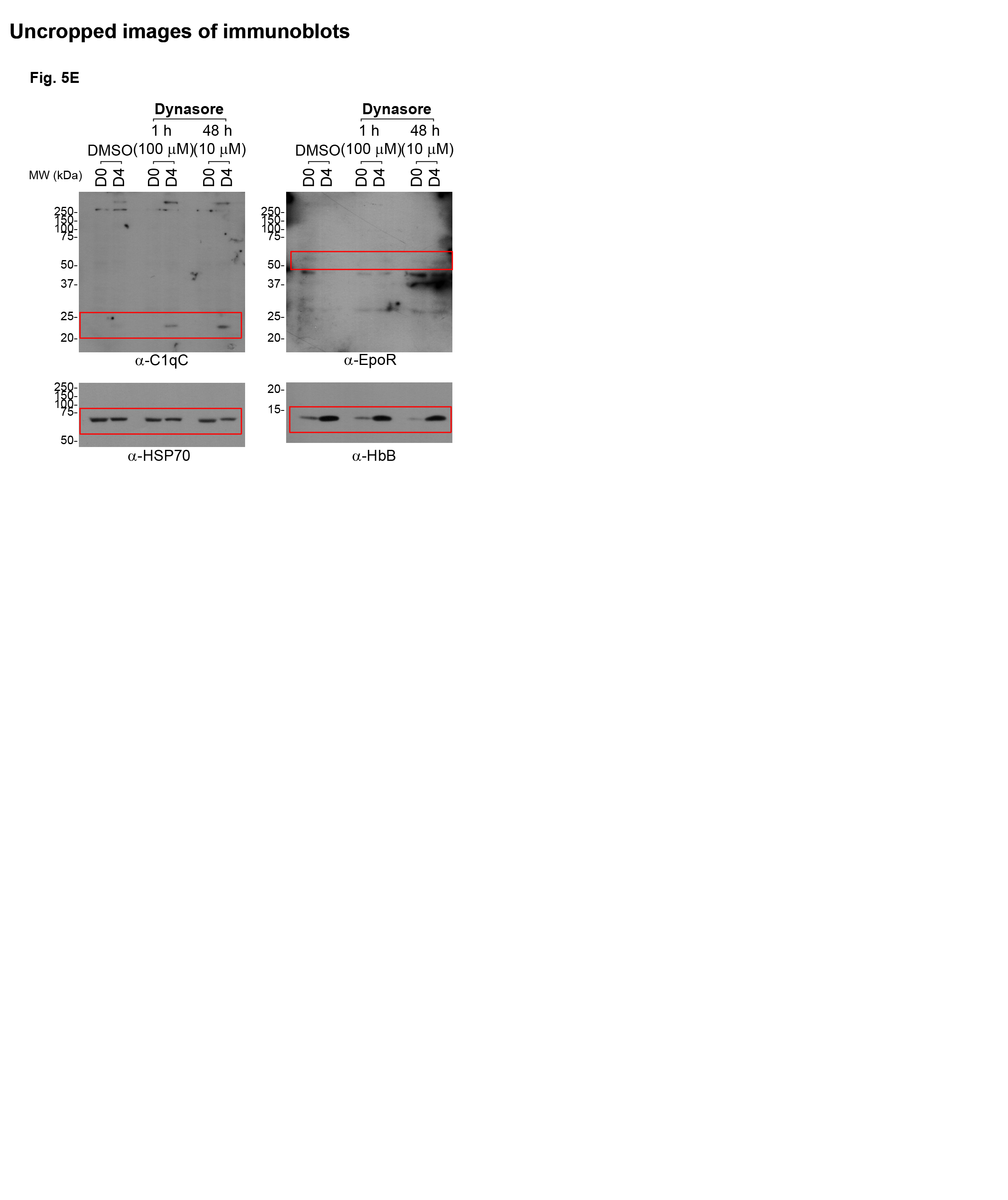

Supplement: Supplementary file 6 — Source data Fig. 5 [file 44319_2025_616_MOESM6_ESM.zip › 5E/Fig5E_Blot_data.tif]

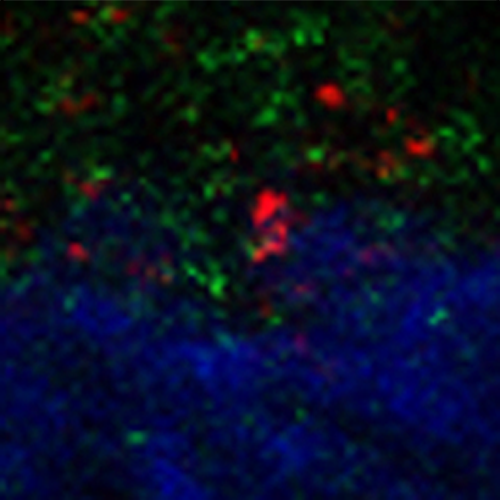

Supplement: Supplementary file 6 — Source data Fig. 5 [file 44319_2025_616_MOESM6_ESM.zip › 5F/Fig5F_D0_Enlarged_image1.tif]

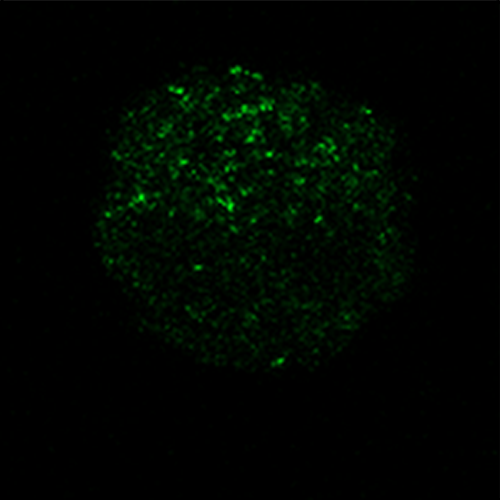

Supplement: Supplementary file 6 — Source data Fig. 5 [file 44319_2025_616_MOESM6_ESM.zip › 5F/Fig5F_D0_EpoR.tif]

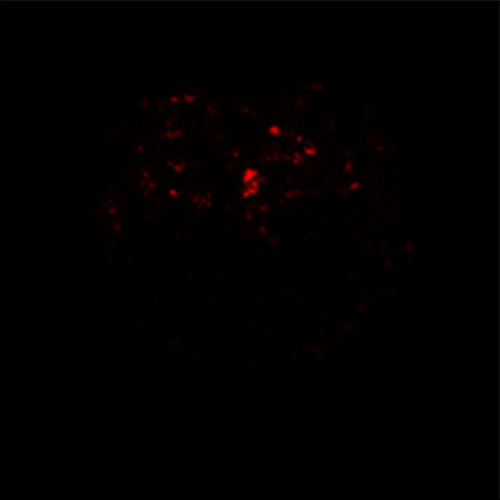

Supplement: Supplementary file 6 — Source data Fig. 5 [file 44319_2025_616_MOESM6_ESM.zip › 5F/Fig5F_D0_LAMP1.tif]

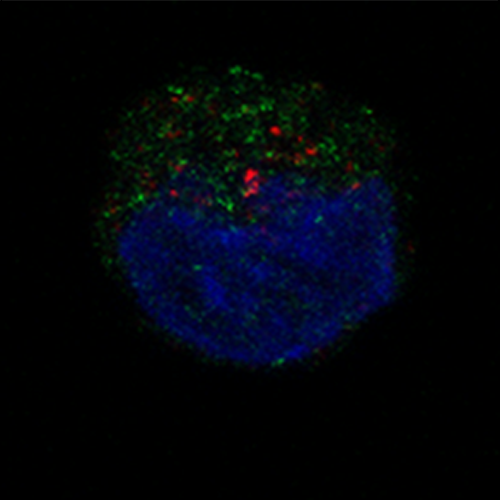

Supplement: Supplementary file 6 — Source data Fig. 5 [file 44319_2025_616_MOESM6_ESM.zip › 5F/Fig5F_D0_Merged.tif]

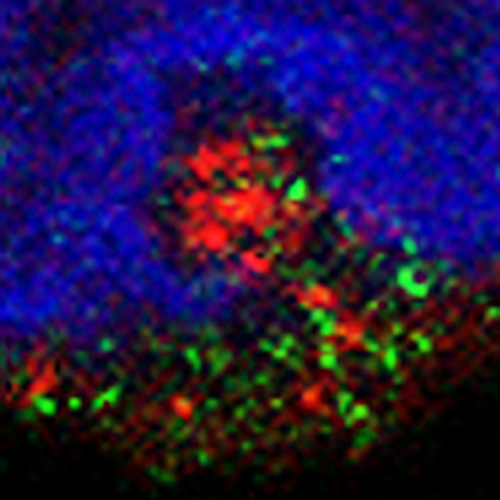

Supplement: Supplementary file 6 — Source data Fig. 5 [file 44319_2025_616_MOESM6_ESM.zip › 5F/Fig5F_D4_C1q_minus_Enlarged_image3.tif]

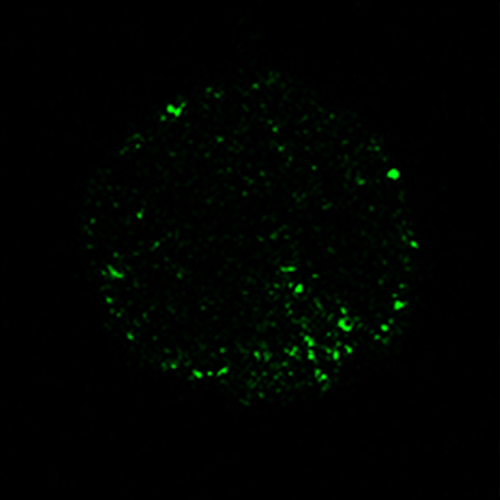

Supplement: Supplementary file 6 — Source data Fig. 5 [file 44319_2025_616_MOESM6_ESM.zip › 5F/Fig5F_D4_C1q_minus_EpoR.tif]

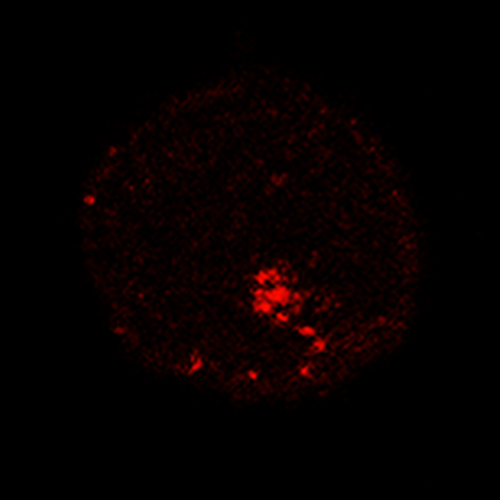

Supplement: Supplementary file 6 — Source data Fig. 5 [file 44319_2025_616_MOESM6_ESM.zip › 5F/Fig5F_D4_C1q_minus_LAMP1.tif]

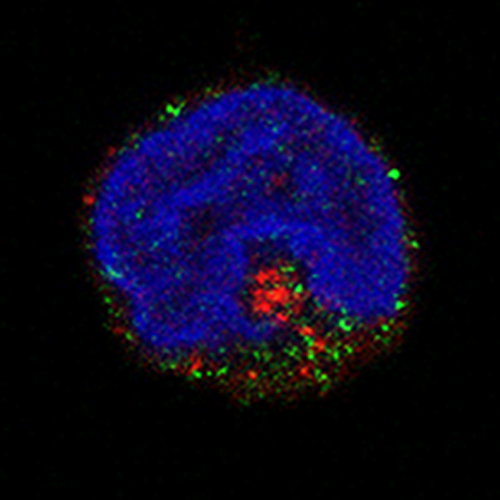

Supplement: Supplementary file 6 — Source data Fig. 5 [file 44319_2025_616_MOESM6_ESM.zip › 5F/Fig5F_D4_C1q_minus_Merged.tif]

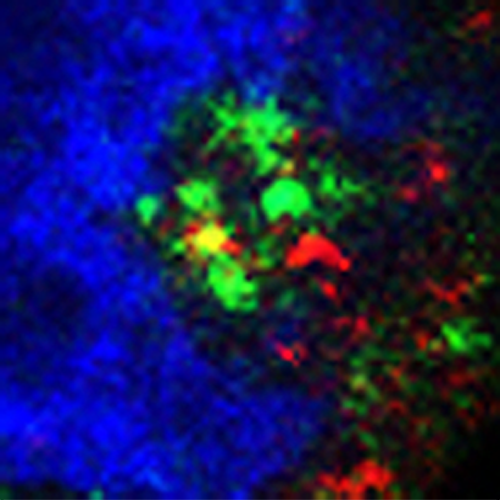

Supplement: Supplementary file 6 — Source data Fig. 5 [file 44319_2025_616_MOESM6_ESM.zip › 5F/Fig5F_D4_C1q_plus_Enlarged_image2.tif]

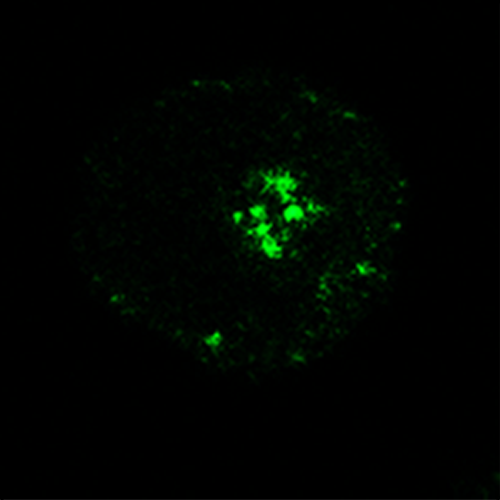

Supplement: Supplementary file 6 — Source data Fig. 5 [file 44319_2025_616_MOESM6_ESM.zip › 5F/Fig5F_D4_C1q_plus_EpoR.tif]

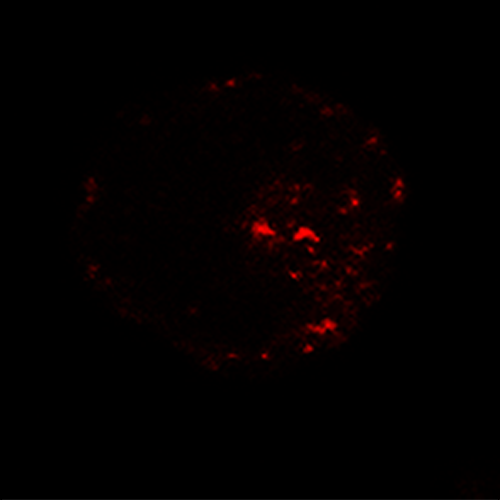

Supplement: Supplementary file 6 — Source data Fig. 5 [file 44319_2025_616_MOESM6_ESM.zip › 5F/Fig5F_D4_C1q_plus_LAMP1.tif]

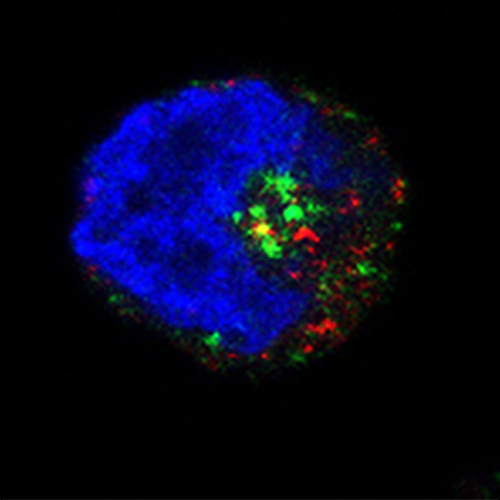

Supplement: Supplementary file 6 — Source data Fig. 5 [file 44319_2025_616_MOESM6_ESM.zip › 5F/Fig5F_D4_C1q_plus_Merged.tif]

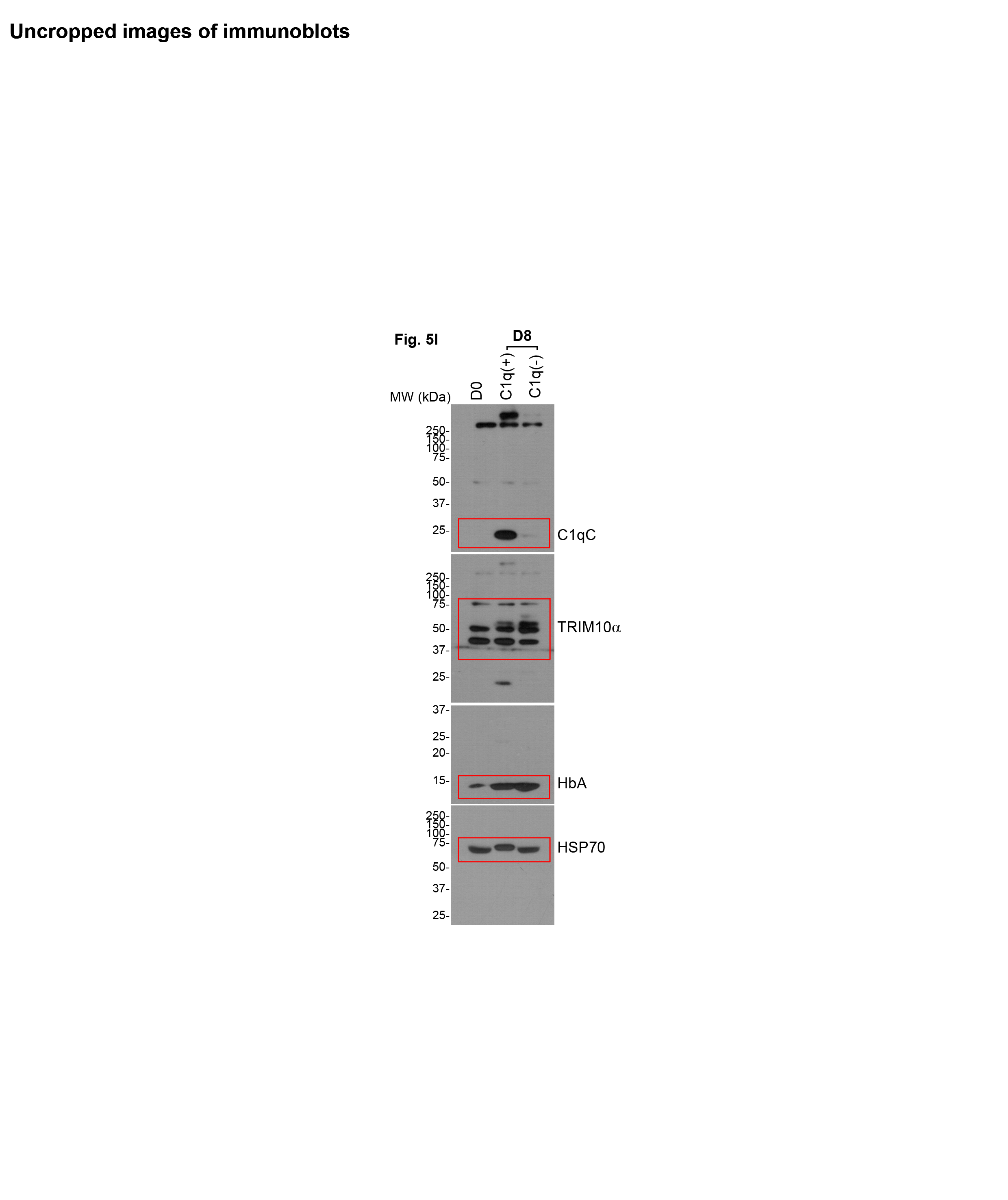

Supplement: Supplementary file 6 — Source data Fig. 5 [file 44319_2025_616_MOESM6_ESM.zip › 5I/Fig5I_Blot_data.tif]

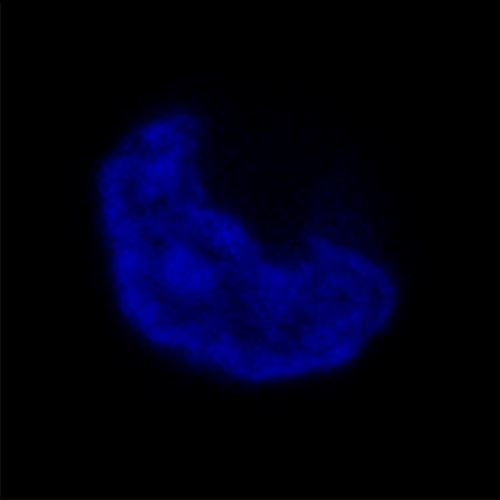

Supplement: Supplementary file 7 — Source data Fig. 6 [file 44319_2025_616_MOESM7_ESM.zip › 6A/Fig6A_D8_DAPI.tif]

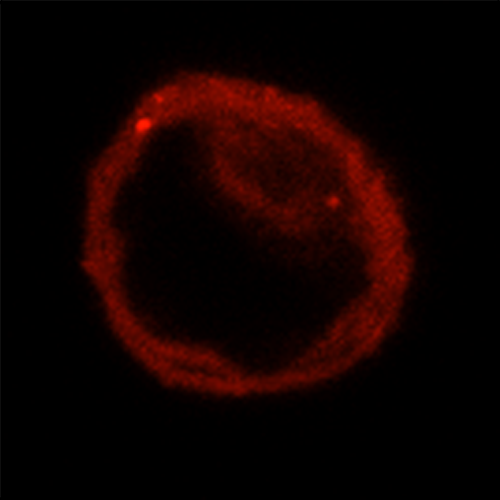

Supplement: Supplementary file 7 — Source data Fig. 6 [file 44319_2025_616_MOESM7_ESM.zip › 6A/Fig6A_D8_HbA.tif]

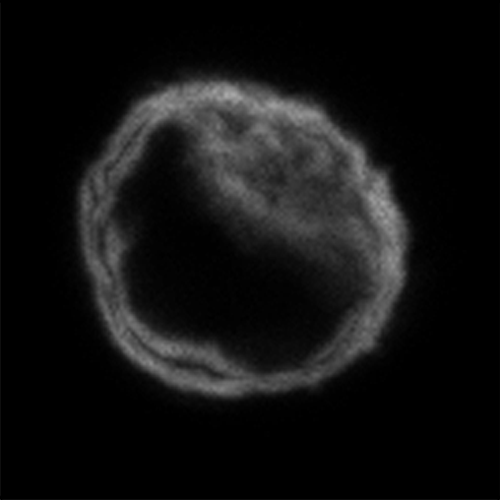

Supplement: Supplementary file 7 — Source data Fig. 6 [file 44319_2025_616_MOESM7_ESM.zip › 6A/Fig6A_D8_HbB.tif]

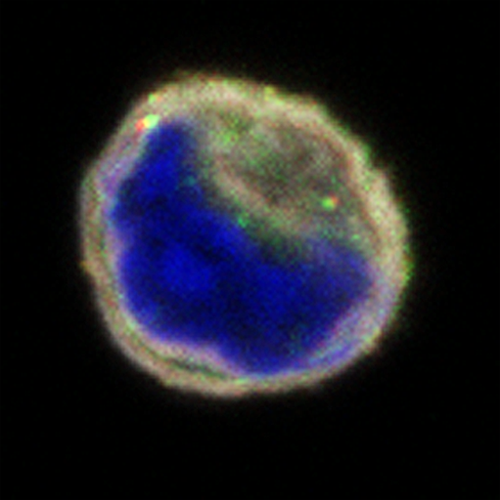

Supplement: Supplementary file 7 — Source data Fig. 6 [file 44319_2025_616_MOESM7_ESM.zip › 6A/Fig6A_D8_Merged.tif]

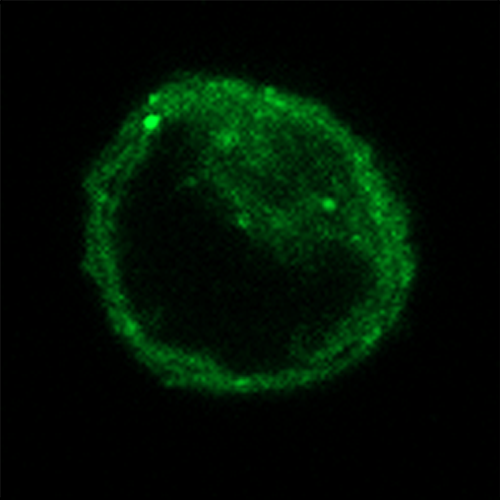

Supplement: Supplementary file 7 — Source data Fig. 6 [file 44319_2025_616_MOESM7_ESM.zip › 6A/Fig6A_D8_TRIM10alpha.tif]

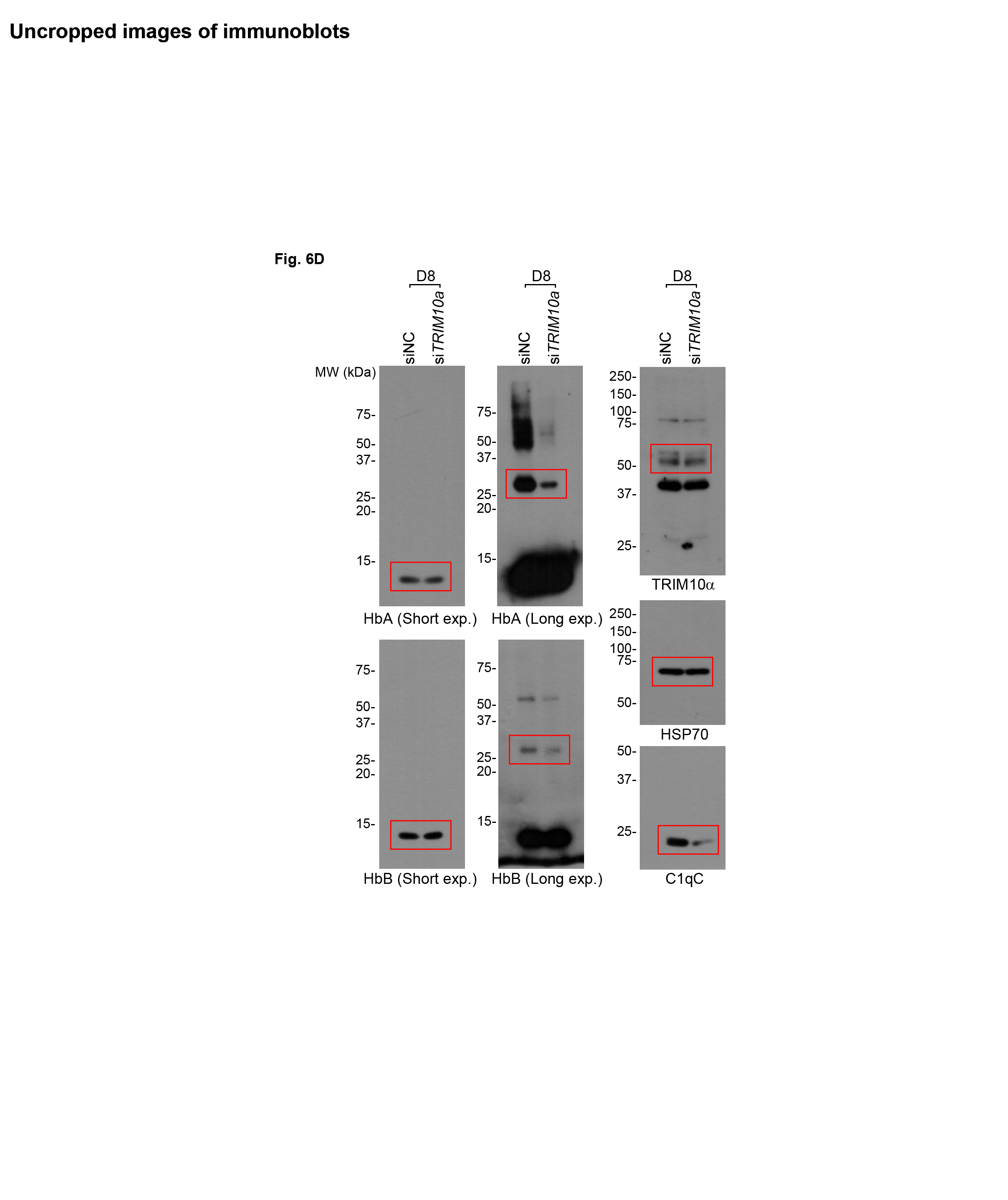

Supplement: Supplementary file 7 — Source data Fig. 6 [file 44319_2025_616_MOESM7_ESM.zip › 6D/Fig6D_Blot_data.tif]

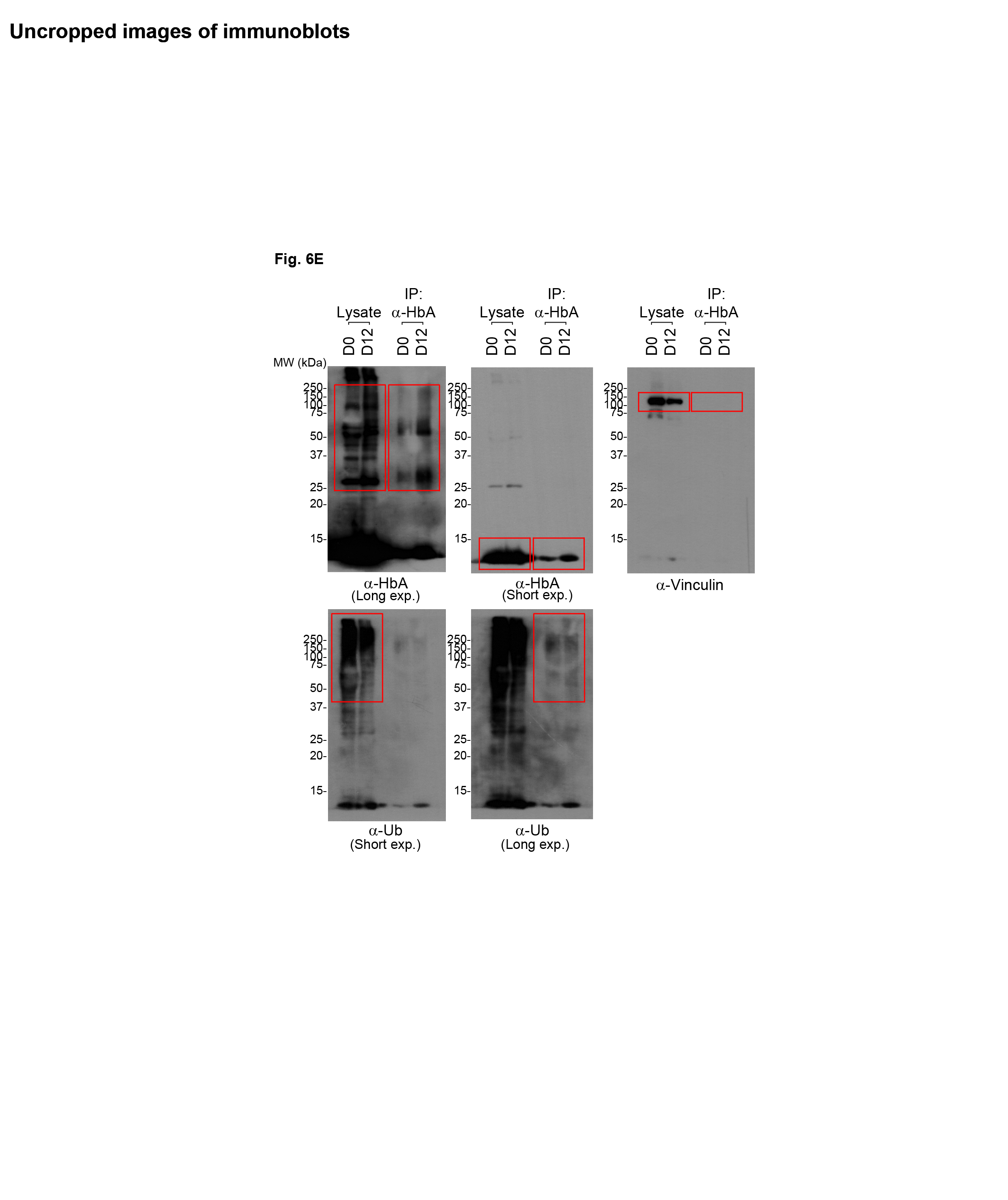

Supplement: Supplementary file 7 — Source data Fig. 6 [file 44319_2025_616_MOESM7_ESM.zip › 6E/Fig6E_Blot_data.tif]

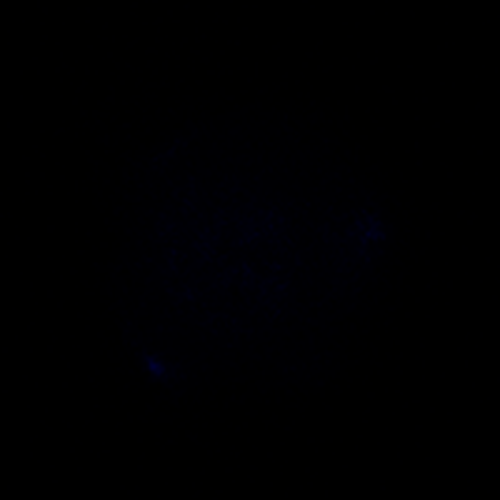

Supplement: Supplementary file 7 — Source data Fig. 6 [file 44319_2025_616_MOESM7_ESM.zip › 6F/Fig6F_D12RETICULO_DAPI.tif]
