# Supplementary figures and images for: Stage-specific TRIM10 expression regulates erythroid maturation (part 2 of 3)
Source: EMBO Rep. 2025 Oct 30;26(23):5982–6014. doi: 10.1038/s44319-025-00616-0 (PMC12678476; doi:10.1038/s44319-025-00616-0)

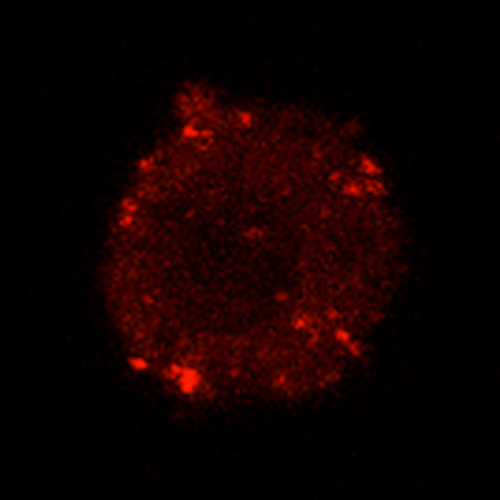

Supplement: Supplementary file 7 — Source data Fig. 6 [file 44319_2025_616_MOESM7_ESM.zip › 6F/Fig6F_D12RETICULO_HbA.tif]

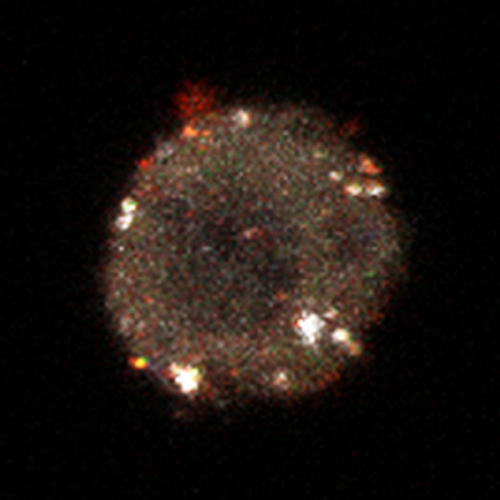

Supplement: Supplementary file 7 — Source data Fig. 6 [file 44319_2025_616_MOESM7_ESM.zip › 6F/Fig6F_D12RETICULO_merged.tif]

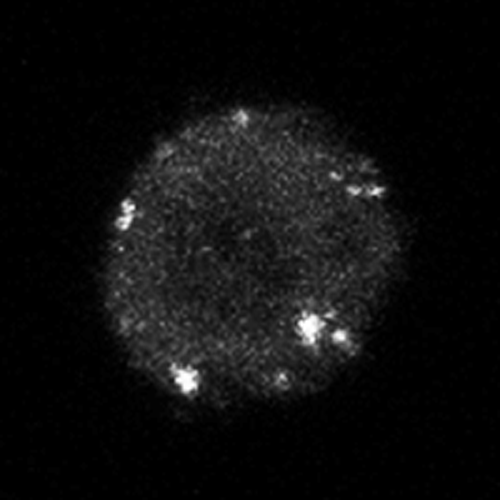

Supplement: Supplementary file 7 — Source data Fig. 6 [file 44319_2025_616_MOESM7_ESM.zip › 6F/Fig6F_D12RETICULO_P62.tif]

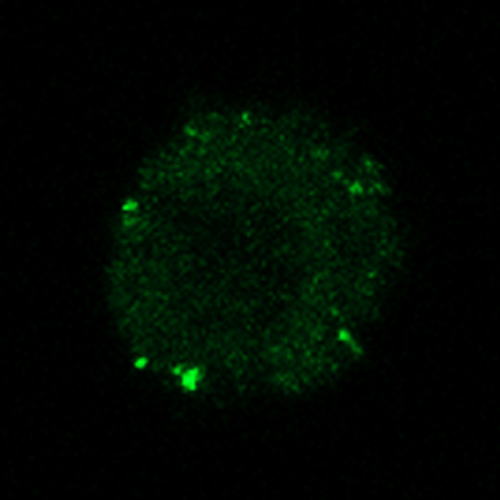

Supplement: Supplementary file 7 — Source data Fig. 6 [file 44319_2025_616_MOESM7_ESM.zip › 6F/Fig6F_D12RETICULO_TRIM10alpha.tif]

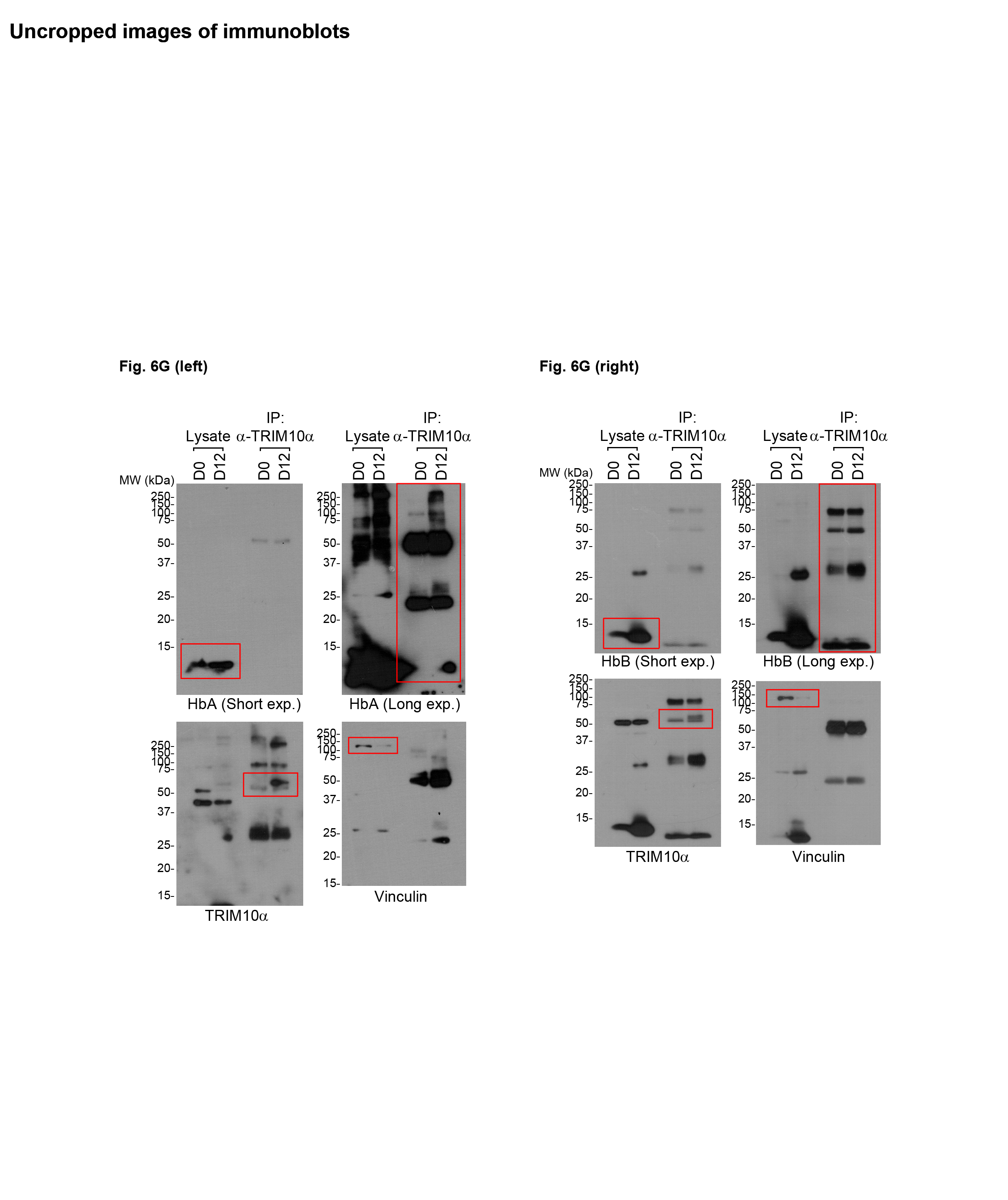

Supplement: Supplementary file 7 — Source data Fig. 6 [file 44319_2025_616_MOESM7_ESM.zip › 6G/Fig6G_Blot_data.tif]

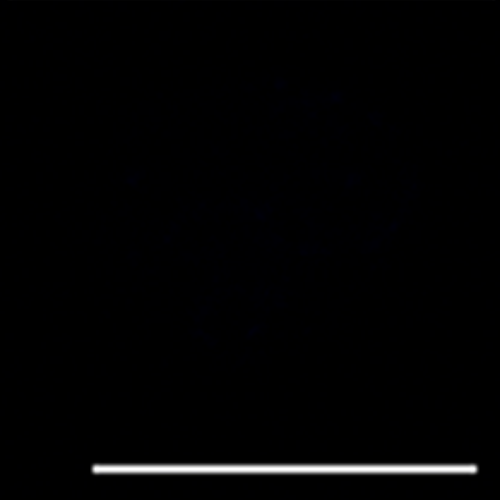

Supplement: Supplementary file 7 — Source data Fig. 6 [file 44319_2025_616_MOESM7_ESM.zip › 6H/Fig6H_D12RETICULO_siNC_DAPI.tif]

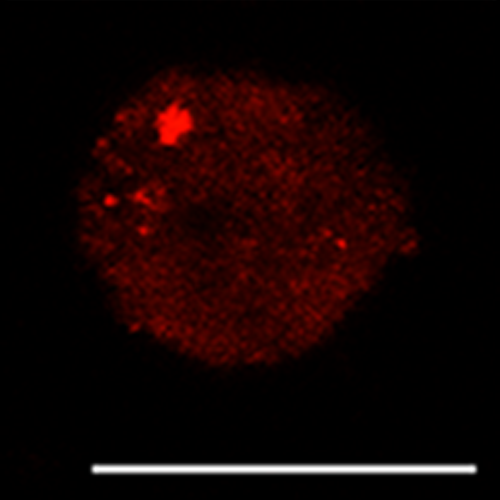

Supplement: Supplementary file 7 — Source data Fig. 6 [file 44319_2025_616_MOESM7_ESM.zip › 6H/Fig6H_D12RETICULO_siNC_HbA.tif]

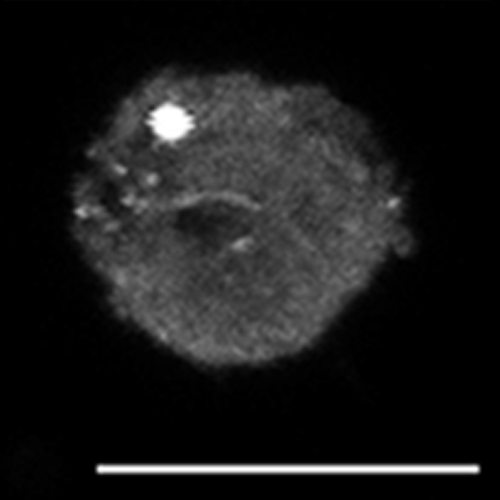

Supplement: Supplementary file 7 — Source data Fig. 6 [file 44319_2025_616_MOESM7_ESM.zip › 6H/Fig6H_D12RETICULO_siNC_HbB.tif]

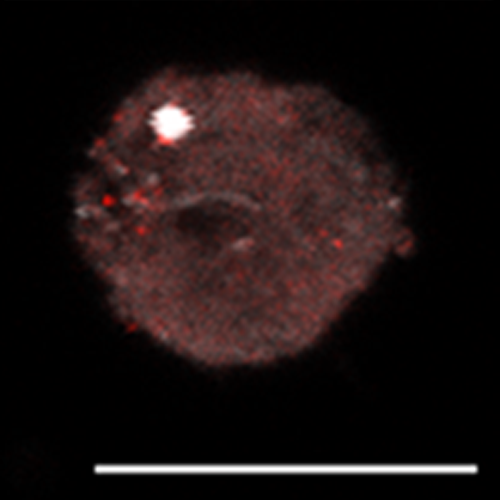

Supplement: Supplementary file 7 — Source data Fig. 6 [file 44319_2025_616_MOESM7_ESM.zip › 6H/Fig6H_D12RETICULO_siNC_Merged.tif]

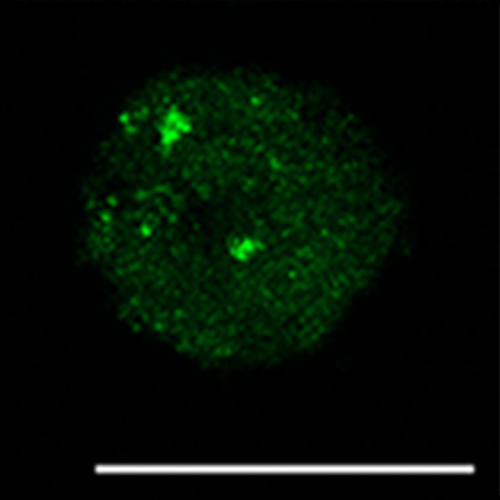

Supplement: Supplementary file 7 — Source data Fig. 6 [file 44319_2025_616_MOESM7_ESM.zip › 6H/Fig6H_D12RETICULO_siNC_TRIM10alpha.tif]

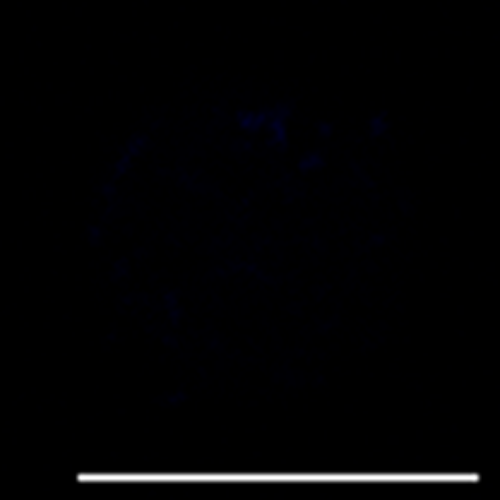

Supplement: Supplementary file 7 — Source data Fig. 6 [file 44319_2025_616_MOESM7_ESM.zip › 6H/Fig6H_D12RETICULO_siTRIM10a_DAPI.tif]

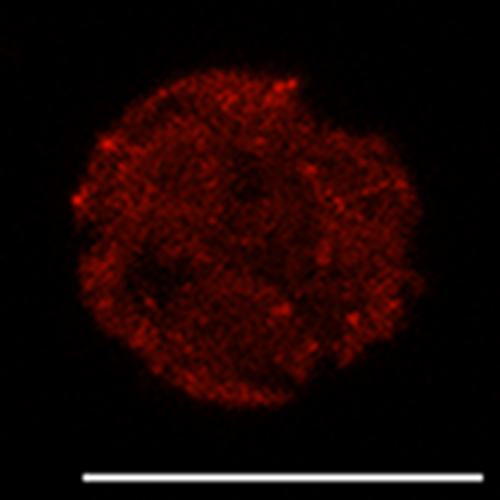

Supplement: Supplementary file 7 — Source data Fig. 6 [file 44319_2025_616_MOESM7_ESM.zip › 6H/Fig6H_D12RETICULO_siTRIM10a_HbA.tif]

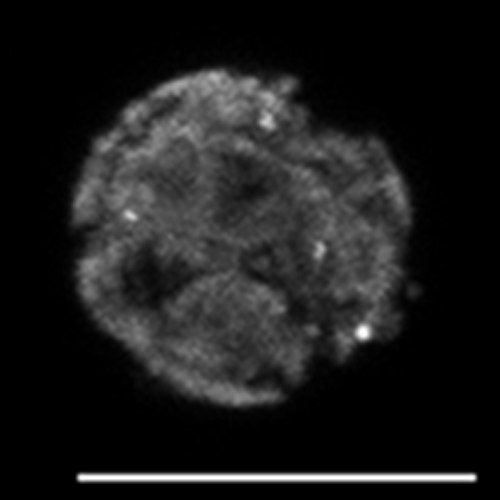

Supplement: Supplementary file 7 — Source data Fig. 6 [file 44319_2025_616_MOESM7_ESM.zip › 6H/Fig6H_D12RETICULO_siTRIM10a_HbB.tif]

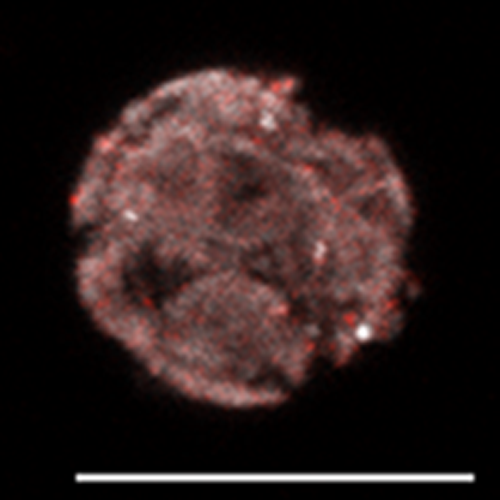

Supplement: Supplementary file 7 — Source data Fig. 6 [file 44319_2025_616_MOESM7_ESM.zip › 6H/Fig6H_D12RETICULO_siTRIM10a_Merged.tif]

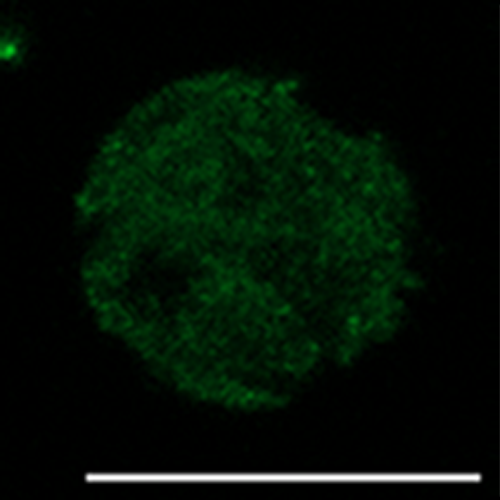

Supplement: Supplementary file 7 — Source data Fig. 6 [file 44319_2025_616_MOESM7_ESM.zip › 6H/Fig6H_D12RETICULO_siTRIM10a_TRIM10alpha.tif]

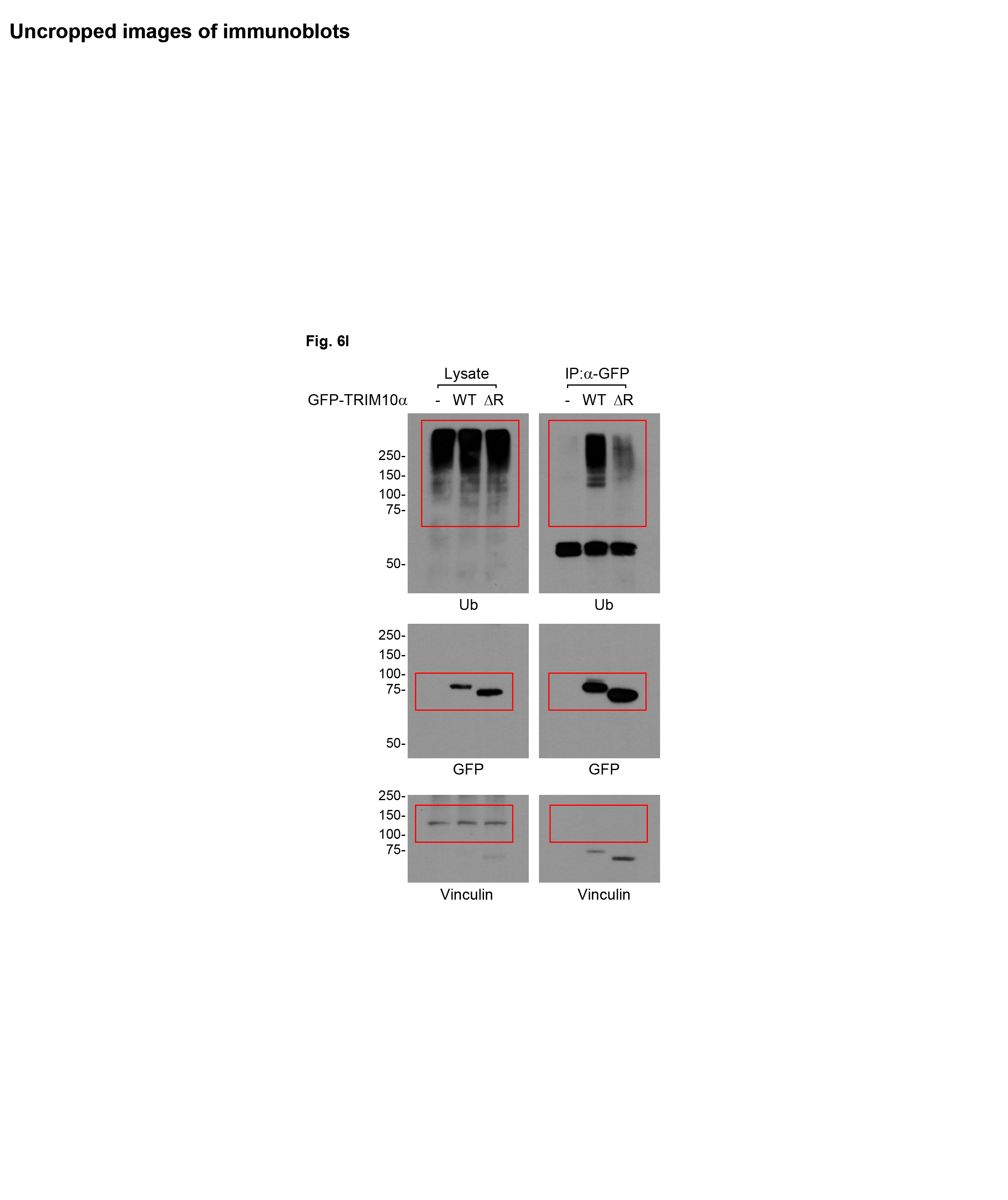

Supplement: Supplementary file 7 — Source data Fig. 6 [file 44319_2025_616_MOESM7_ESM.zip › 6I/Fig6I_Blot_data.tif]

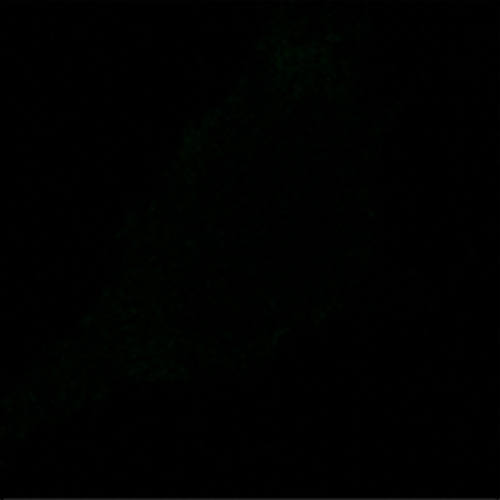

Supplement: Supplementary file 7 — Source data Fig. 6 [file 44319_2025_616_MOESM7_ESM.zip › 6J/Fig6J_Mock_GFP.tif]

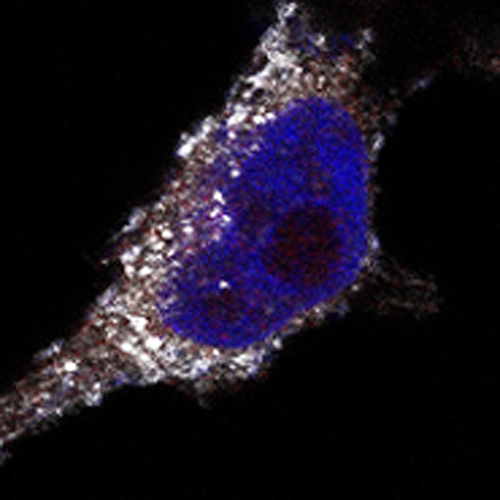

Supplement: Supplementary file 7 — Source data Fig. 6 [file 44319_2025_616_MOESM7_ESM.zip › 6J/Fig6J_Mock_Merged.tif]

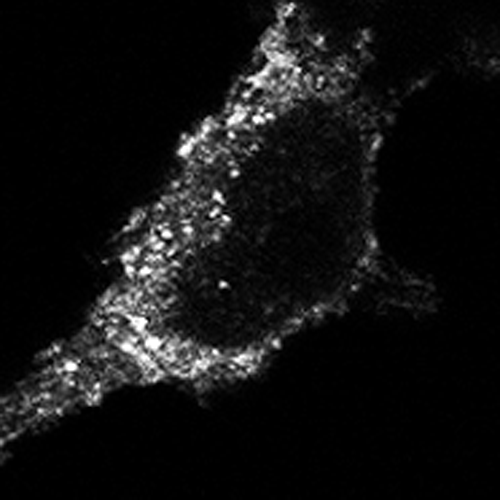

Supplement: Supplementary file 7 — Source data Fig. 6 [file 44319_2025_616_MOESM7_ESM.zip › 6J/Fig6J_Mock_p62.tif]

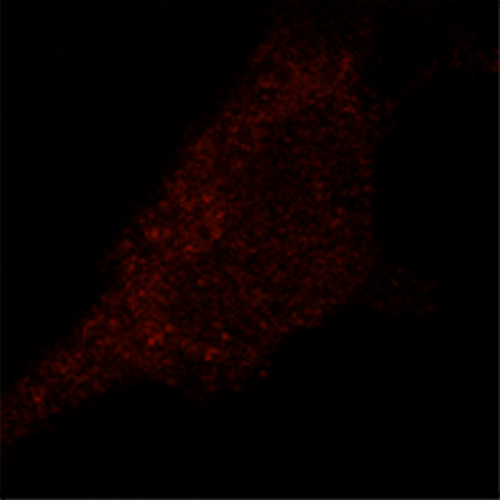

Supplement: Supplementary file 7 — Source data Fig. 6 [file 44319_2025_616_MOESM7_ESM.zip › 6J/Fig6J_Mock_Ubiquitin.tif]

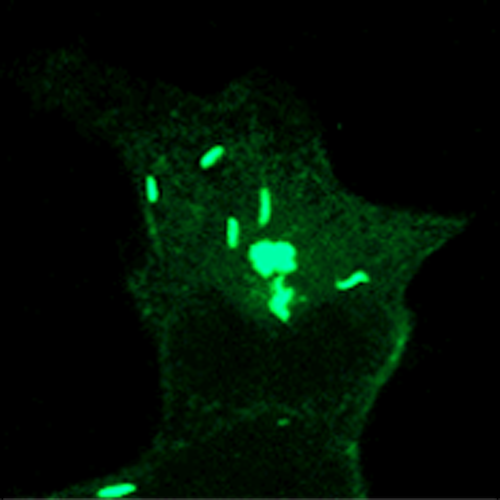

Supplement: Supplementary file 7 — Source data Fig. 6 [file 44319_2025_616_MOESM7_ESM.zip › 6J/Fig6J_RINGdel_GFP.tif]

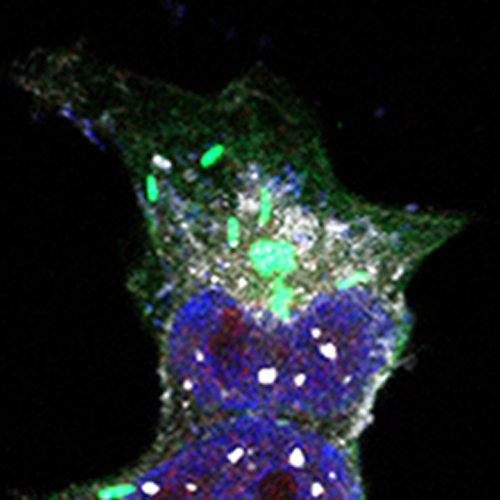

Supplement: Supplementary file 7 — Source data Fig. 6 [file 44319_2025_616_MOESM7_ESM.zip › 6J/Fig6J_RINGdel_Merged.tif]

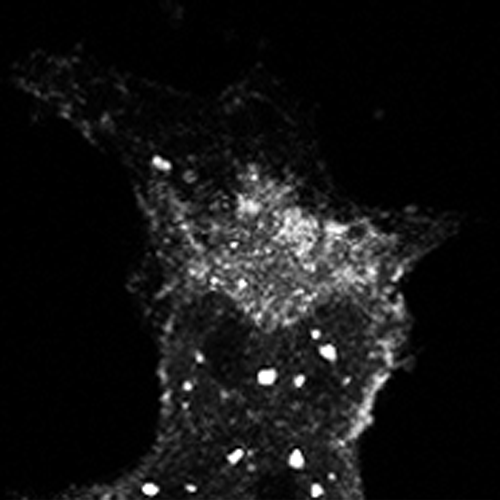

Supplement: Supplementary file 7 — Source data Fig. 6 [file 44319_2025_616_MOESM7_ESM.zip › 6J/Fig6J_RINGdel_p62.tif]

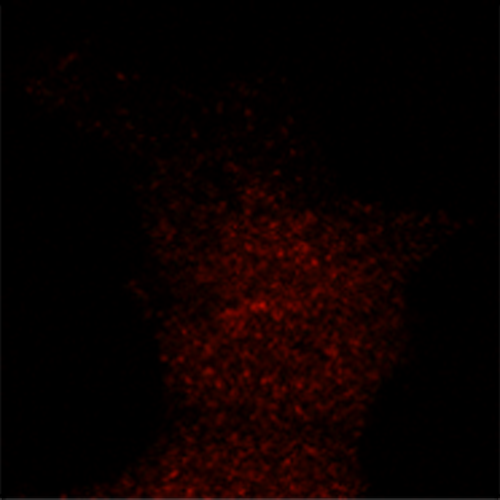

Supplement: Supplementary file 7 — Source data Fig. 6 [file 44319_2025_616_MOESM7_ESM.zip › 6J/Fig6J_RINGdel_Ubiquitin.tif]

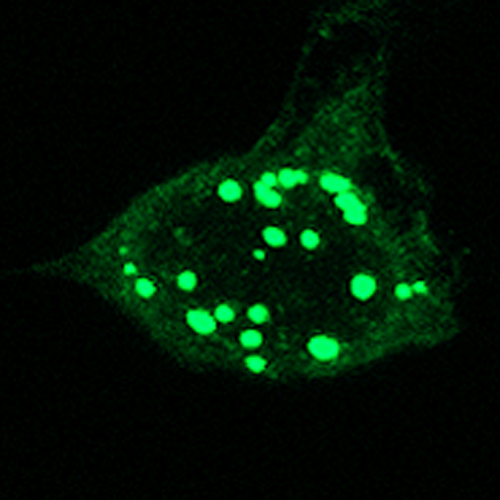

Supplement: Supplementary file 7 — Source data Fig. 6 [file 44319_2025_616_MOESM7_ESM.zip › 6J/Fig6J_WT_GFP.tif]

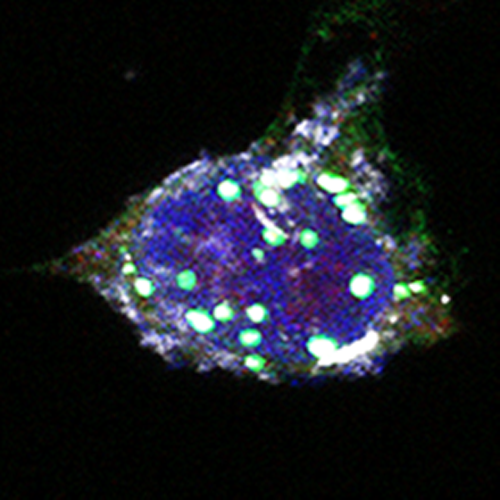

Supplement: Supplementary file 7 — Source data Fig. 6 [file 44319_2025_616_MOESM7_ESM.zip › 6J/Fig6J_WT_Merged.tif]

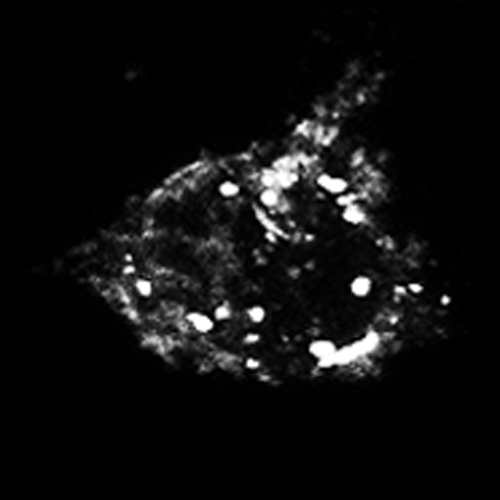

Supplement: Supplementary file 7 — Source data Fig. 6 [file 44319_2025_616_MOESM7_ESM.zip › 6J/Fig6J_WT_p62.tif]

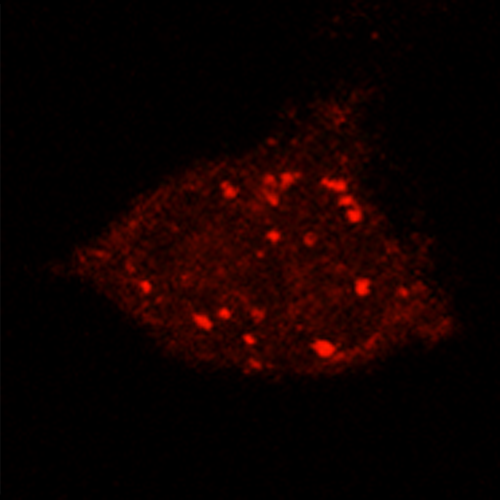

Supplement: Supplementary file 7 — Source data Fig. 6 [file 44319_2025_616_MOESM7_ESM.zip › 6J/Fig6J_WT_Ubiquitin.tif]

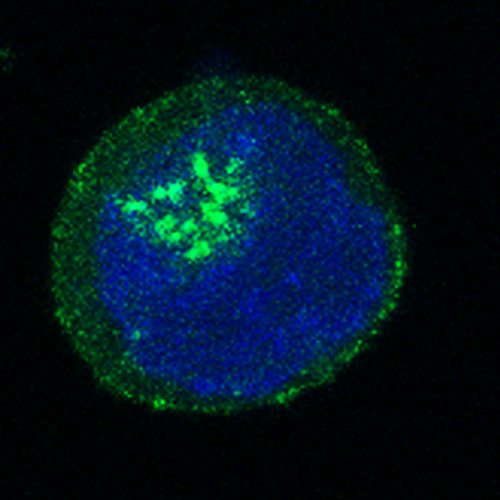

Supplement: Supplementary file 8 — Source data Fig. 7 [file 44319_2025_616_MOESM8_ESM.zip › 7B/Fig7B_GFPTRIM10alpha.tif]

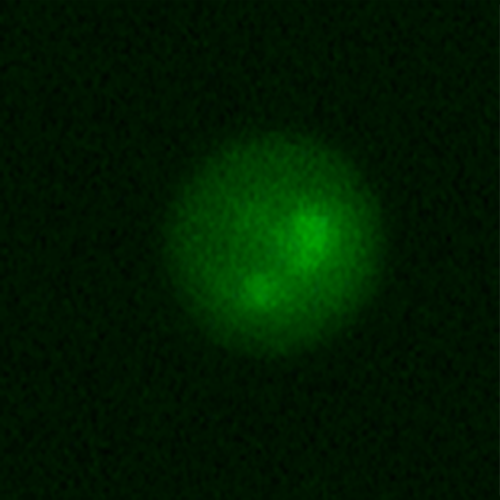

Supplement: Supplementary file 8 — Source data Fig. 7 [file 44319_2025_616_MOESM8_ESM.zip › 7B/Fig7B_GFPTRIM10beta_Highexpression.tif]

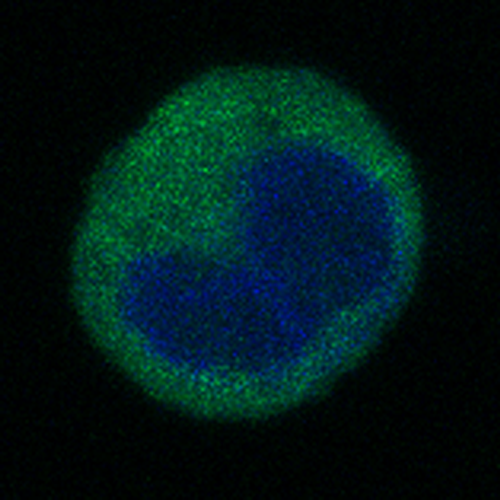

Supplement: Supplementary file 8 — Source data Fig. 7 [file 44319_2025_616_MOESM8_ESM.zip › 7B/Fig7B_GFPTRIM10beta_Lowexpression.tif]

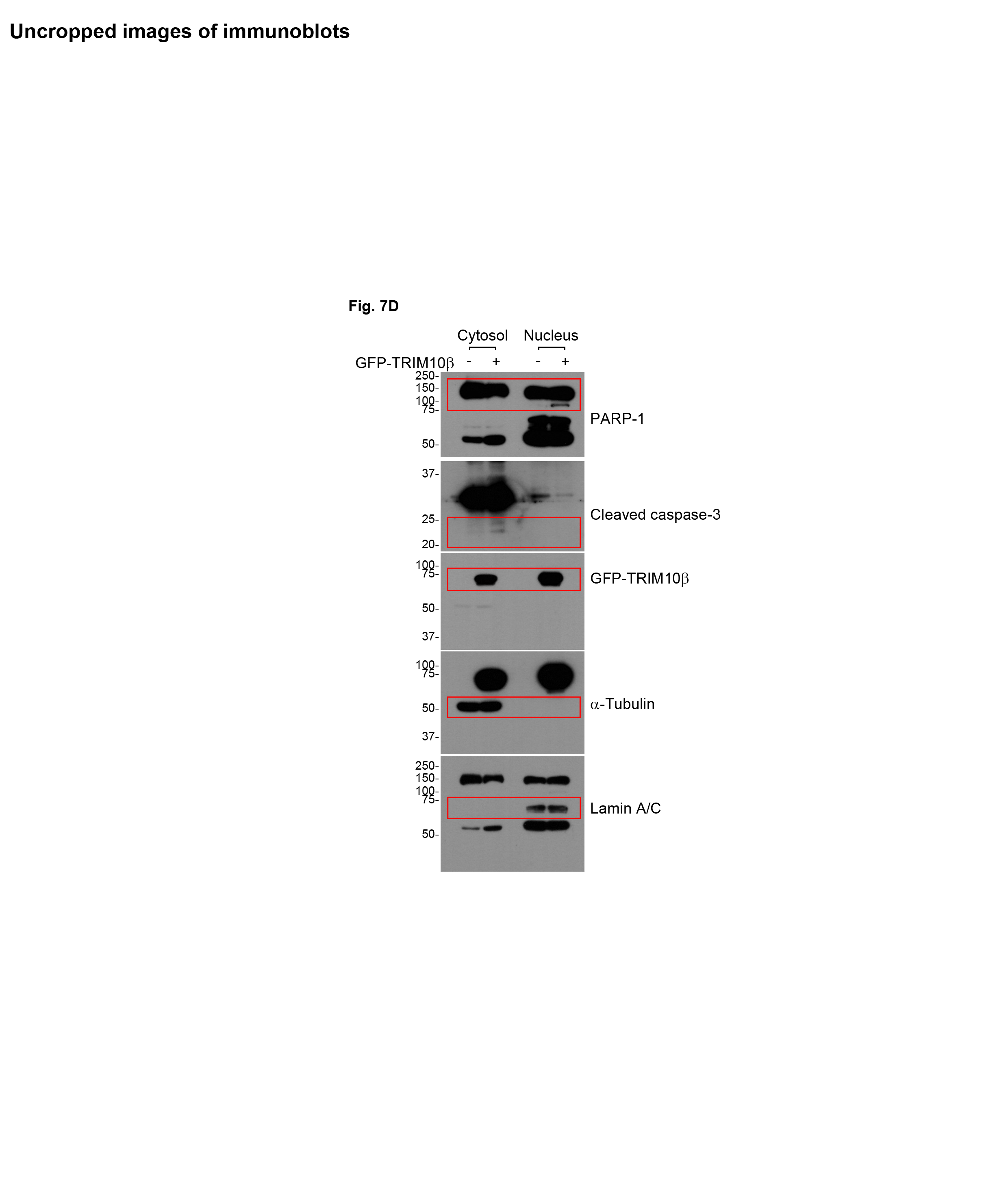

Supplement: Supplementary file 8 — Source data Fig. 7 [file 44319_2025_616_MOESM8_ESM.zip › 7D/Fig7D_Blot_data.tif]

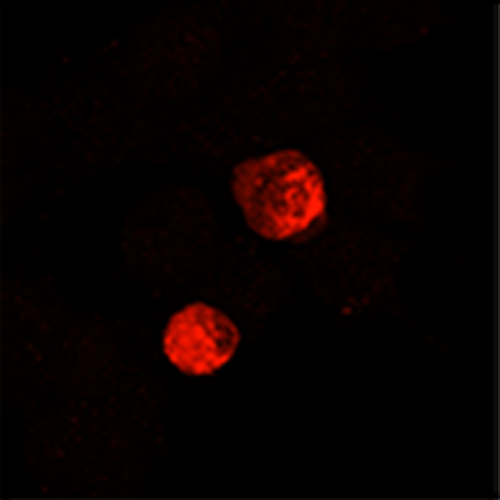

Supplement: Supplementary file 8 — Source data Fig. 7 [file 44319_2025_616_MOESM8_ESM.zip › 7E/Fig7E_GFPTRIM10beta_ActiveCasp3.tif]

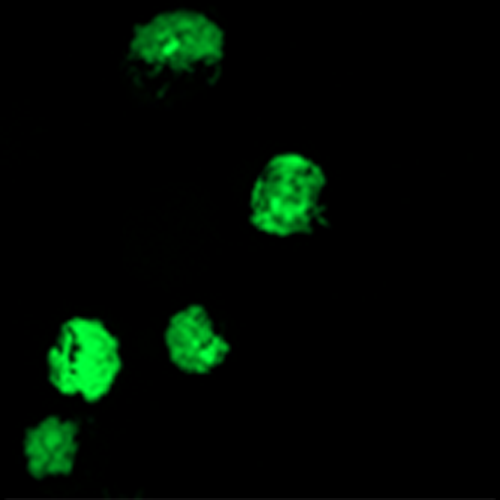

Supplement: Supplementary file 8 — Source data Fig. 7 [file 44319_2025_616_MOESM8_ESM.zip › 7E/Fig7E_GFPTRIM10beta_GFP.tif]

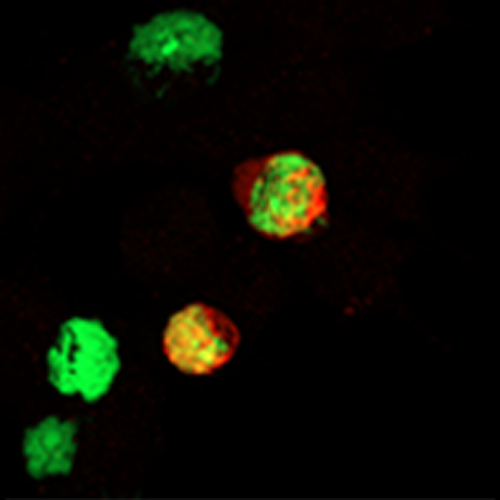

Supplement: Supplementary file 8 — Source data Fig. 7 [file 44319_2025_616_MOESM8_ESM.zip › 7E/Fig7E_GFPTRIM10beta_Merged.tif]

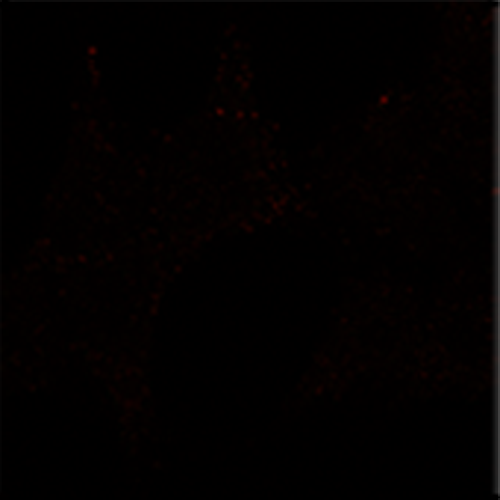

Supplement: Supplementary file 8 — Source data Fig. 7 [file 44319_2025_616_MOESM8_ESM.zip › 7E/Fig7E_Mock_ActiveCasp3.tif]

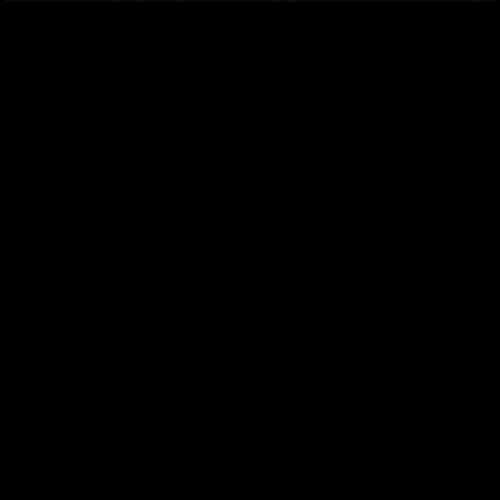

Supplement: Supplementary file 8 — Source data Fig. 7 [file 44319_2025_616_MOESM8_ESM.zip › 7E/Fig7E_Mock_GFP.tif]

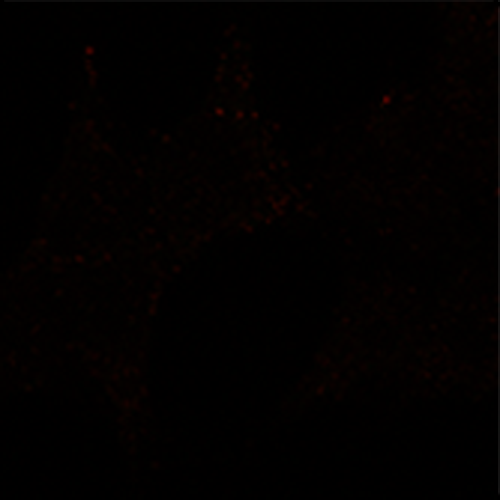

Supplement: Supplementary file 8 — Source data Fig. 7 [file 44319_2025_616_MOESM8_ESM.zip › 7E/Fig7E_Mock_Merged.tif]

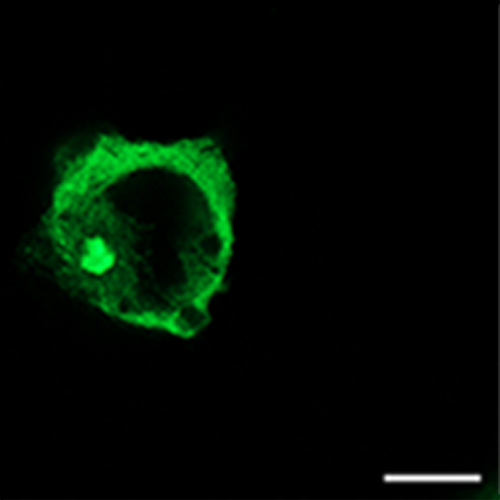

Supplement: Supplementary file 8 — Source data Fig. 7 [file 44319_2025_616_MOESM8_ESM.zip › 7F/Fig7F_LC3BP62_GFPTRIM10beta_GFP.tif]

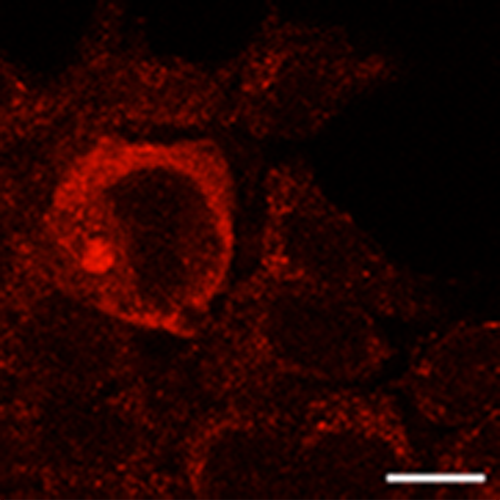

Supplement: Supplementary file 8 — Source data Fig. 7 [file 44319_2025_616_MOESM8_ESM.zip › 7F/Fig7F_LC3BP62_GFPTRIM10beta_LC3B.tif]

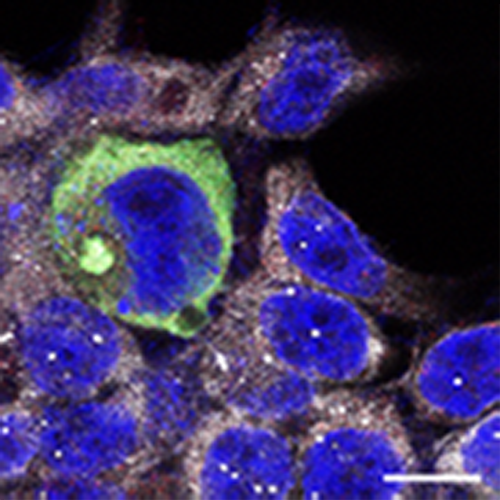

Supplement: Supplementary file 8 — Source data Fig. 7 [file 44319_2025_616_MOESM8_ESM.zip › 7F/Fig7F_LC3BP62_GFPTRIM10beta_Merged.tif]

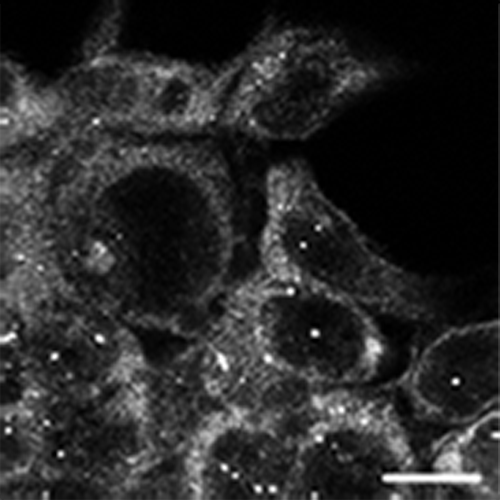

Supplement: Supplementary file 8 — Source data Fig. 7 [file 44319_2025_616_MOESM8_ESM.zip › 7F/Fig7F_LC3BP62_GFPTRIM10beta_P62.tif]

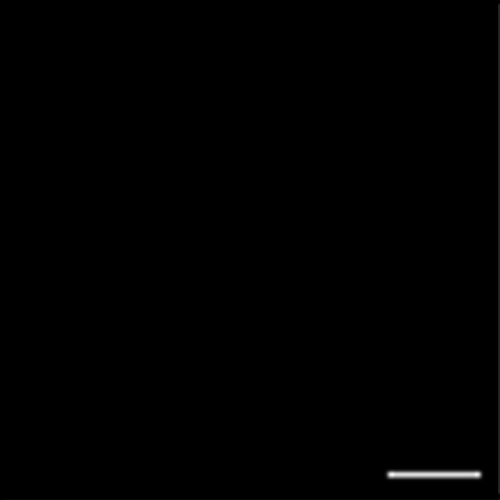

Supplement: Supplementary file 8 — Source data Fig. 7 [file 44319_2025_616_MOESM8_ESM.zip › 7F/Fig7F_LC3BP62_Mock_GFP.tif]

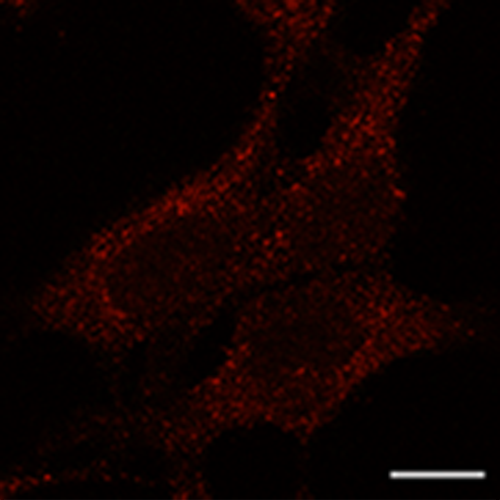

Supplement: Supplementary file 8 — Source data Fig. 7 [file 44319_2025_616_MOESM8_ESM.zip › 7F/Fig7F_LC3BP62_Mock_LC3B.tif]

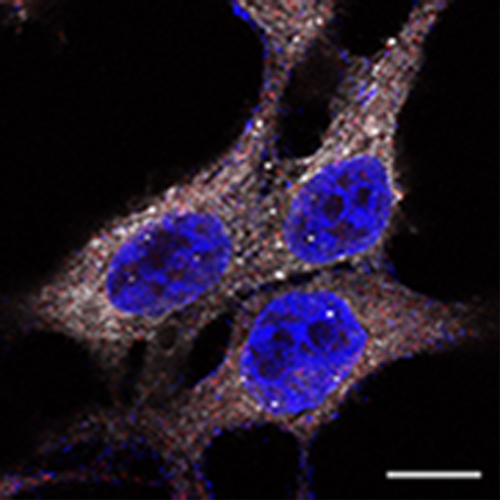

Supplement: Supplementary file 8 — Source data Fig. 7 [file 44319_2025_616_MOESM8_ESM.zip › 7F/Fig7F_LC3BP62_Mock_Merged.tif]

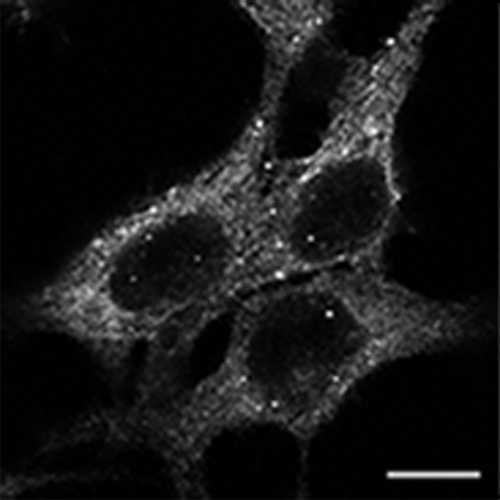

Supplement: Supplementary file 8 — Source data Fig. 7 [file 44319_2025_616_MOESM8_ESM.zip › 7F/Fig7F_LC3BP62_Mock_P62.tif]

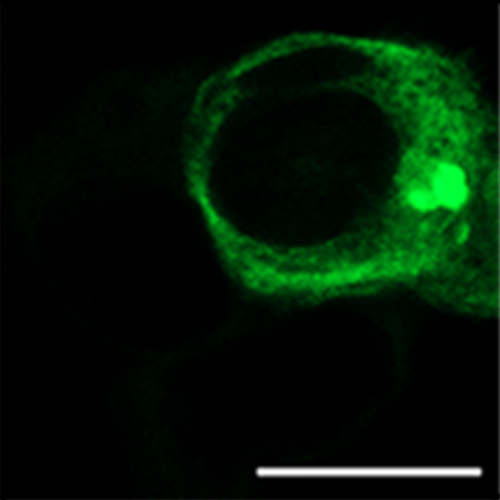

Supplement: Supplementary file 8 — Source data Fig. 7 [file 44319_2025_616_MOESM8_ESM.zip › 7F/Fig7F_Ubiquitin_GFPTRIM10beta_GFP.tif]

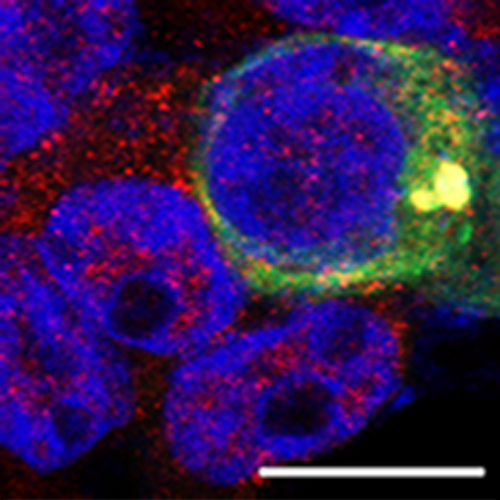

Supplement: Supplementary file 8 — Source data Fig. 7 [file 44319_2025_616_MOESM8_ESM.zip › 7F/Fig7F_Ubiquitin_GFPTRIM10beta_Merged.tif]

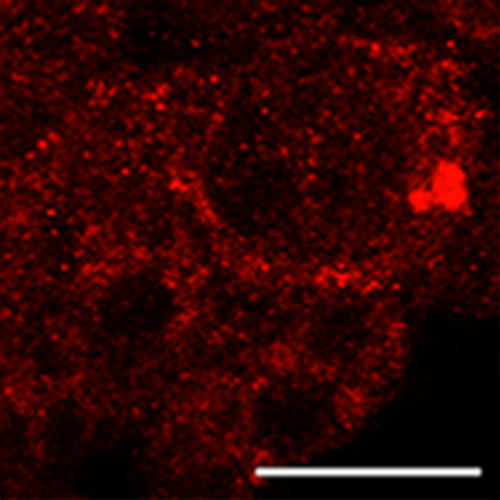

Supplement: Supplementary file 8 — Source data Fig. 7 [file 44319_2025_616_MOESM8_ESM.zip › 7F/Fig7F_Ubiquitin_GFPTRIM10beta_Ubiquitin.tif]

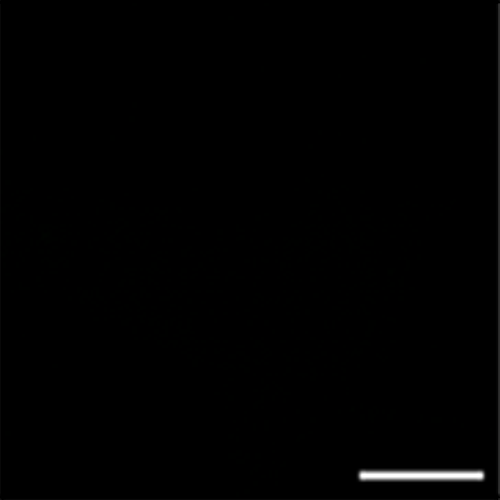

Supplement: Supplementary file 8 — Source data Fig. 7 [file 44319_2025_616_MOESM8_ESM.zip › 7F/Fig7F_Ubiquitin_Mock_GFP.tif]

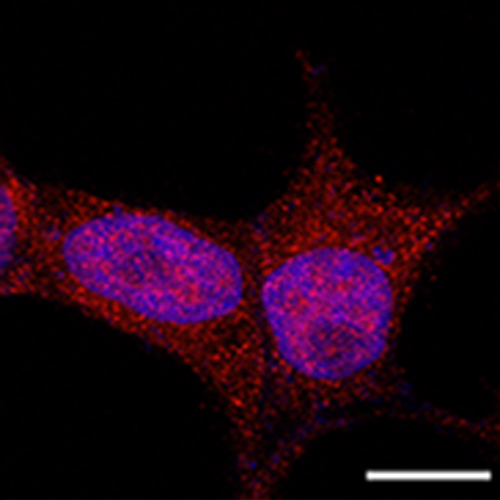

Supplement: Supplementary file 8 — Source data Fig. 7 [file 44319_2025_616_MOESM8_ESM.zip › 7F/Fig7F_Ubiquitin_Mock_Merged.tif]

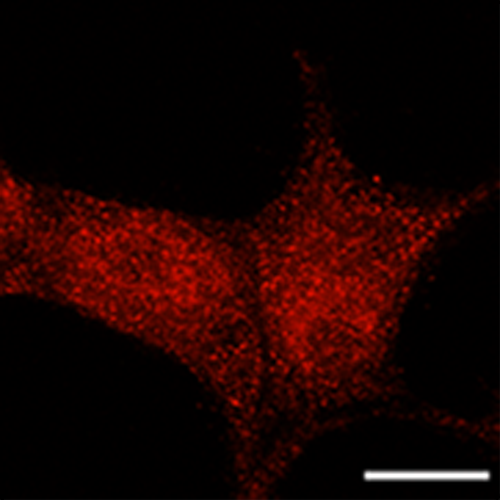

Supplement: Supplementary file 8 — Source data Fig. 7 [file 44319_2025_616_MOESM8_ESM.zip › 7F/Fig7F_Ubiquitin_Mock_Ubiquitin.tif]

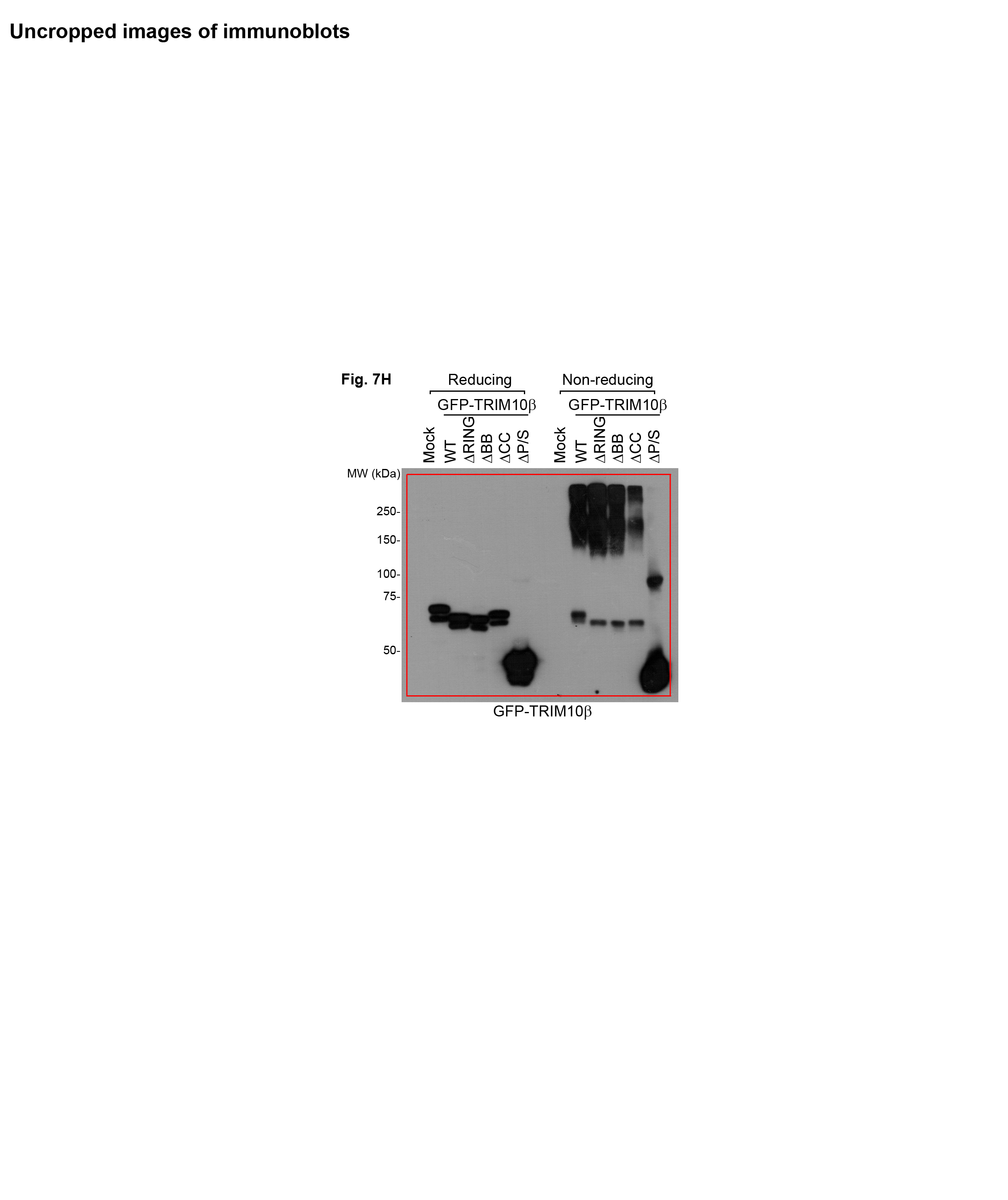

Supplement: Supplementary file 8 — Source data Fig. 7 [file 44319_2025_616_MOESM8_ESM.zip › 7H/Fig7H_Blot_data.tif]

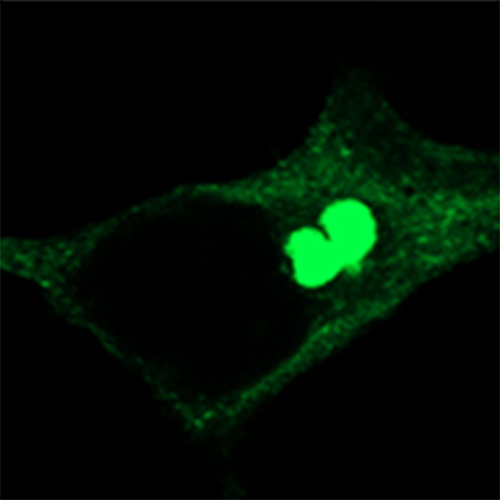

Supplement: Supplementary file 8 — Source data Fig. 7 [file 44319_2025_616_MOESM8_ESM.zip › 7I/Fig7I_GFPTRIM10beta_delBB_GFP.tif]

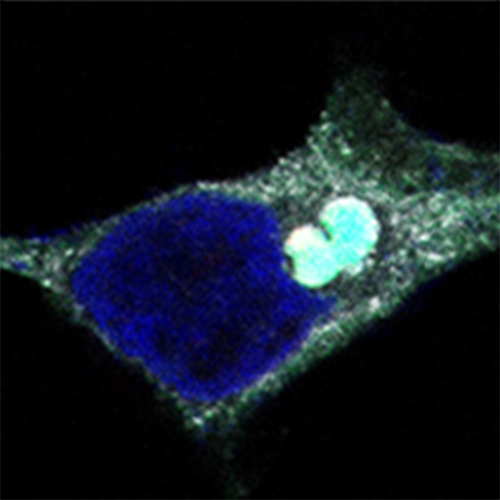

Supplement: Supplementary file 8 — Source data Fig. 7 [file 44319_2025_616_MOESM8_ESM.zip › 7I/Fig7I_GFPTRIM10beta_delBB_Merged.tif]

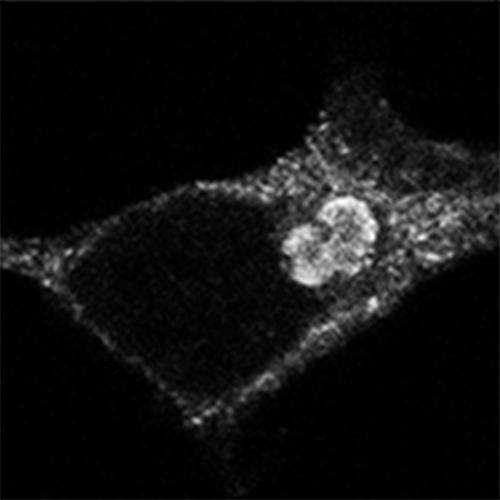

Supplement: Supplementary file 8 — Source data Fig. 7 [file 44319_2025_616_MOESM8_ESM.zip › 7I/Fig7I_GFPTRIM10beta_delBB_p62.tif]

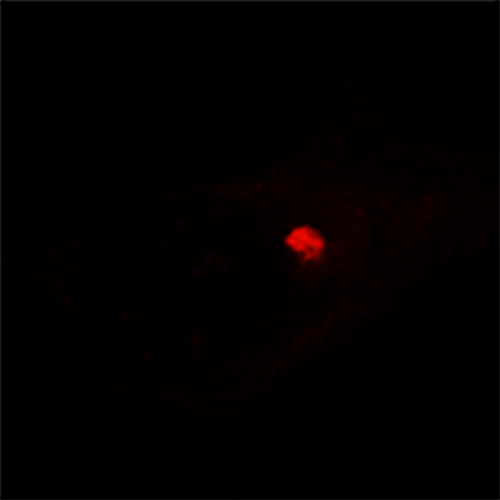

Supplement: Supplementary file 8 — Source data Fig. 7 [file 44319_2025_616_MOESM8_ESM.zip › 7I/Fig7I_GFPTRIM10beta_delBB_Pericentrin.tif]

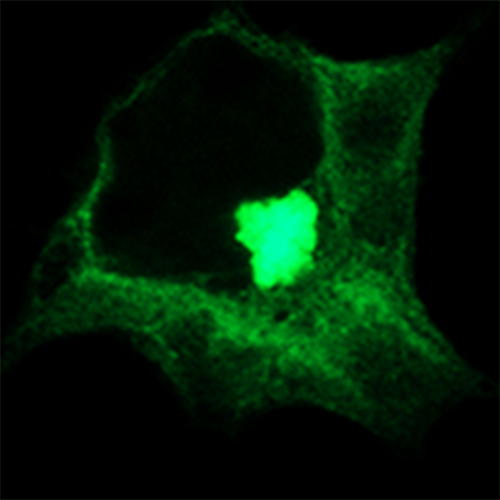

Supplement: Supplementary file 8 — Source data Fig. 7 [file 44319_2025_616_MOESM8_ESM.zip › 7I/Fig7I_GFPTRIM10beta_delCC_GFP.tif]

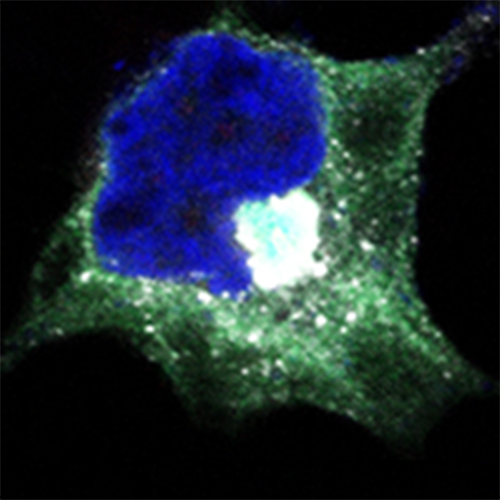

Supplement: Supplementary file 8 — Source data Fig. 7 [file 44319_2025_616_MOESM8_ESM.zip › 7I/Fig7I_GFPTRIM10beta_delCC_Merged.tif]

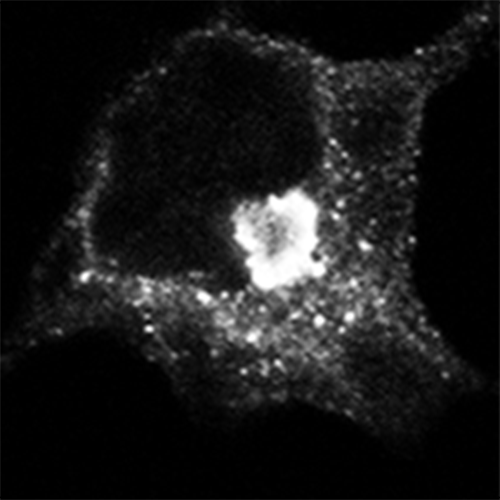

Supplement: Supplementary file 8 — Source data Fig. 7 [file 44319_2025_616_MOESM8_ESM.zip › 7I/Fig7I_GFPTRIM10beta_delCC_p62.tif]

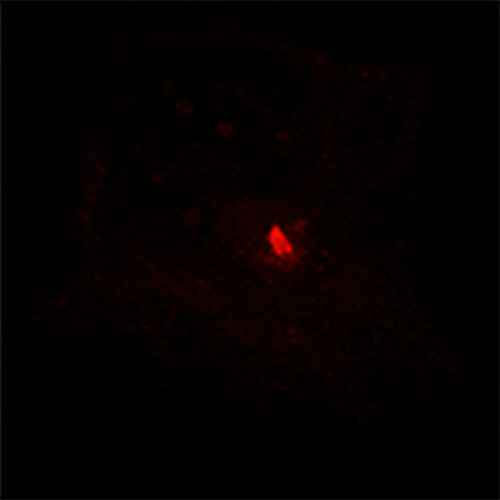

Supplement: Supplementary file 8 — Source data Fig. 7 [file 44319_2025_616_MOESM8_ESM.zip › 7I/Fig7I_GFPTRIM10beta_delCC_Pericentrin.tif]

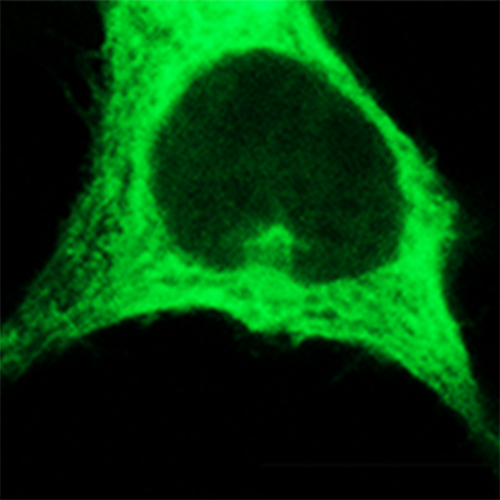

Supplement: Supplementary file 8 — Source data Fig. 7 [file 44319_2025_616_MOESM8_ESM.zip › 7I/Fig7I_GFPTRIM10beta_delP-S_GFP.tif]

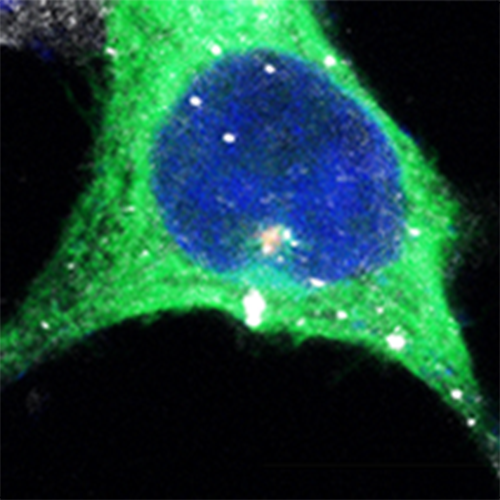

Supplement: Supplementary file 8 — Source data Fig. 7 [file 44319_2025_616_MOESM8_ESM.zip › 7I/Fig7I_GFPTRIM10beta_delP-S_Merged.tif]

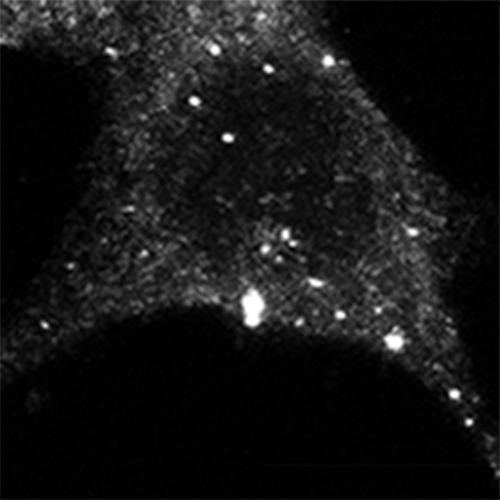

Supplement: Supplementary file 8 — Source data Fig. 7 [file 44319_2025_616_MOESM8_ESM.zip › 7I/Fig7I_GFPTRIM10beta_delP-S_p62.tif]

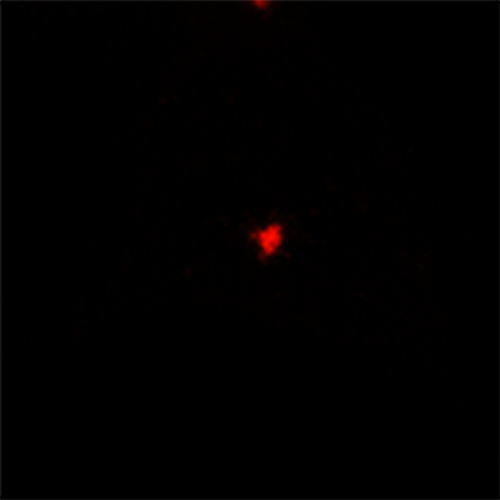

Supplement: Supplementary file 8 — Source data Fig. 7 [file 44319_2025_616_MOESM8_ESM.zip › 7I/Fig7I_GFPTRIM10beta_delP-S_Pericentrin.tif]

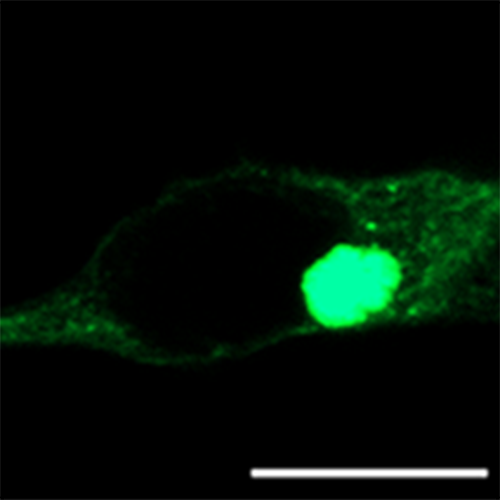

Supplement: Supplementary file 8 — Source data Fig. 7 [file 44319_2025_616_MOESM8_ESM.zip › 7I/Fig7I_GFPTRIM10beta_delRING_GFP.tif]

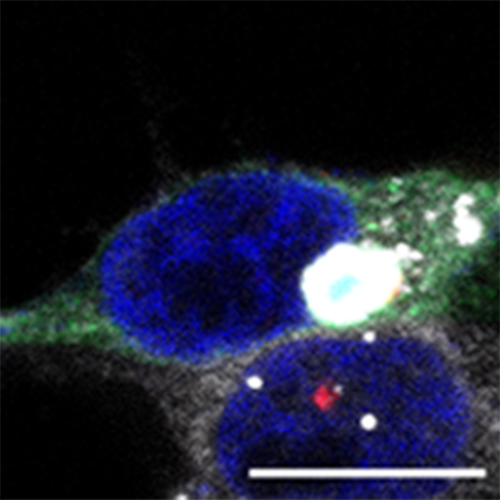

Supplement: Supplementary file 8 — Source data Fig. 7 [file 44319_2025_616_MOESM8_ESM.zip › 7I/Fig7I_GFPTRIM10beta_delRING_Merged.tif]

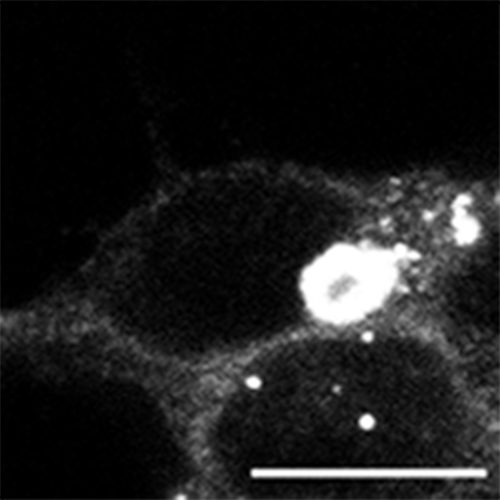

Supplement: Supplementary file 8 — Source data Fig. 7 [file 44319_2025_616_MOESM8_ESM.zip › 7I/Fig7I_GFPTRIM10beta_delRING_p62.tif]

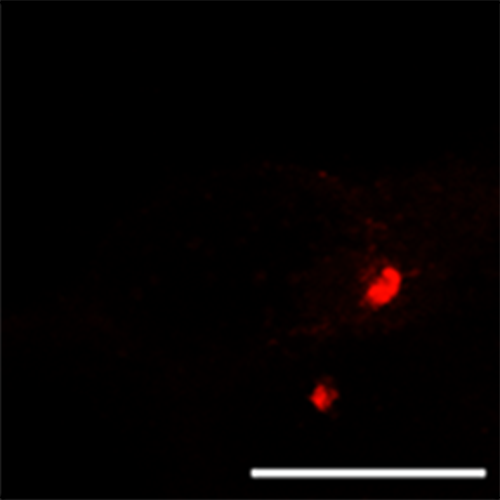

Supplement: Supplementary file 8 — Source data Fig. 7 [file 44319_2025_616_MOESM8_ESM.zip › 7I/Fig7I_GFPTRIM10beta_delRING_Pericentrin.tif]

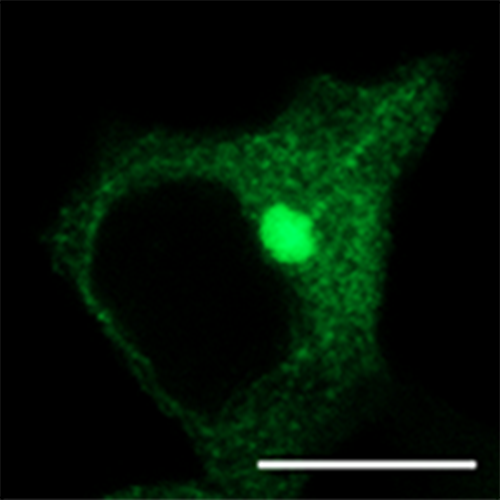

Supplement: Supplementary file 8 — Source data Fig. 7 [file 44319_2025_616_MOESM8_ESM.zip › 7I/Fig7I_GFPTRIM10beta_WT_GFP.tif]

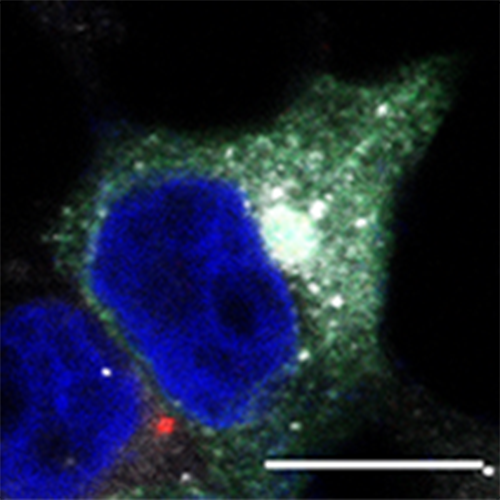

Supplement: Supplementary file 8 — Source data Fig. 7 [file 44319_2025_616_MOESM8_ESM.zip › 7I/Fig7I_GFPTRIM10beta_WT_Merged.tif]

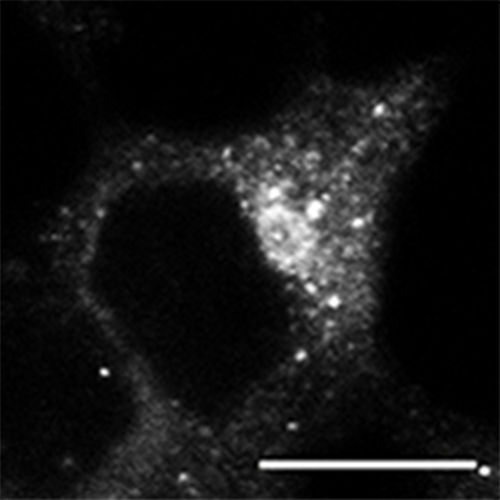

Supplement: Supplementary file 8 — Source data Fig. 7 [file 44319_2025_616_MOESM8_ESM.zip › 7I/Fig7I_GFPTRIM10beta_WT_p62.tif]

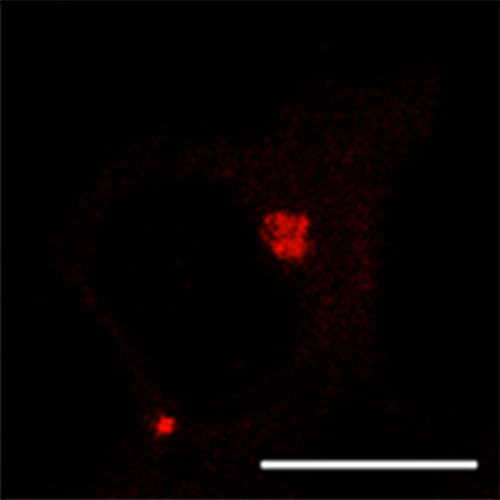

Supplement: Supplementary file 8 — Source data Fig. 7 [file 44319_2025_616_MOESM8_ESM.zip › 7I/Fig7I_GFPTRIM10beta_WT_Pericentrin.tif]

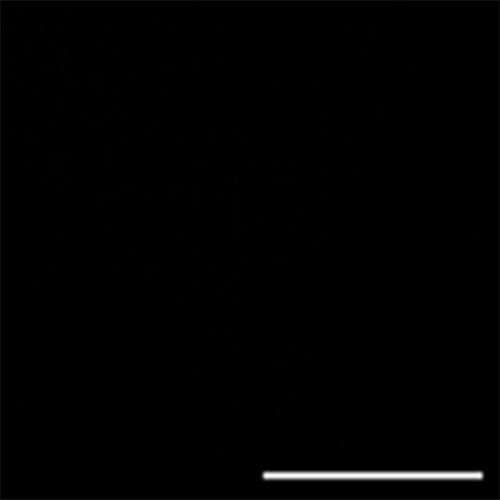

Supplement: Supplementary file 8 — Source data Fig. 7 [file 44319_2025_616_MOESM8_ESM.zip › 7I/Fig7I_Mock_GFP.tif]

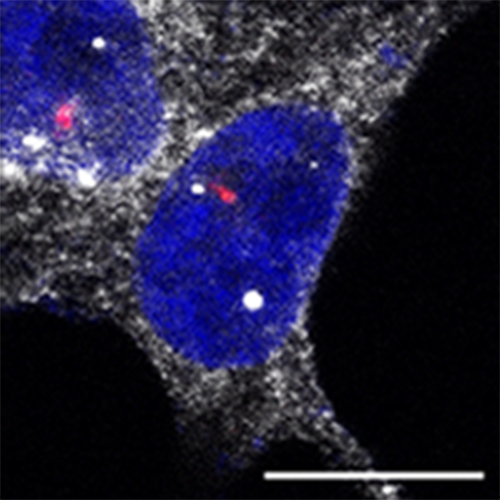

Supplement: Supplementary file 8 — Source data Fig. 7 [file 44319_2025_616_MOESM8_ESM.zip › 7I/Fig7I_Mock_Merged.tif]

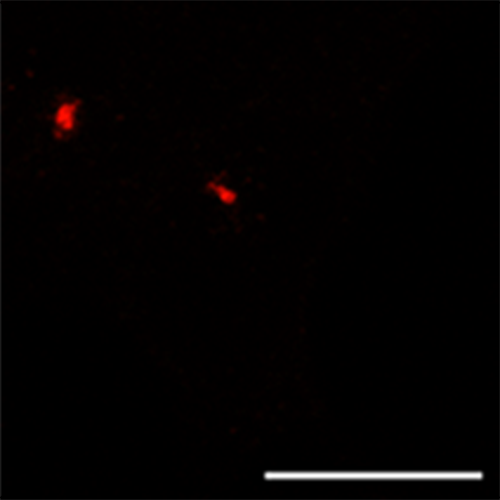

Supplement: Supplementary file 8 — Source data Fig. 7 [file 44319_2025_616_MOESM8_ESM.zip › 7I/Fig7I_Mock_Pericentrin.tif]

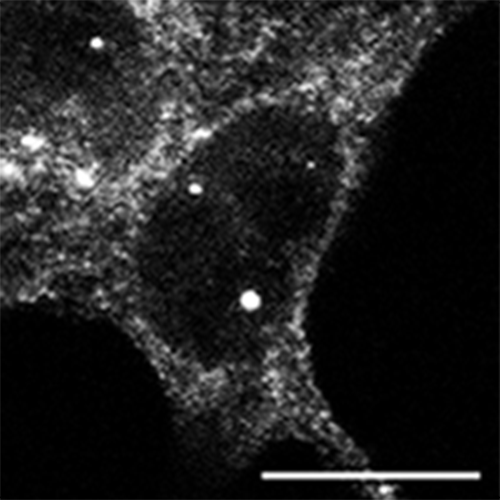

Supplement: Supplementary file 8 — Source data Fig. 7 [file 44319_2025_616_MOESM8_ESM.zip › 7I/FigI_Mock_p62.tif]

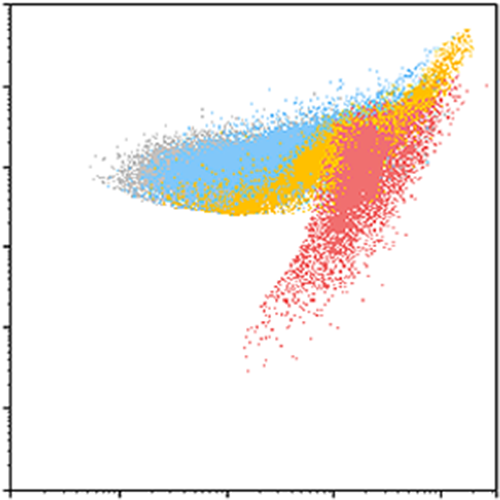

Supplement: Supplementary file 9 — EV Figures Source Data [file 44319_2025_616_MOESM9_ESM.zip › Figure EV1/EV1E/FigEV1E_HUDEP-2_hCD36_hCD235a.tif]

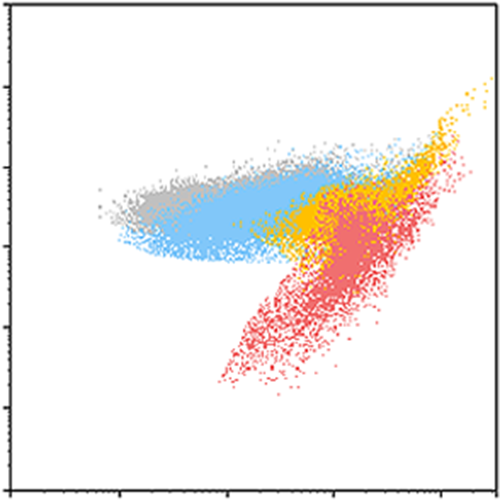

Supplement: Supplementary file 9 — EV Figures Source Data [file 44319_2025_616_MOESM9_ESM.zip › Figure EV1/EV1E/FigEV1E_HUDEP-2_hCD49d_hCD235a.tif]

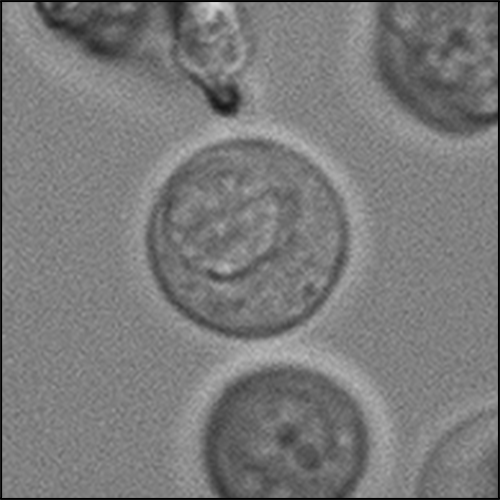

Supplement: Supplementary file 9 — EV Figures Source Data [file 44319_2025_616_MOESM9_ESM.zip › Figure EV1/EV1F/FigEV1F_DifferentiatedHUDEP-2_left.tif]

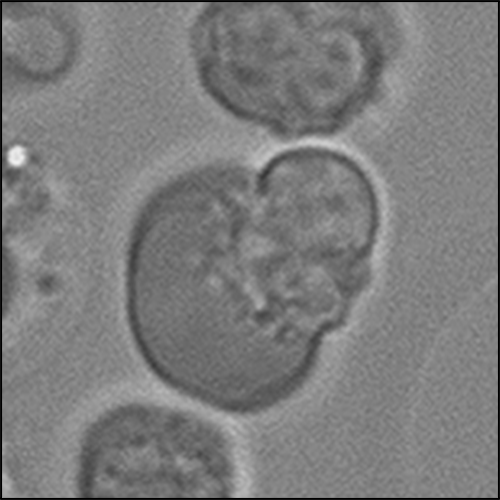

Supplement: Supplementary file 9 — EV Figures Source Data [file 44319_2025_616_MOESM9_ESM.zip › Figure EV1/EV1F/FigEV1F_DifferentiatedHUDEP-2_right.tif]

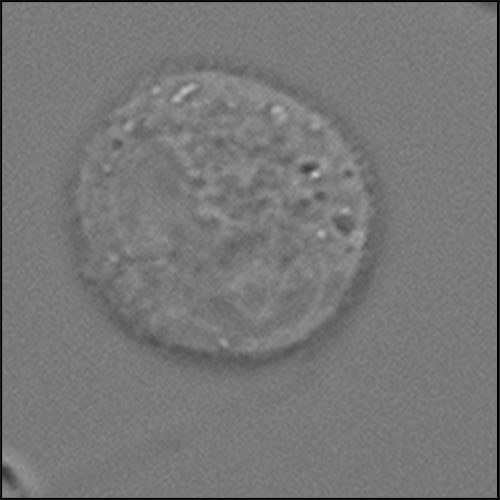

Supplement: Supplementary file 9 — EV Figures Source Data [file 44319_2025_616_MOESM9_ESM.zip › Figure EV1/EV1F/FigEV1F_HUDEP-2.tif]

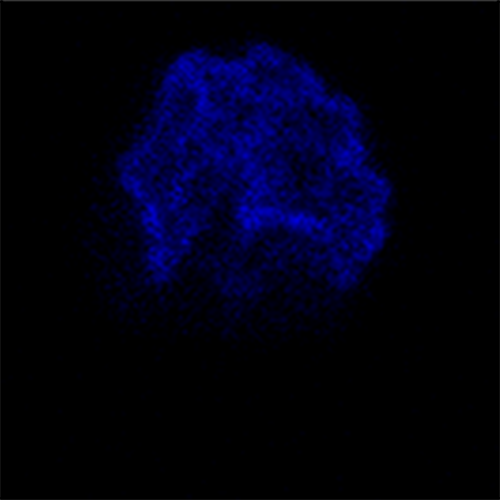

Supplement: Supplementary file 9 — EV Figures Source Data [file 44319_2025_616_MOESM9_ESM.zip › Figure EV2/EV2A/FigEV2A_D4_DAPI.tif]

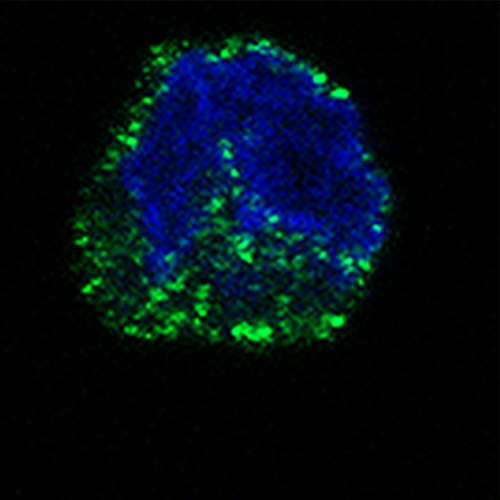

Supplement: Supplementary file 9 — EV Figures Source Data [file 44319_2025_616_MOESM9_ESM.zip › Figure EV2/EV2A/FigEV2A_D4_Merged.tif]

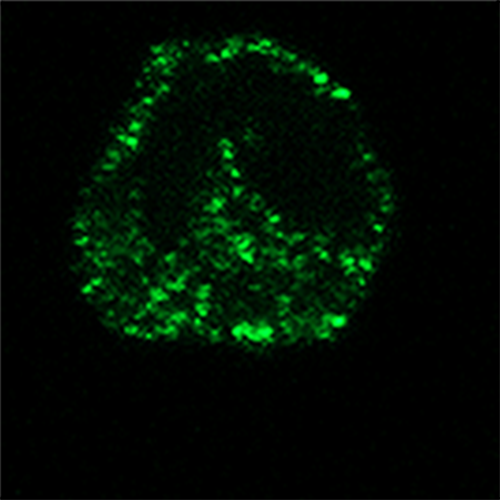

Supplement: Supplementary file 9 — EV Figures Source Data [file 44319_2025_616_MOESM9_ESM.zip › Figure EV2/EV2A/FigEV2A_D4_TRIM10alpha.tif]

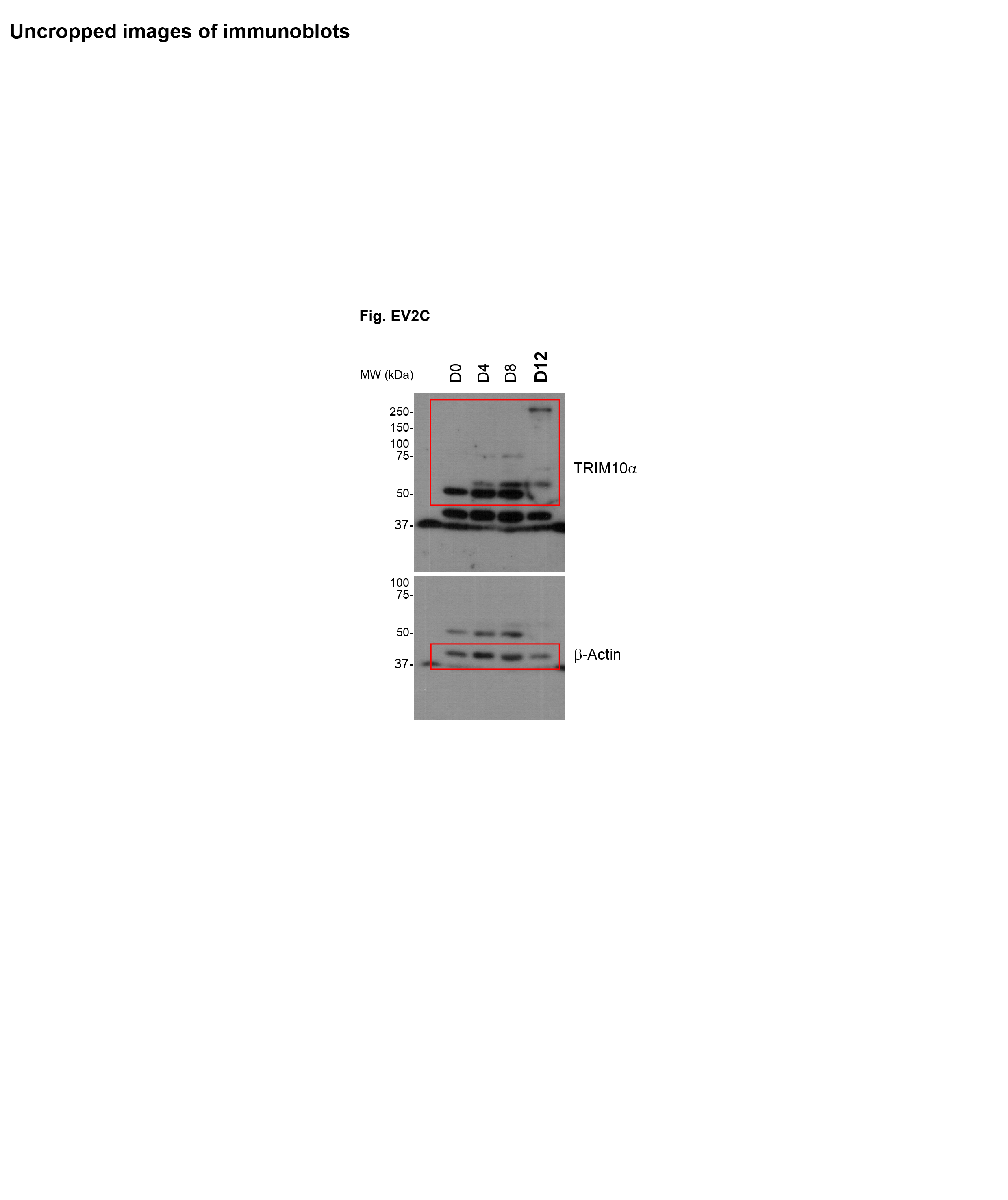

Supplement: Supplementary file 9 — EV Figures Source Data [file 44319_2025_616_MOESM9_ESM.zip › Figure EV2/EV2C/FigEV2C_Blot_data.tif]

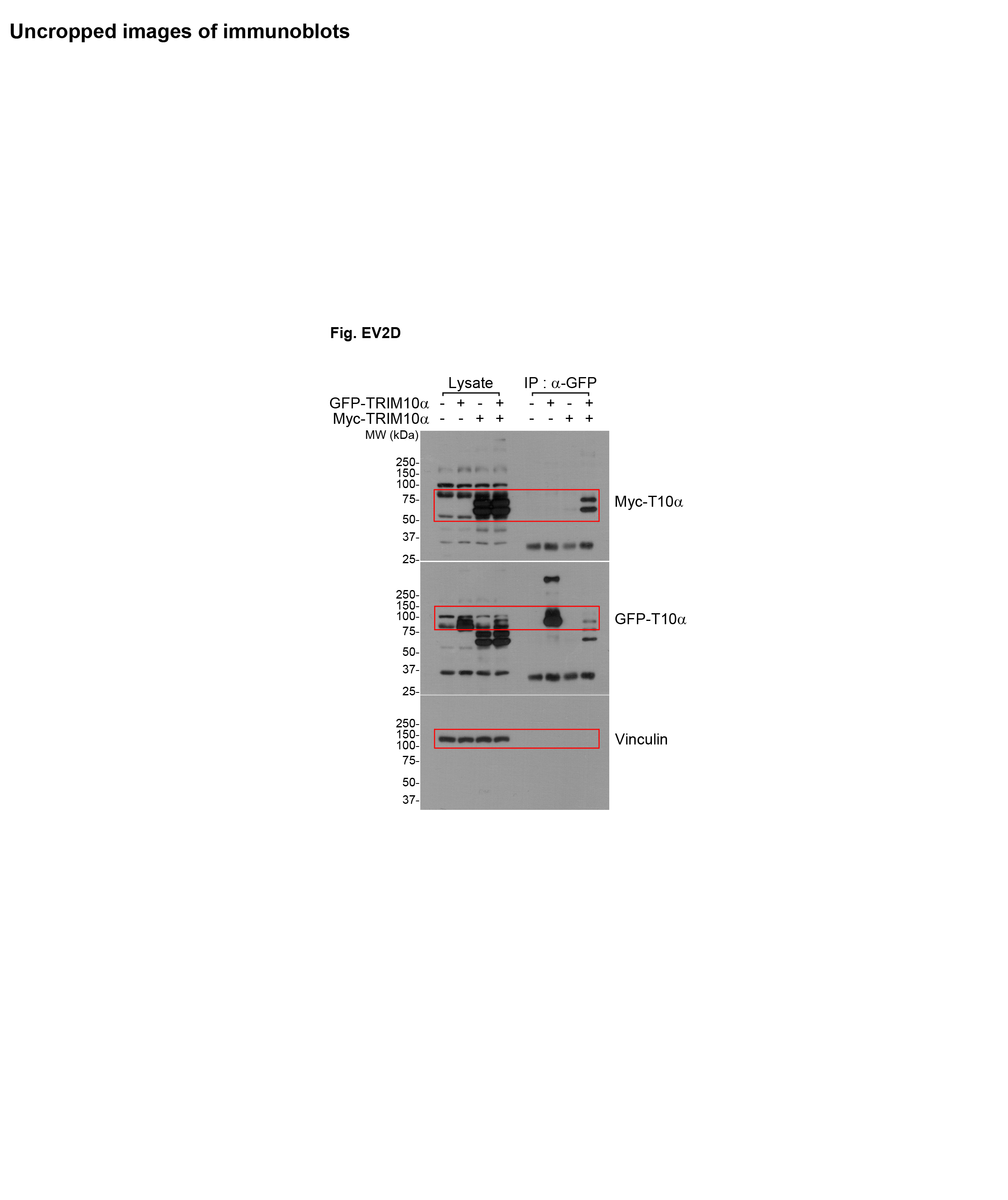

Supplement: Supplementary file 9 — EV Figures Source Data [file 44319_2025_616_MOESM9_ESM.zip › Figure EV2/EV2D/FigEV2D_Blot_data.tif]

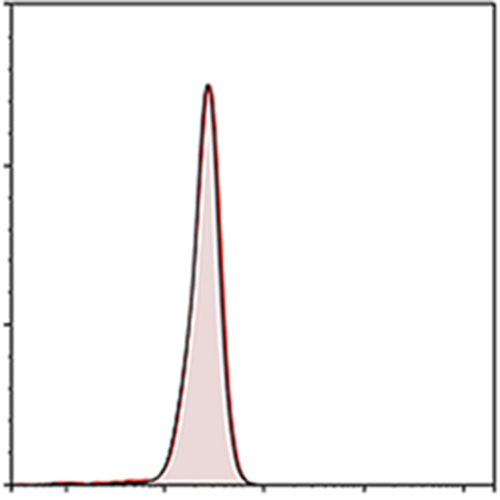

Supplement: Supplementary file 9 — EV Figures Source Data [file 44319_2025_616_MOESM9_ESM.zip › Figure EV2/EV2E/FigEV2E_Mock.tif]

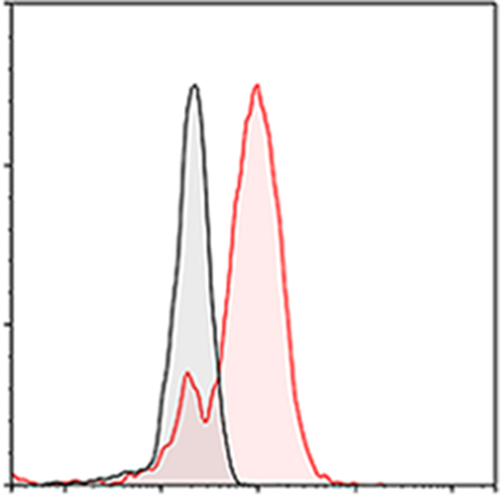

Supplement: Supplementary file 9 — EV Figures Source Data [file 44319_2025_616_MOESM9_ESM.zip › Figure EV2/EV2E/FigEV2E_WT.tif]

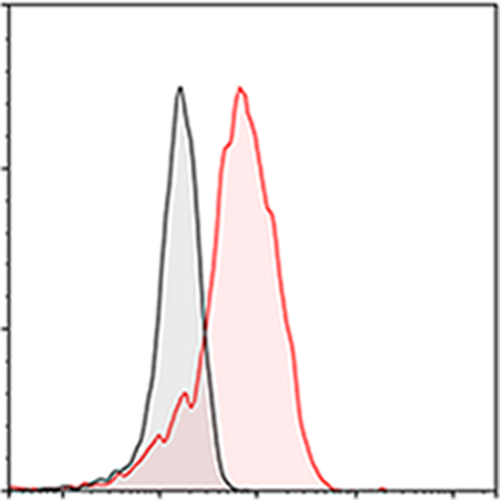

Supplement: Supplementary file 9 — EV Figures Source Data [file 44319_2025_616_MOESM9_ESM.zip › Figure EV2/EV2E/FigEV2E_delBB.tif]

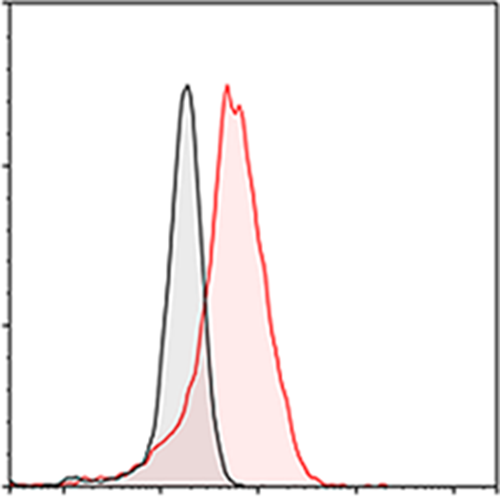

Supplement: Supplementary file 9 — EV Figures Source Data [file 44319_2025_616_MOESM9_ESM.zip › Figure EV2/EV2E/FigEV2E_delCC.tif]

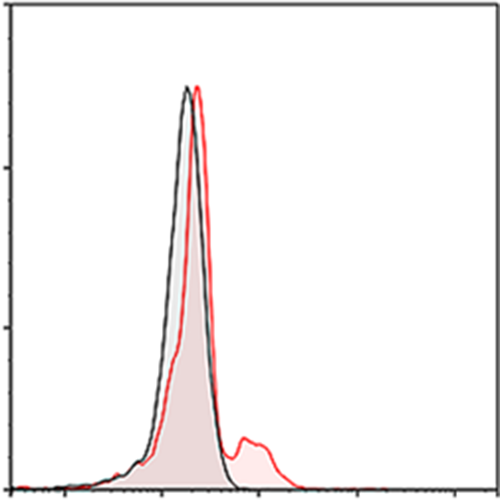

Supplement: Supplementary file 9 — EV Figures Source Data [file 44319_2025_616_MOESM9_ESM.zip › Figure EV2/EV2E/FigEV2E_delPRYSPRY.tif]

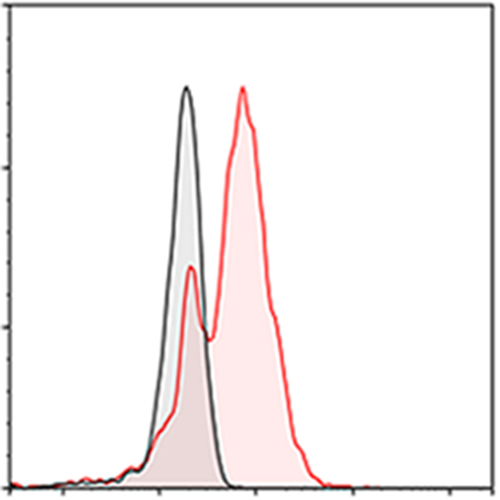

Supplement: Supplementary file 9 — EV Figures Source Data [file 44319_2025_616_MOESM9_ESM.zip › Figure EV2/EV2E/FigEV2E_delRING.tif]

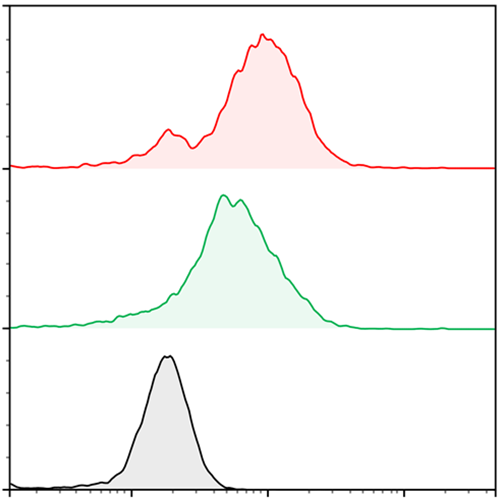

Supplement: Supplementary file 9 — EV Figures Source Data [file 44319_2025_616_MOESM9_ESM.zip › Figure EV2/EV2G/FigEV2G_Surface_MycTRIM10alpha.tif]

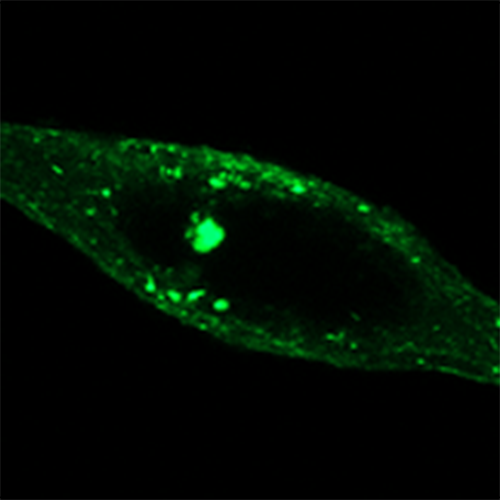

Supplement: Supplementary file 9 — EV Figures Source Data [file 44319_2025_616_MOESM9_ESM.zip › Figure EV2/EV2H/FigEV2H_TRIM10alpha_WT_GFP.tif]

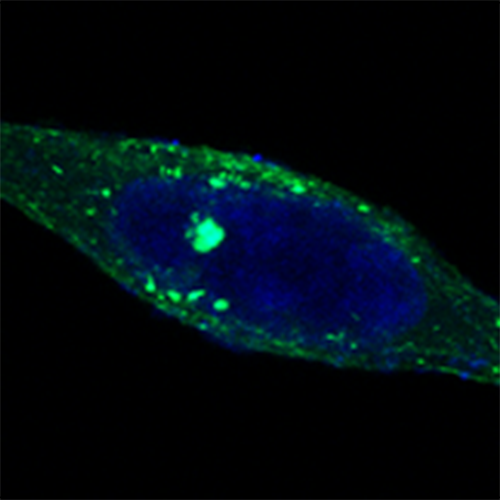

Supplement: Supplementary file 9 — EV Figures Source Data [file 44319_2025_616_MOESM9_ESM.zip › Figure EV2/EV2H/FigEV2H_TRIM10alpha_WT_Merged.tif]

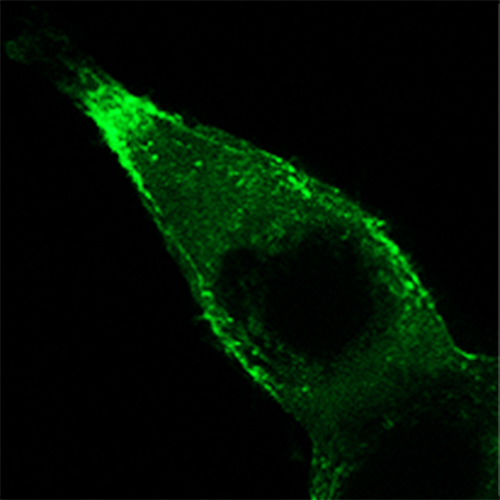

Supplement: Supplementary file 9 — EV Figures Source Data [file 44319_2025_616_MOESM9_ESM.zip › Figure EV2/EV2H/FigEV2H_TRIM10alpha_delBB_GFP.tif]

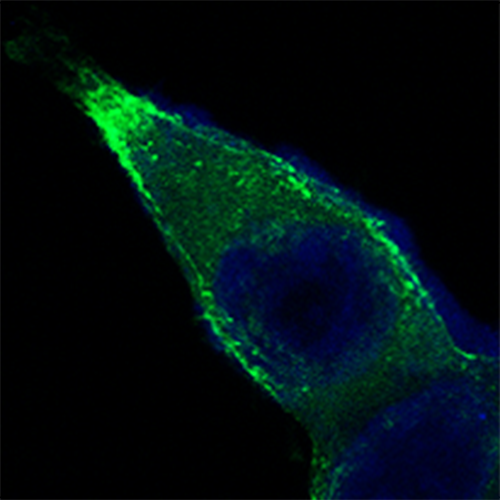

Supplement: Supplementary file 9 — EV Figures Source Data [file 44319_2025_616_MOESM9_ESM.zip › Figure EV2/EV2H/FigEV2H_TRIM10alpha_delBB_Merged.tif]

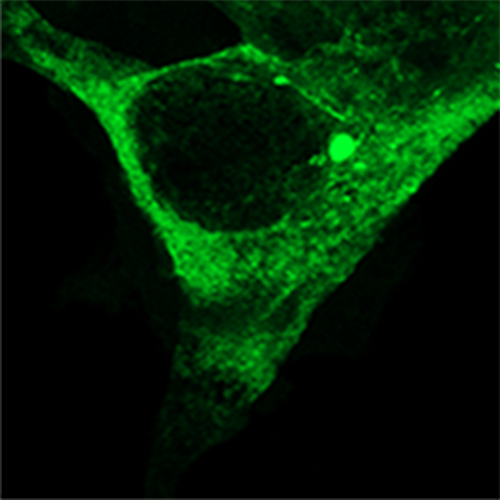

Supplement: Supplementary file 9 — EV Figures Source Data [file 44319_2025_616_MOESM9_ESM.zip › Figure EV2/EV2H/FigEV2H_TRIM10alpha_delCC_GFP.tif]

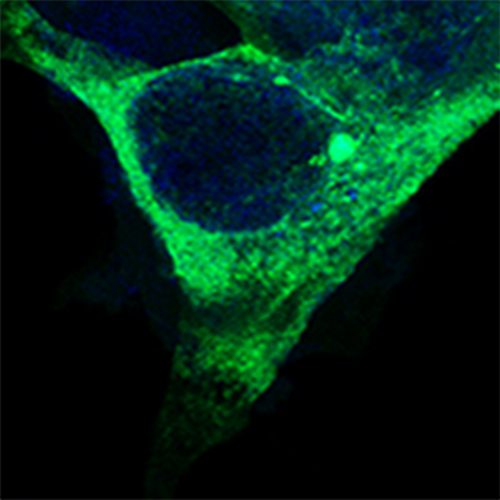

Supplement: Supplementary file 9 — EV Figures Source Data [file 44319_2025_616_MOESM9_ESM.zip › Figure EV2/EV2H/FigEV2H_TRIM10alpha_delCC_Merged.tif]
