# Supplementary figures and images for: Stage-specific TRIM10 expression regulates erythroid maturation (part 3 of 3)
Source: EMBO Rep. 2025 Oct 30;26(23):5982–6014. doi: 10.1038/s44319-025-00616-0 (PMC12678476; doi:10.1038/s44319-025-00616-0)

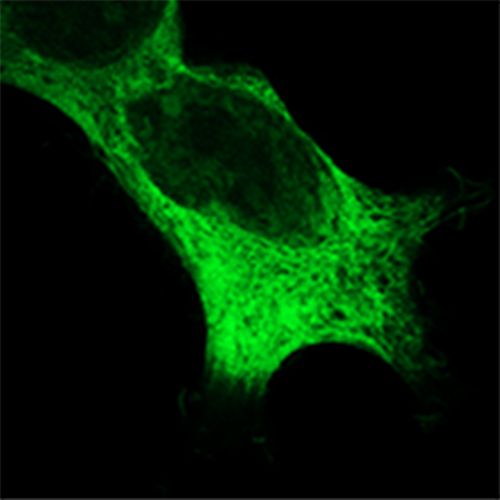

Supplement: Supplementary file 9 — EV Figures Source Data [file 44319_2025_616_MOESM9_ESM.zip › Figure EV2/EV2H/FigEV2H_TRIM10alpha_delPRYSPRY_GFP.tif]

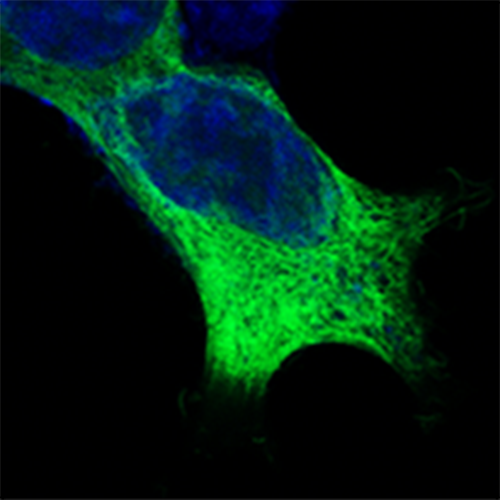

Supplement: Supplementary file 9 — EV Figures Source Data [file 44319_2025_616_MOESM9_ESM.zip › Figure EV2/EV2H/FigEV2H_TRIM10alpha_delPRYSPRY_Merged.tif]

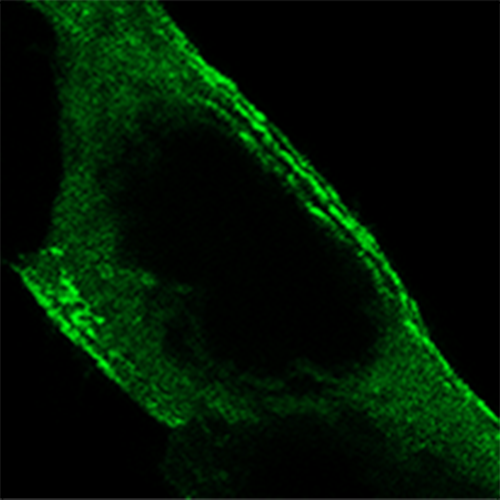

Supplement: Supplementary file 9 — EV Figures Source Data [file 44319_2025_616_MOESM9_ESM.zip › Figure EV2/EV2H/FigEV2H_TRIM10alpha_delRING_GFP.tif]

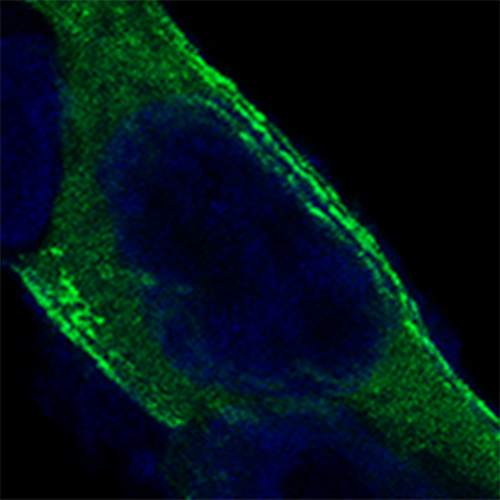

Supplement: Supplementary file 9 — EV Figures Source Data [file 44319_2025_616_MOESM9_ESM.zip › Figure EV2/EV2H/FigEV2H_TRIM10alpha_delRING_Merged.tif]

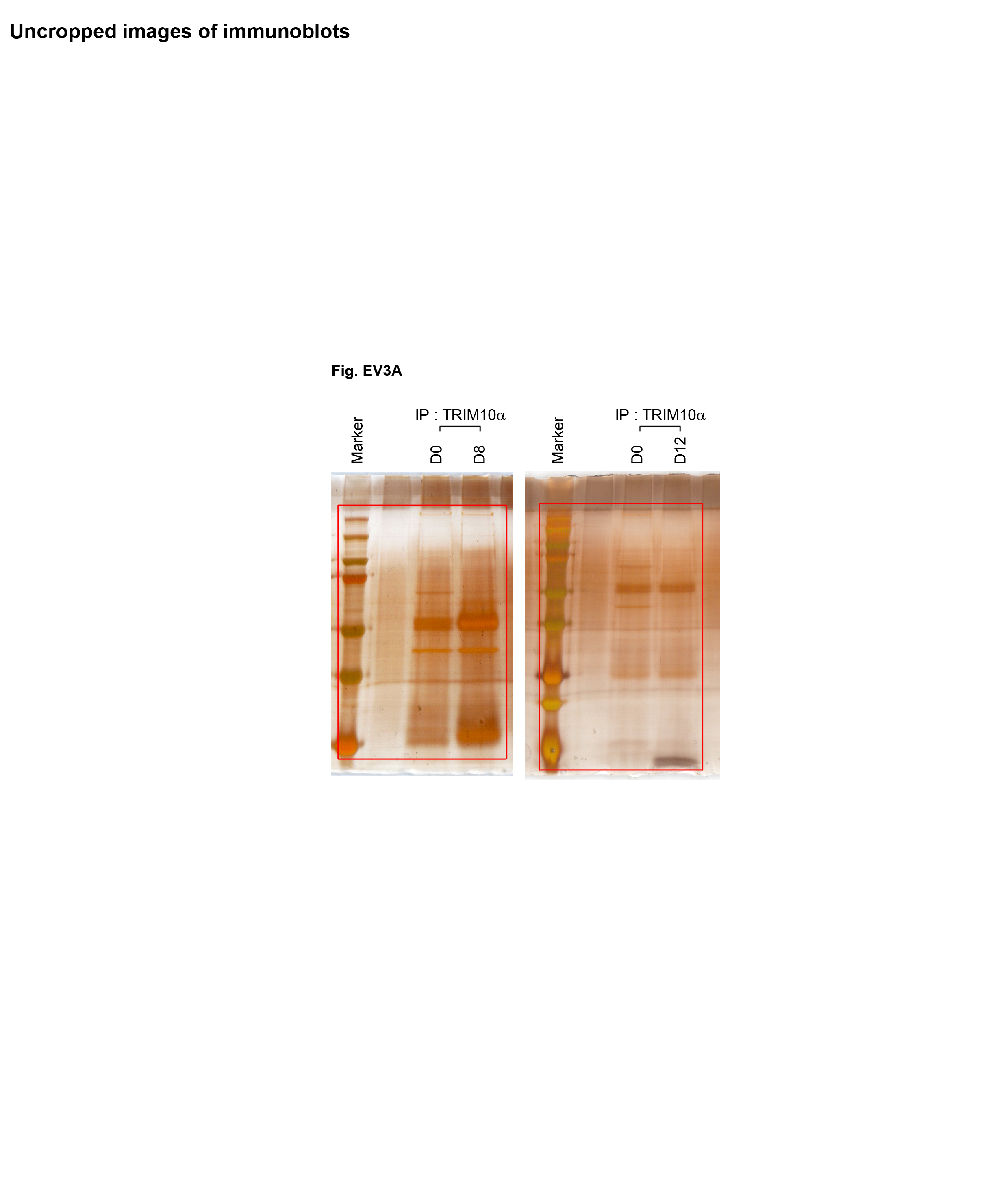

Supplement: Supplementary file 9 — EV Figures Source Data [file 44319_2025_616_MOESM9_ESM.zip › Figure EV3/EV3A/FigEV3A_Blot_data.tif]

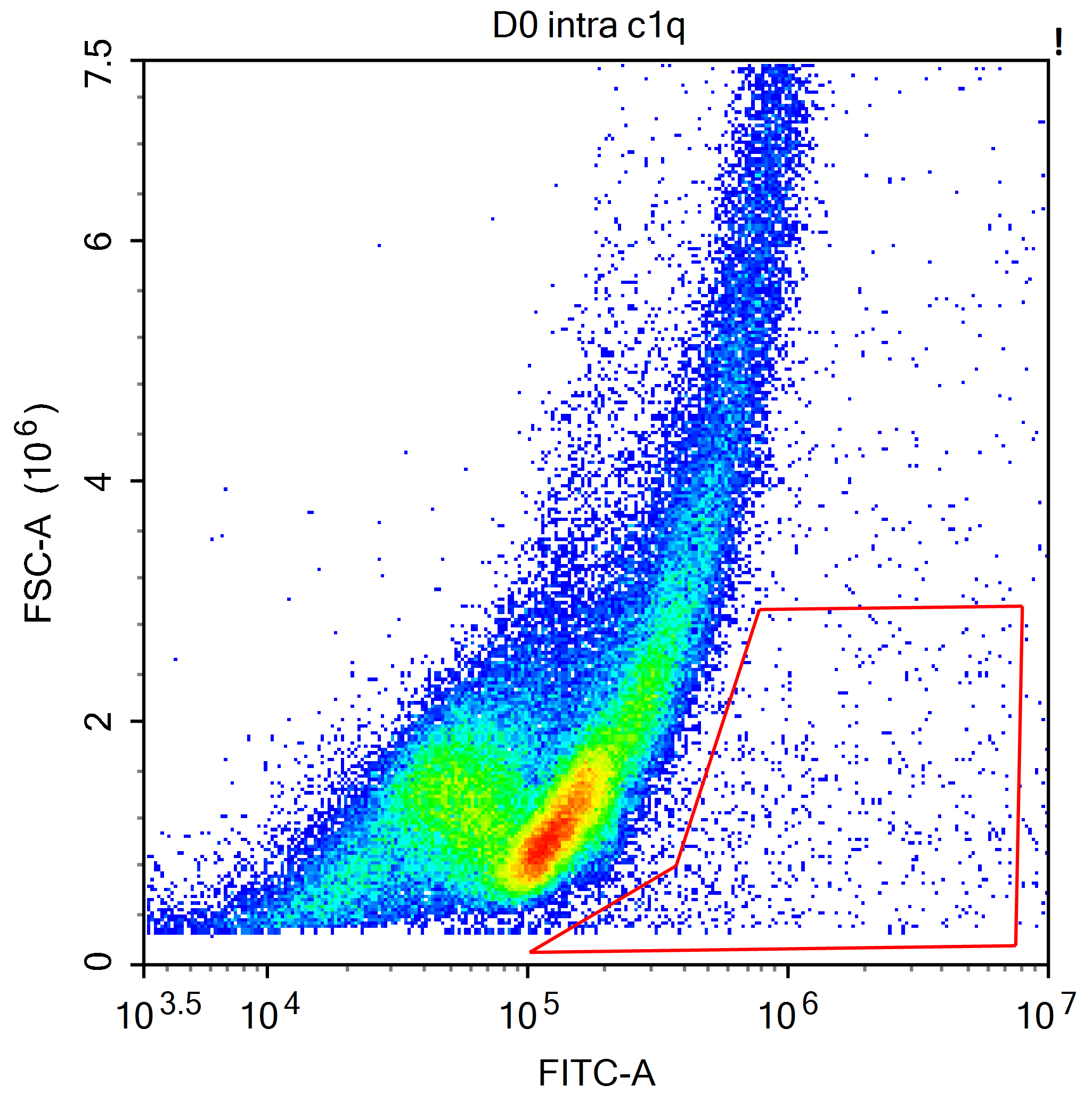

Supplement: Supplementary file 9 — EV Figures Source Data [file 44319_2025_616_MOESM9_ESM.zip › Figure EV3/EV3D/EV3D_D0_Anti-C1q_Intracellular.tiff]

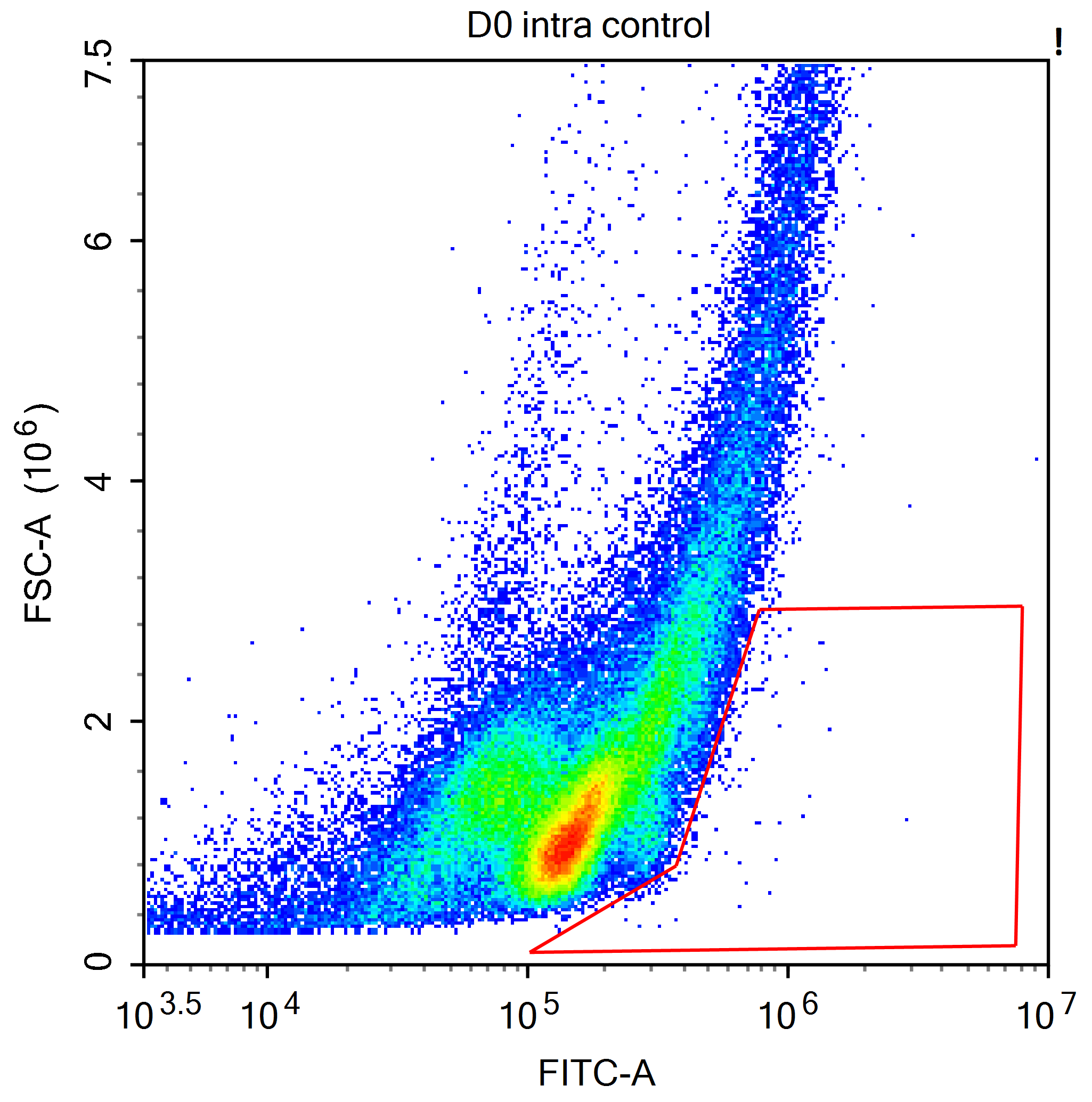

Supplement: Supplementary file 9 — EV Figures Source Data [file 44319_2025_616_MOESM9_ESM.zip › Figure EV3/EV3D/EV3D_D0_Isotype_Control_Intracellular.tiff]

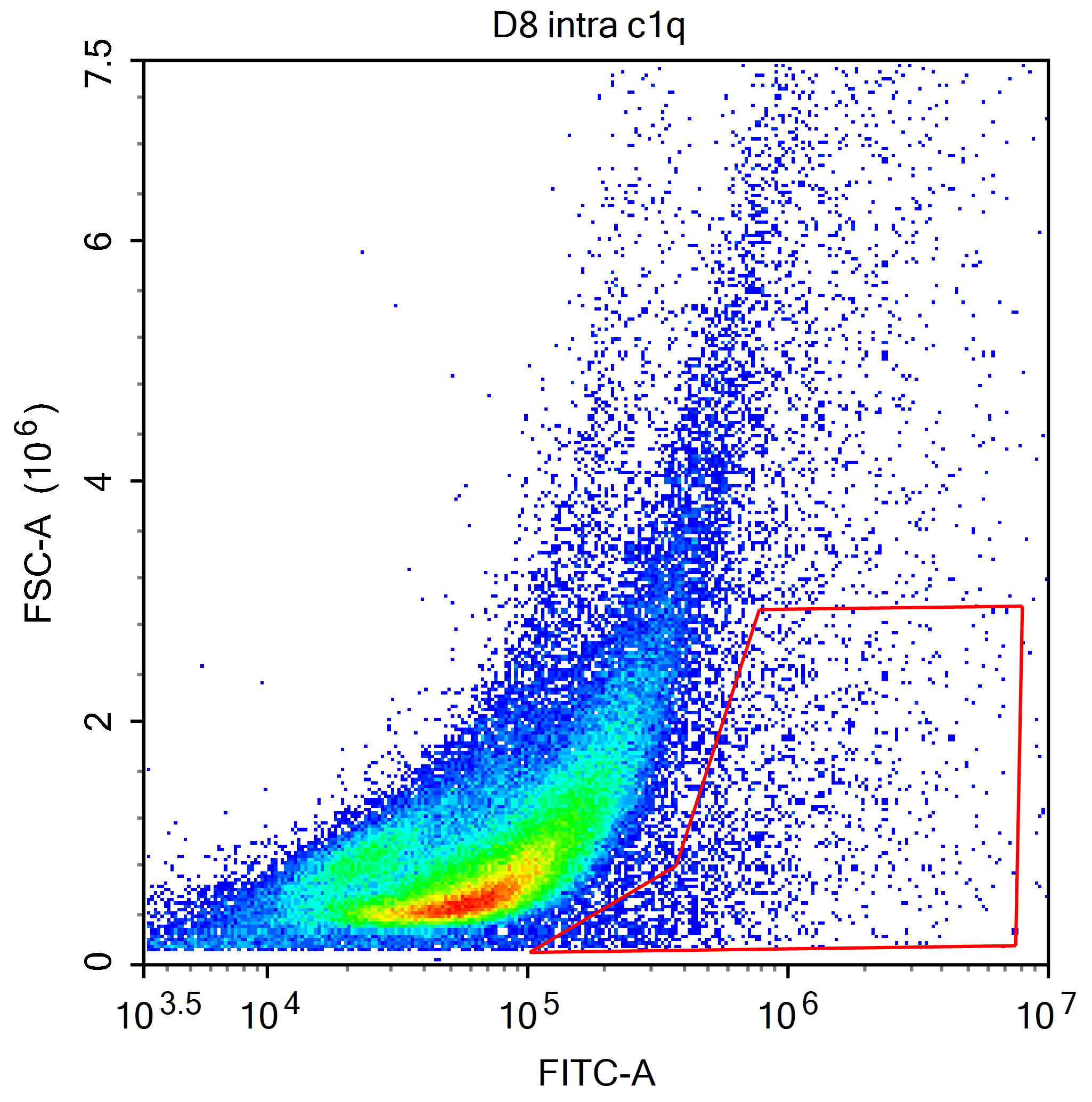

Supplement: Supplementary file 9 — EV Figures Source Data [file 44319_2025_616_MOESM9_ESM.zip › Figure EV3/EV3D/EV3D_D8_Anti-C1q_Intracellular.tiff]

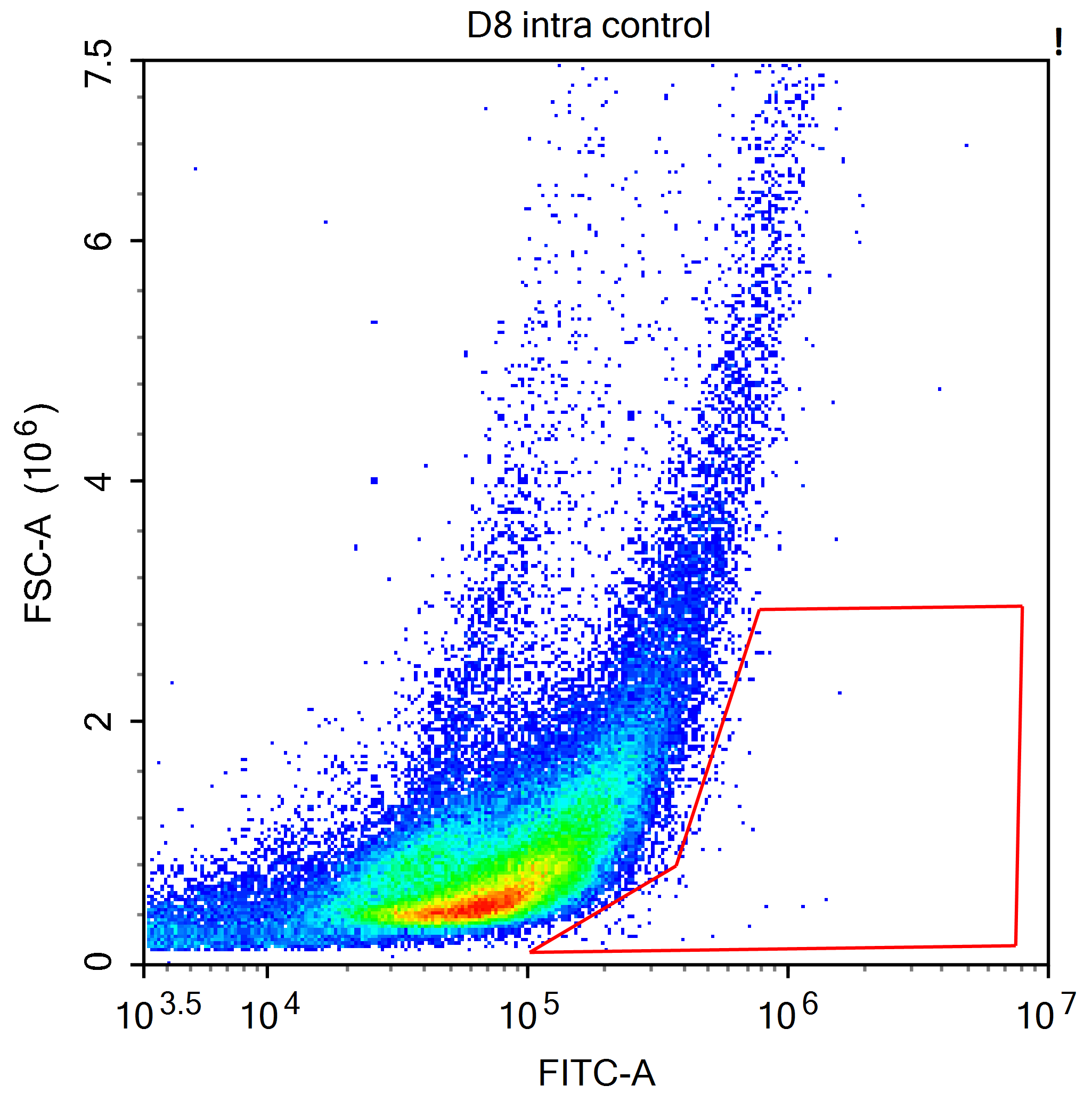

Supplement: Supplementary file 9 — EV Figures Source Data [file 44319_2025_616_MOESM9_ESM.zip › Figure EV3/EV3D/EV3D_D8_Isotype_Control_Intracellular.tiff]

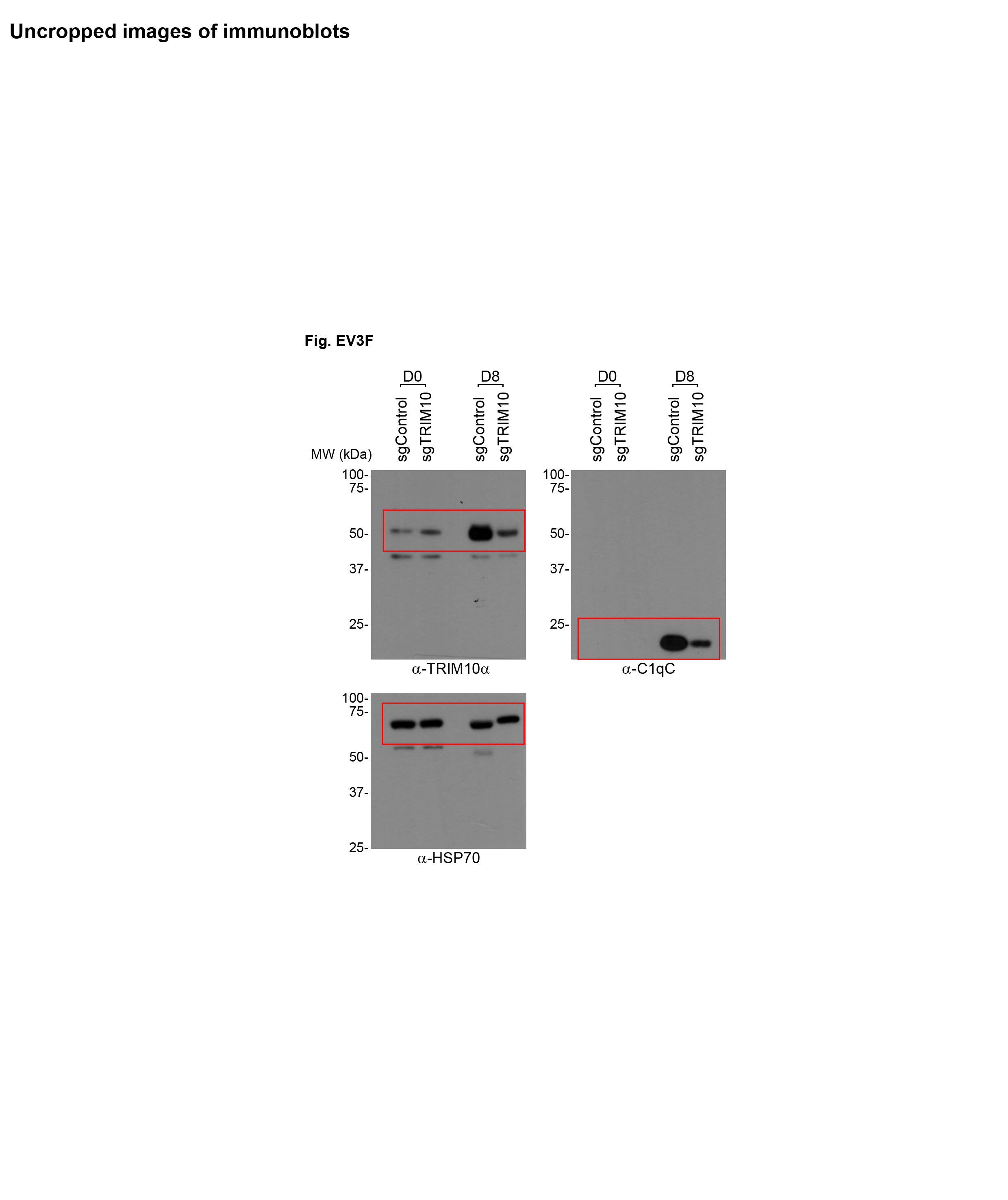

Supplement: Supplementary file 9 — EV Figures Source Data [file 44319_2025_616_MOESM9_ESM.zip › Figure EV3/EV3F/FigEV3F_Blot_data.tif]

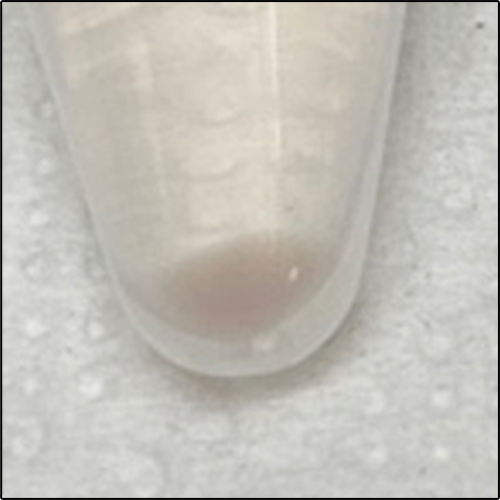

Supplement: Supplementary file 9 — EV Figures Source Data [file 44319_2025_616_MOESM9_ESM.zip › Figure EV3/EV3H/FigEV3H_D0_sgControl.tif]

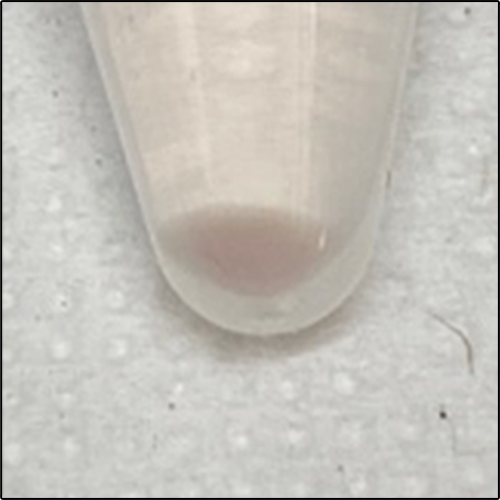

Supplement: Supplementary file 9 — EV Figures Source Data [file 44319_2025_616_MOESM9_ESM.zip › Figure EV3/EV3H/FigEV3H_D0_sgTRIM10.tif]

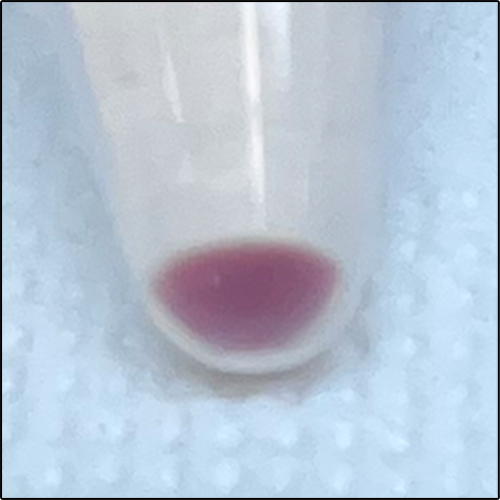

Supplement: Supplementary file 9 — EV Figures Source Data [file 44319_2025_616_MOESM9_ESM.zip › Figure EV3/EV3H/FigEV3H_D8_sgControl.tif]

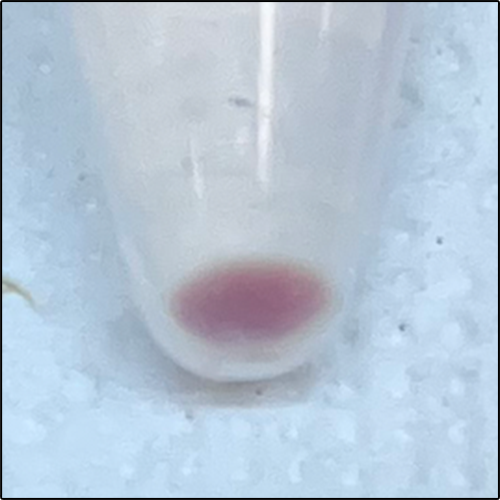

Supplement: Supplementary file 9 — EV Figures Source Data [file 44319_2025_616_MOESM9_ESM.zip › Figure EV3/EV3H/FigEV3H_D8_sgTRIM10.tif]

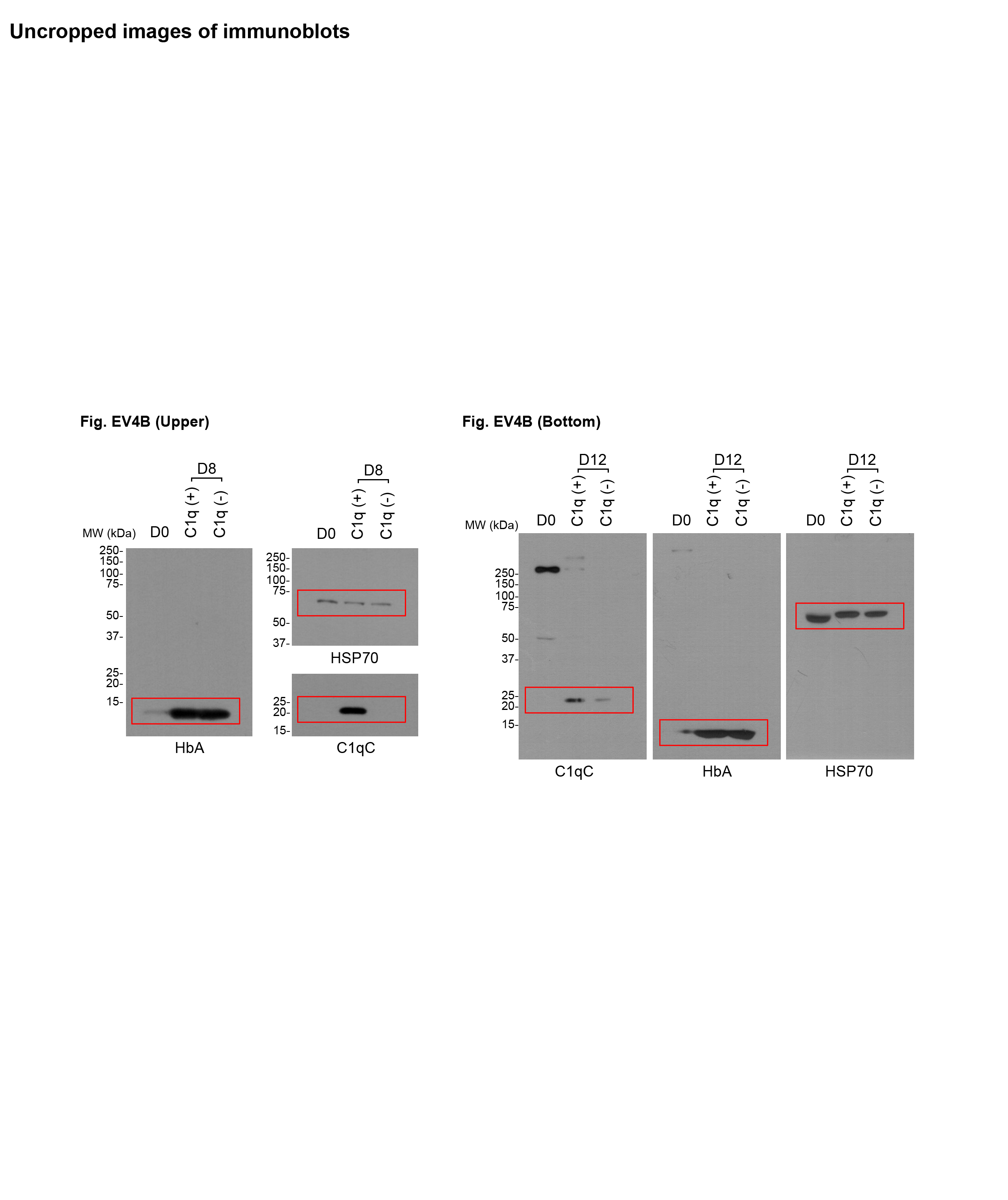

Supplement: Supplementary file 9 — EV Figures Source Data [file 44319_2025_616_MOESM9_ESM.zip › Figure EV4/EV4B/FigEV4B_Blot_data.tif]

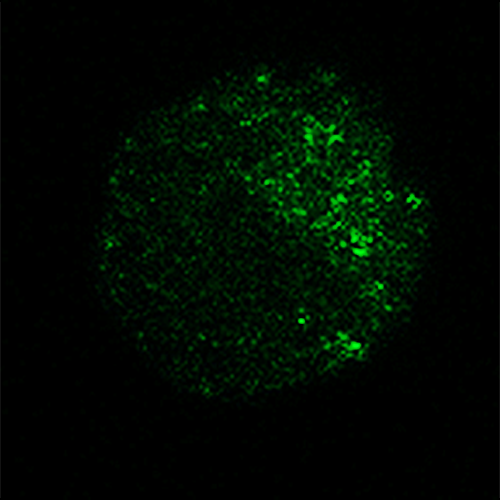

Supplement: Supplementary file 9 — EV Figures Source Data [file 44319_2025_616_MOESM9_ESM.zip › Figure EV4/EV4C/FigEV4C_D0_EPOR.tif]

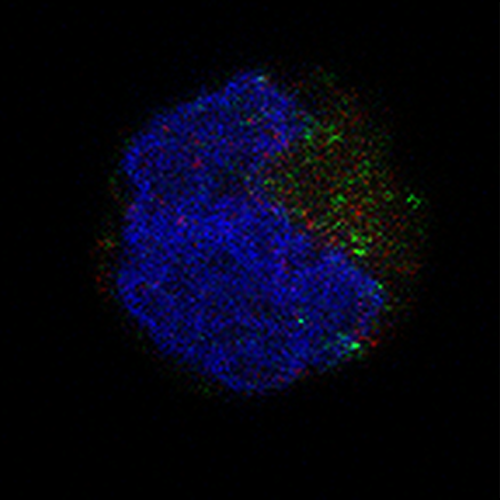

Supplement: Supplementary file 9 — EV Figures Source Data [file 44319_2025_616_MOESM9_ESM.zip › Figure EV4/EV4C/FigEV4C_D0_Merged.tif]

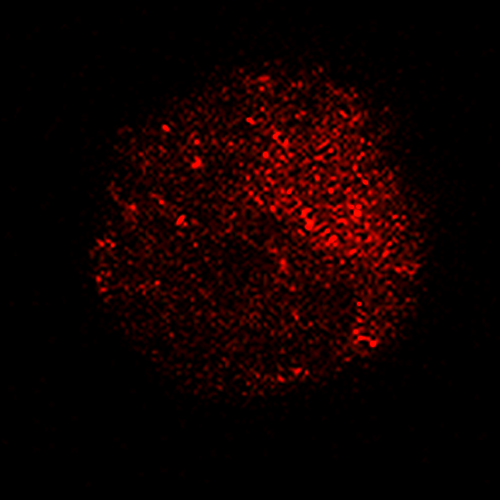

Supplement: Supplementary file 9 — EV Figures Source Data [file 44319_2025_616_MOESM9_ESM.zip › Figure EV4/EV4C/FigEV4C_D0_TRIM10alpha.tif]

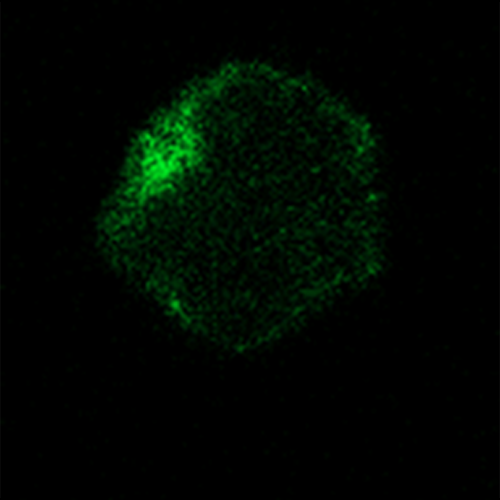

Supplement: Supplementary file 9 — EV Figures Source Data [file 44319_2025_616_MOESM9_ESM.zip › Figure EV4/EV4C/FigEV4C_D4_EPOR.tif]

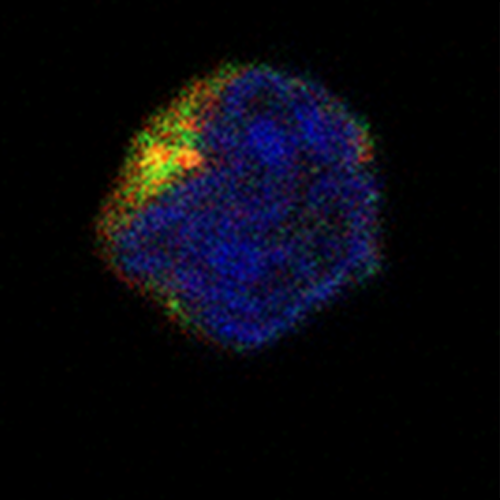

Supplement: Supplementary file 9 — EV Figures Source Data [file 44319_2025_616_MOESM9_ESM.zip › Figure EV4/EV4C/FigEV4C_D4_Merged.tif]

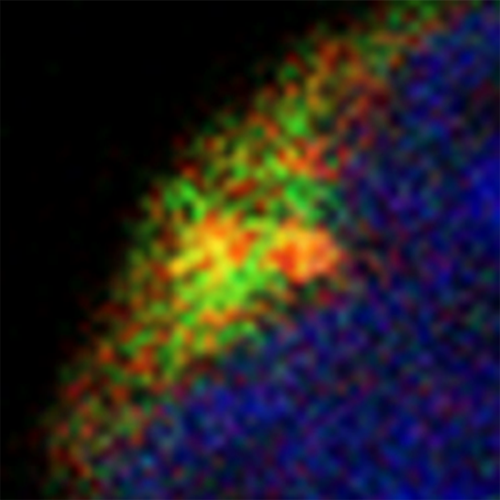

Supplement: Supplementary file 9 — EV Figures Source Data [file 44319_2025_616_MOESM9_ESM.zip › Figure EV4/EV4C/FigEV4C_D4_Merged_enlarged.tif]

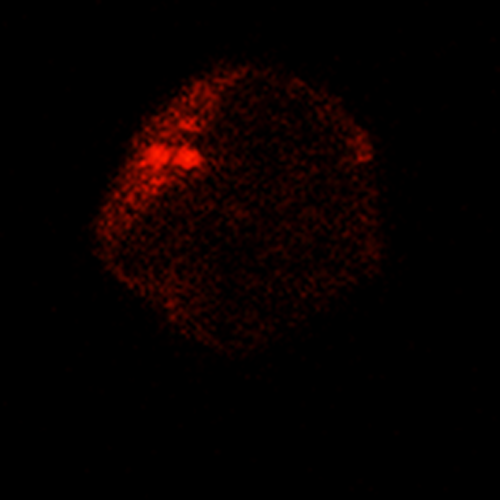

Supplement: Supplementary file 9 — EV Figures Source Data [file 44319_2025_616_MOESM9_ESM.zip › Figure EV4/EV4C/FigEV4C_D4_TRIM10alpha.tif]

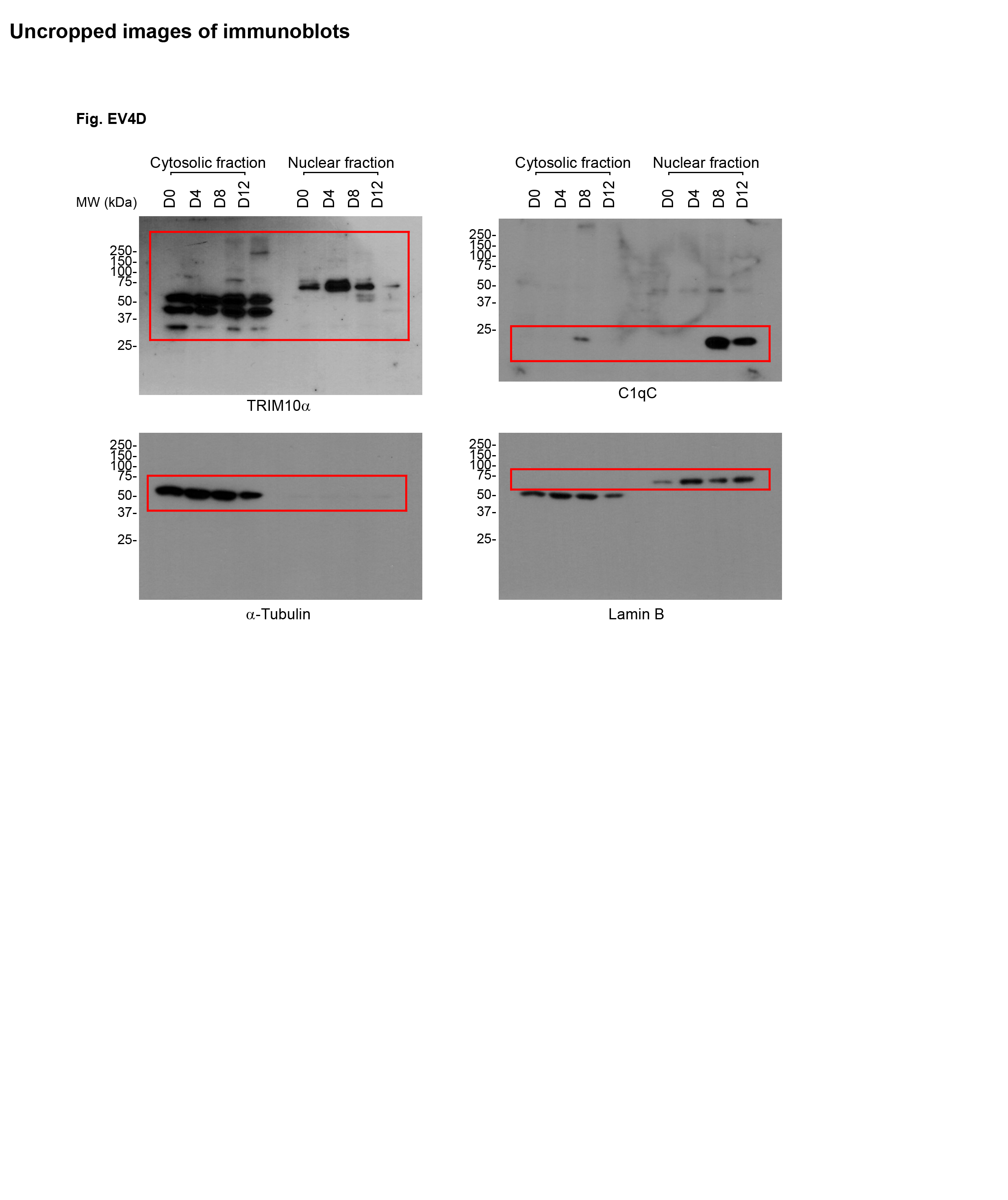

Supplement: Supplementary file 9 — EV Figures Source Data [file 44319_2025_616_MOESM9_ESM.zip › Figure EV4/EV4D/FigEV4D_Blot_data.tif]

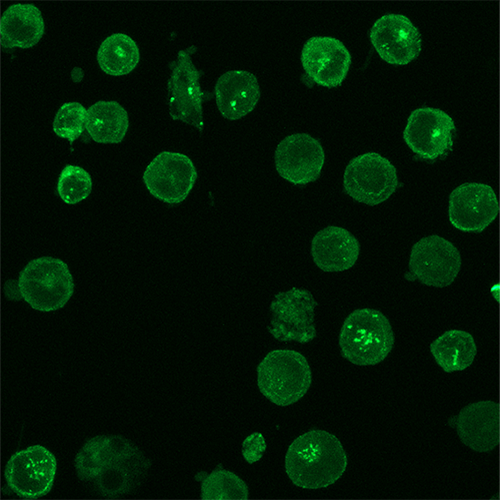

Supplement: Supplementary file 9 — EV Figures Source Data [file 44319_2025_616_MOESM9_ESM.zip › Figure EV5/EV5A/FigEV5A_GFPTRIM10alpha.tif]

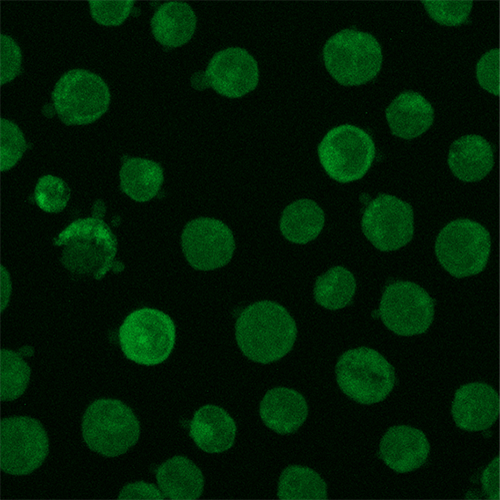

Supplement: Supplementary file 9 — EV Figures Source Data [file 44319_2025_616_MOESM9_ESM.zip › Figure EV5/EV5A/FigEV5A_GFPTRIM10beta.tif]

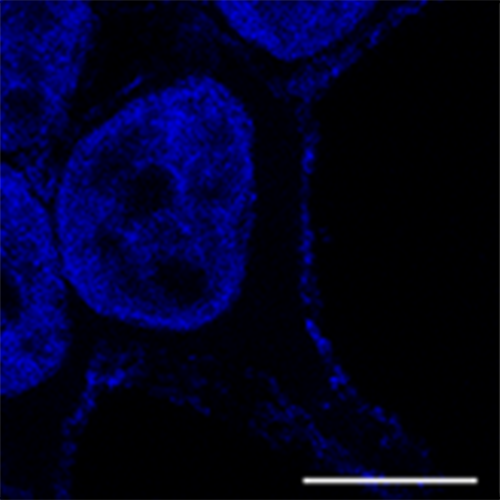

Supplement: Supplementary file 9 — EV Figures Source Data [file 44319_2025_616_MOESM9_ESM.zip › Figure EV5/EV5B/FigEV5B_GFPTRIM10beta_plus1_DAPI.tif]

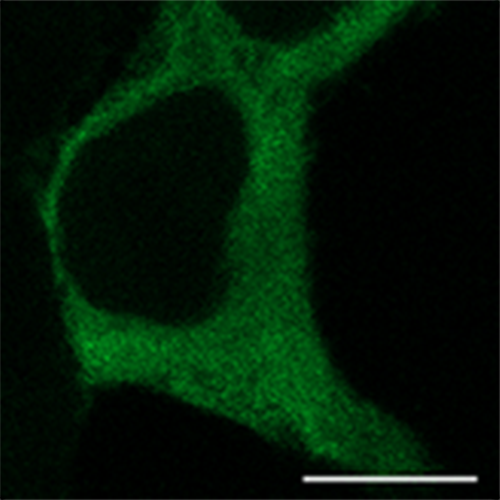

Supplement: Supplementary file 9 — EV Figures Source Data [file 44319_2025_616_MOESM9_ESM.zip › Figure EV5/EV5B/FigEV5B_GFPTRIM10beta_plus1_GFP.tif]

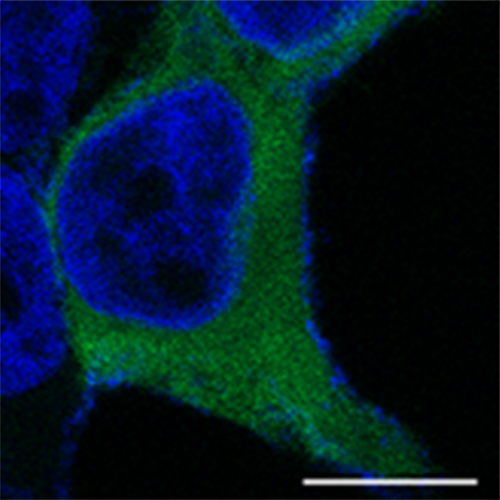

Supplement: Supplementary file 9 — EV Figures Source Data [file 44319_2025_616_MOESM9_ESM.zip › Figure EV5/EV5B/FigEV5B_GFPTRIM10beta_plus1_Merged.tif]

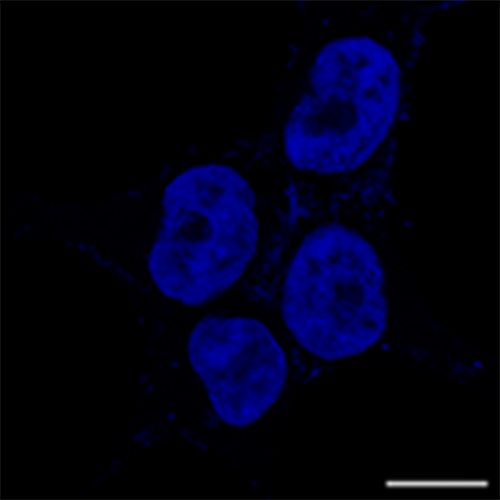

Supplement: Supplementary file 9 — EV Figures Source Data [file 44319_2025_616_MOESM9_ESM.zip › Figure EV5/EV5B/FigEV5B_GFPTRIM10beta_plus2_DAPI.tif]

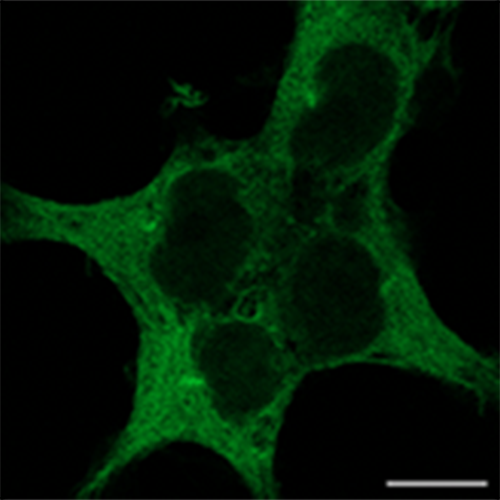

Supplement: Supplementary file 9 — EV Figures Source Data [file 44319_2025_616_MOESM9_ESM.zip › Figure EV5/EV5B/FigEV5B_GFPTRIM10beta_plus2_GFP.tif]

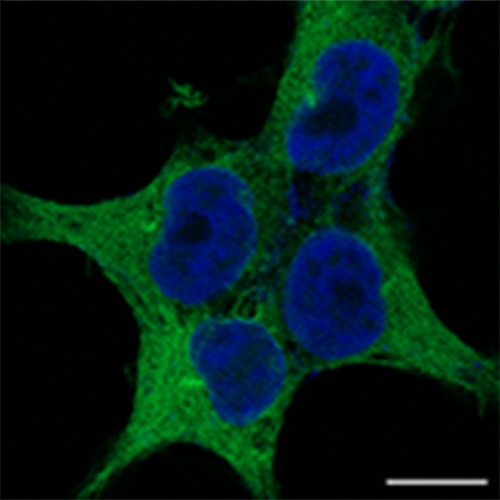

Supplement: Supplementary file 9 — EV Figures Source Data [file 44319_2025_616_MOESM9_ESM.zip › Figure EV5/EV5B/FigEV5B_GFPTRIM10beta_plus2_Merged.tif]

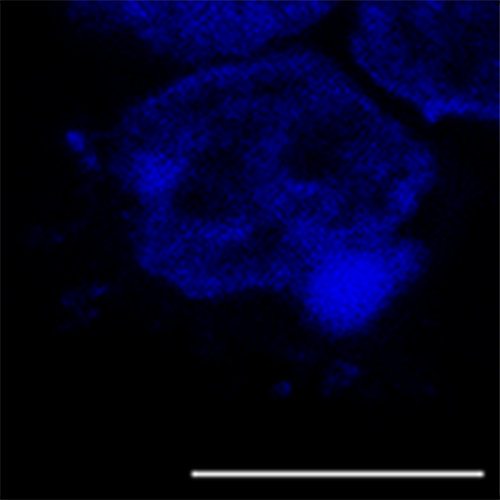

Supplement: Supplementary file 9 — EV Figures Source Data [file 44319_2025_616_MOESM9_ESM.zip › Figure EV5/EV5B/FigEV5B_GFPTRIM10beta_plus3_DAPI.tif]

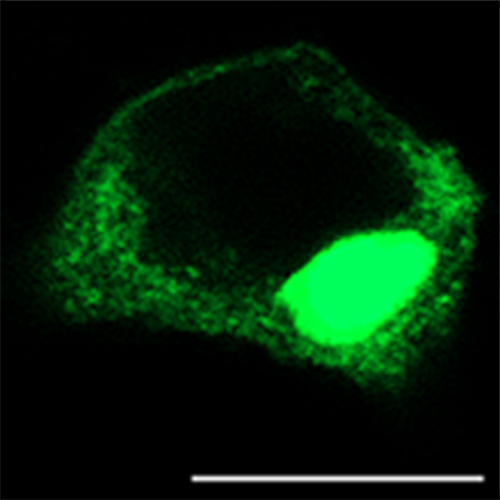

Supplement: Supplementary file 9 — EV Figures Source Data [file 44319_2025_616_MOESM9_ESM.zip › Figure EV5/EV5B/FigEV5B_GFPTRIM10beta_plus3_GFP.tif]

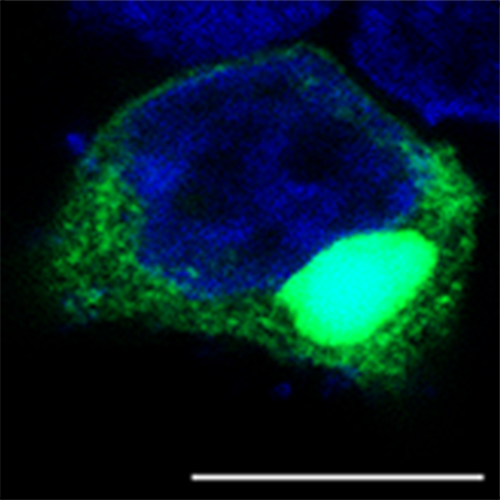

Supplement: Supplementary file 9 — EV Figures Source Data [file 44319_2025_616_MOESM9_ESM.zip › Figure EV5/EV5B/FigEV5B_GFPTRIM10beta_plus3_Merged.tif]

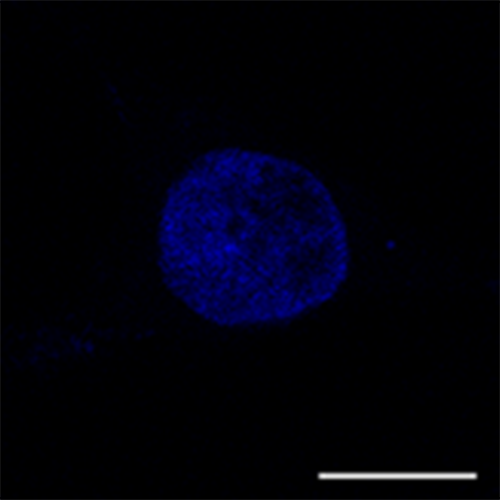

Supplement: Supplementary file 9 — EV Figures Source Data [file 44319_2025_616_MOESM9_ESM.zip › Figure EV5/EV5B/FigEV5B_Mock_DAPI.tif]

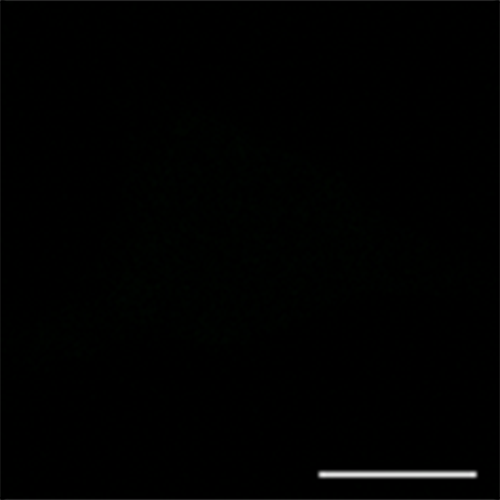

Supplement: Supplementary file 9 — EV Figures Source Data [file 44319_2025_616_MOESM9_ESM.zip › Figure EV5/EV5B/FigEV5B_Mock_GFP.tif]

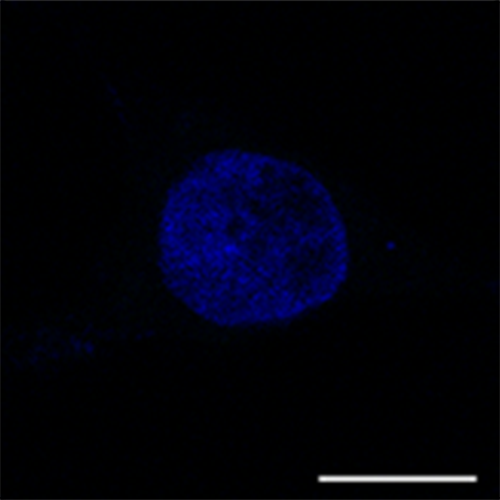

Supplement: Supplementary file 9 — EV Figures Source Data [file 44319_2025_616_MOESM9_ESM.zip › Figure EV5/EV5B/FigEV5B_Mock_Merged.tif]

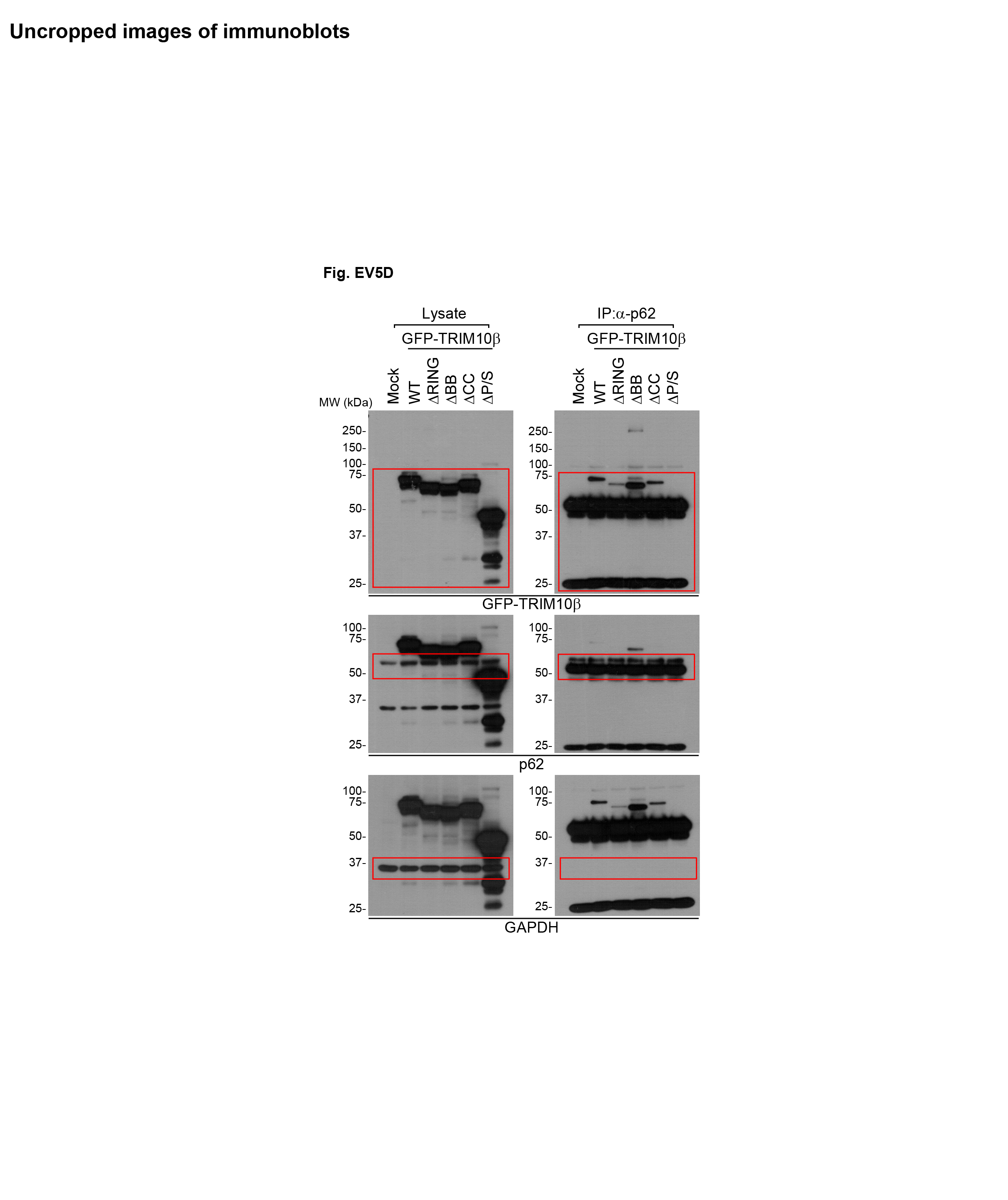

Supplement: Supplementary file 9 — EV Figures Source Data [file 44319_2025_616_MOESM9_ESM.zip › Figure EV5/EV5D/FigEV5D_Blot_data.tif]

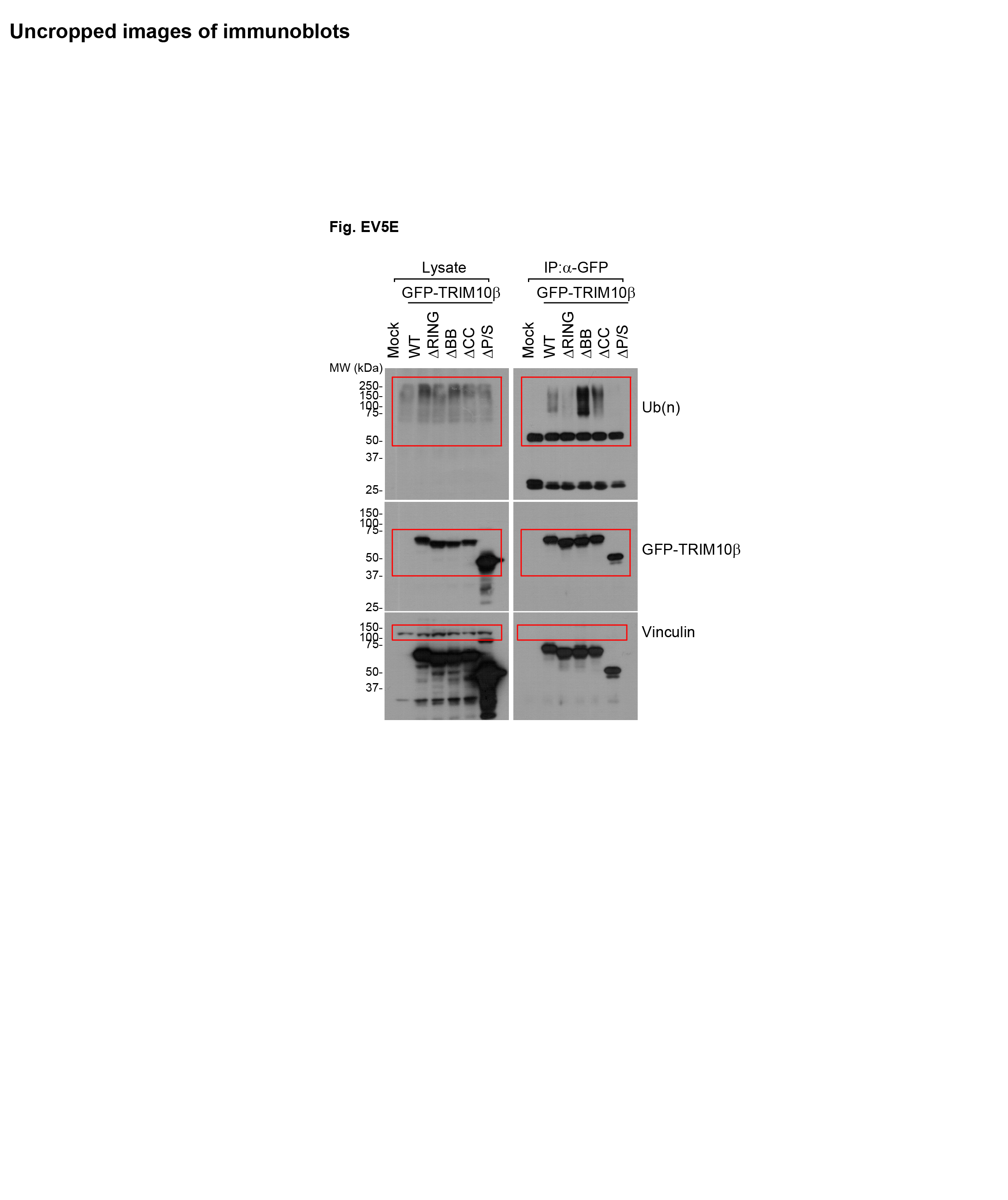

Supplement: Supplementary file 9 — EV Figures Source Data [file 44319_2025_616_MOESM9_ESM.zip › Figure EV5/EV5E/FigEV5E_Blot_data.tif]
