# Supplementary material for: Stage-specific TRIM10 expression regulates erythroid maturation
Source: EMBO Rep. 2025 Oct 30;26(23):5982–6014. doi: 10.1038/s44319-025-00616-0 (PMC12678476; doi:10.1038/s44319-025-00616-0)
Supplement: Supplementary file 10 — Expanded View Figures [file 44319_2025_616_MOESM10_ESM.pdf]

Expanded View Figures

Figure EV1. *TRIM10a* is an erythroid-specific gene with a dynamic expression pattern during erythropoiesis.

(**A**) *TRIM10a* exhibits erythroid lineage-restricted expression across different cell types and tissues (BioGPS) (Wu et al, 2009b; Data ref: Wu et al, 2009a). Heatmap shows absolute mRNA expression, with red and blue representing high and low expression, respectively. (**B**) Violin plots show relative mRNA levels of *mTrim10* in organs during mouse embryonic development. Data were presented as mean ± SD ($n = 3$ biological replicates). P values are determined by unpaired two-tailed t-test. n.s., $P > 0.05$; *$P < 0.05$. (**C**) *mTrim10* expression is increased during mouse erythropoiesis. Violin plots show relative mRNA levels of *mTrim10* in bone marrow (BM)-derived colony-forming units erythroid (CFU-E) cells during ex vivo differentiation at the indicated times. Data are presented as mean ± SD ($n = 3$ biological replicates). P values are determined by unpaired two-tailed t-test. (**D**) *TRIM10a* expression is increased in SCA patients. Comparison of *TRIM10a* expression in public data from whole blood of normal (black) and SCA (red) patients (Raghavachari et al, 2009b; Data ref: Raghavachari et al, 2009a). Data were presented as mean ± SD (n, numbers of patients). P values are determined by unpaired two-tailed t-test. (**E**) Flow cytometry analysis of cell surface marker expression in HUDEP-2 cells cultured in differentiation medium at the indicated times. Cells were dual-stained with anti-CD36-PE or anti-CD49d-PE and anti-CD235a-APC conjugated antibodies. (**F**) HUDEP-2 cells undergo a reduction in size under differentiation conditions. Representative images of HUDEP-2 cells before and after differentiation (D ≥ 10) are shown. Scale bars, 10 µm. (**G**) The expression of *HBA1, HBB*, or *TRIM10a* increases during HUDEP-2 differentiation. qPCR analysis showing relative mRNA expression levels of *HBA1*, *HBB*, or *TRIM10a* in HUDEP-2 cells cultured in differentiation medium for the indicated times. Data were presented as mean ± SD ($n = 3$ biological replicates). P values are determined by unpaired two-tailed t-test. n.s., $P > 0.05$; *$P < 0.05$; **$P < 0.01$; ***$P < 0.001$. Data information: Unless otherwise stated, data shown are mean ± SD of three biological replicates; statistical significance was determined by unpaired two-tailed t-test. (n.s., $P > 0.05$; **$P < 0.01$; ***$P < 0.001$). Source data are available online for this figure.

© The Author(s)

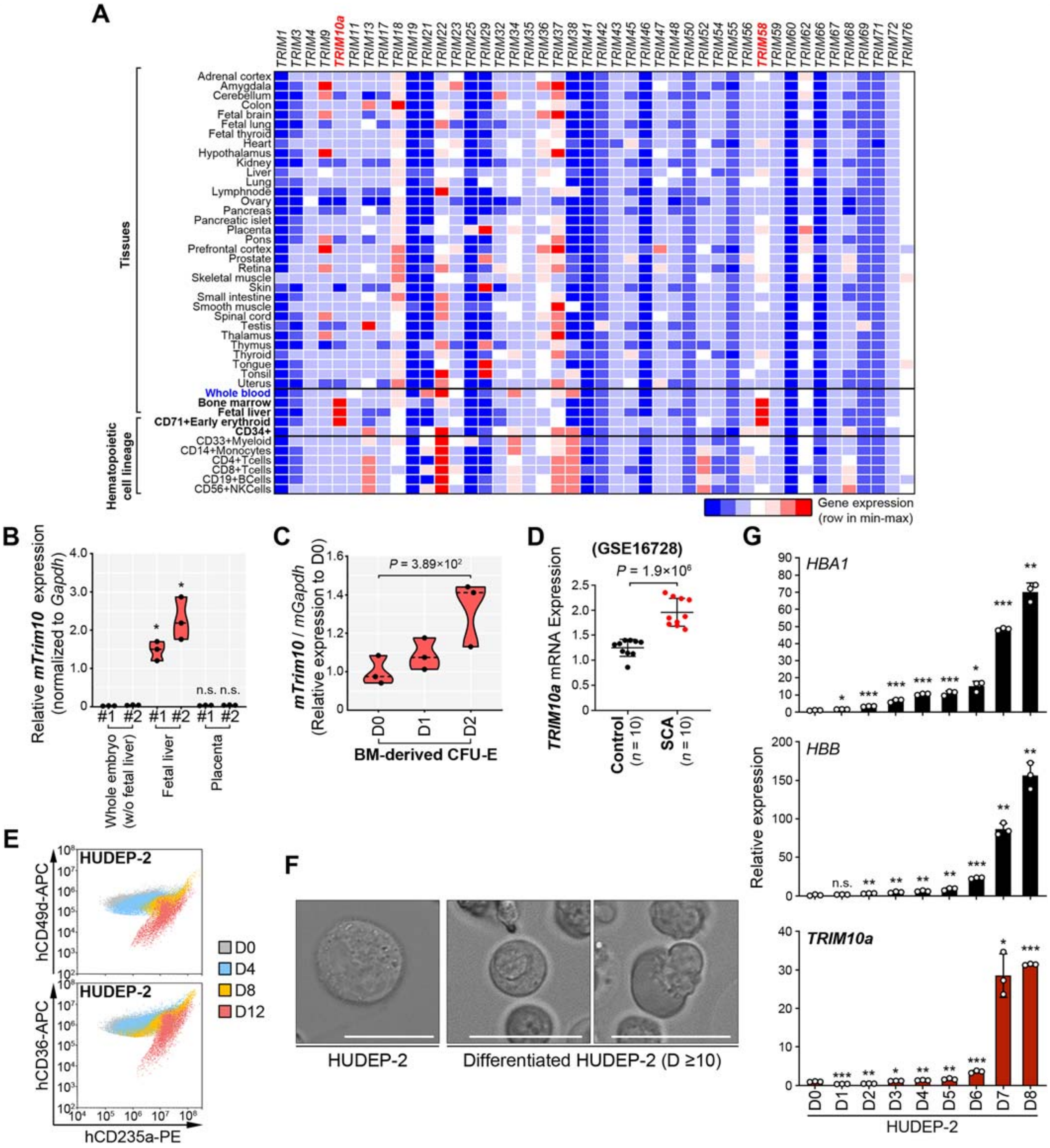
A
Tissues
Hematopoietic cell lineage
Whole blood
Bone marrow
Fetal liver
CD71+Early erythroid
CD34+
Gene expression (row in min-max)
B
Relative mTrim10 expression (normalized to Gapdh)
Whole embryo (w/o fetal liver)
Fetal liver
Placenta
C
mTrim10 / mGapdh (Relative expression to D0)
BM-derived CFU-E
D
(GSE16728)
TRIM10a mRNA Expression
Control (n = 10)
SCA (n = 10)
E
HUDEP-2
hCD49d-APC
hCD36-APC
hCD235a-PE
D0
D4
D8
D12
F
HUDEP-2
Differentiated HUDEP-2 (D ≥10)
G
HBA1
HBB
TRIM10a
Relative expression
HUDEP-2

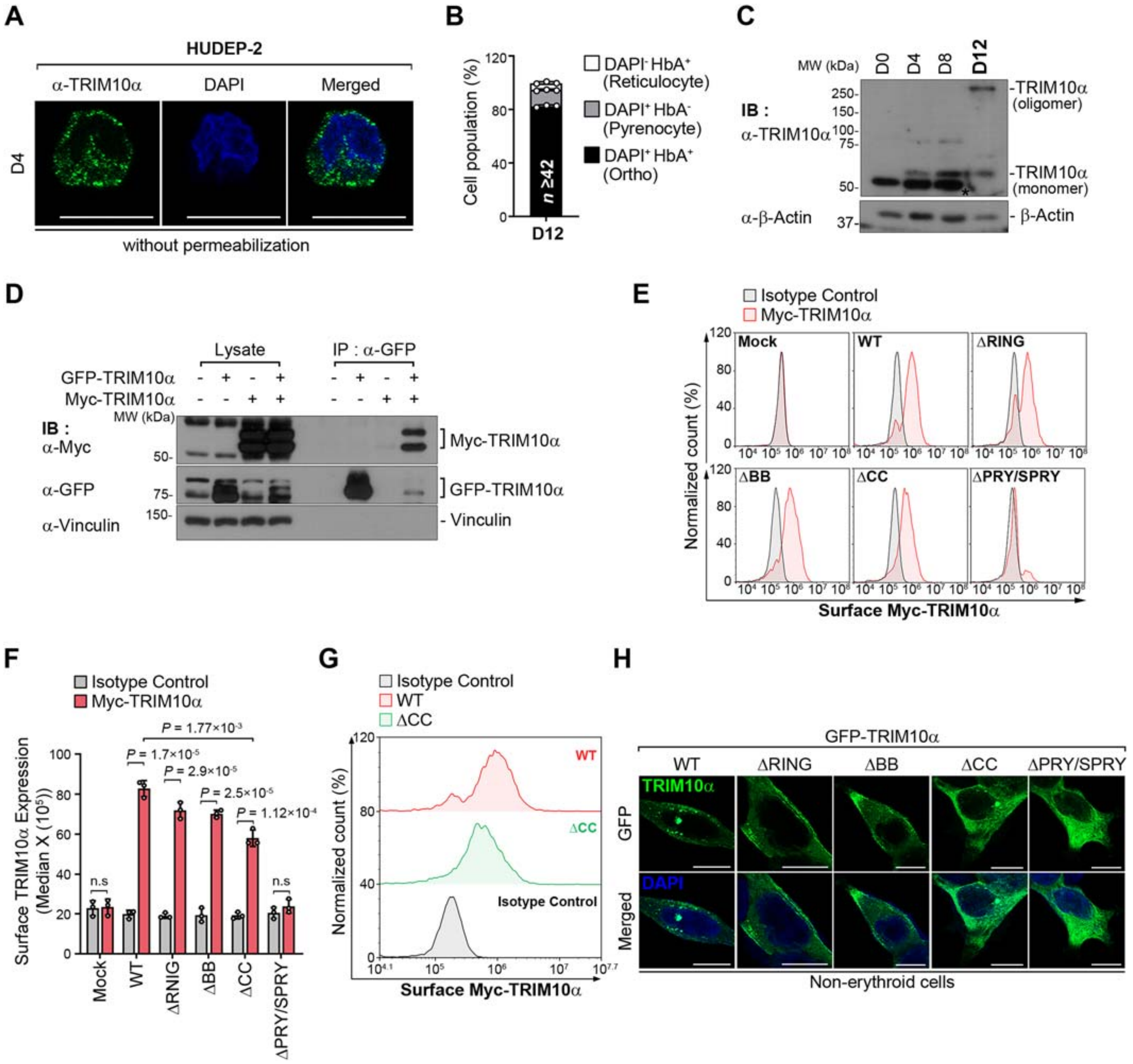

Figure EV2. Dynamics of protein levels and localization of TRIM10α.

(**A**) TRIM10α is localized on the cell surface. Confocal fluorescence images of HUDEP-2 cells cultured in differentiation medium on day 4. Cells were stained with anti-TRIM10α antibody (TRIM10α, green), and nuclei were stained with DAPI (blue) without permeabilization. Images are orthogonal projections of Z-stacks. Scale bars, 10 µm. (**B**) Cell population of HUDEP-2 cells at day 12 of differentiation. Stacked bar graph shows the percentage of HUDEP-2 cell populations on day 12 of differentiation. Pyrenocyte, DAPI$^+$ HbA$^-$ cells (dark gray); Reticulocyte, DAPI$^-$ HbA$^+$ cells (gray); Ortho, DAPI$^+$ HbA$^+$ cells (white). Data were derived from the analysis of multiple cells (n = number of analyzed cells). Data represent mean ± SD of three biological replicates. (**C**) TRIM10α forms oligomers at late stages. Immunoblots showing TRIM10α protein levels in HUDEP-2 cells cultured in differentiation medium for the indicated times. β-Actin was used as the loading control. The asterisk (*) marks the non-specific bands. (**D**) TRIM10α forms a dimer. Co-immunoprecipitation analysis of the interaction between GFP-TRIM10α and Myc-TRIM10α. Vinculin was used as the loading control. (**E**) TRIM10α is localized on the cell surface via its coiled-coil and PRY/SPRY domains. Flow cytometry analysis showing the expression of TRIM10α in HUDEP-2 cells expressing control vector, Myc-WT-TRIM10α, or TRIM10α deletion mutants cultured on expansion medium. (**F**) Bar graphs showing the median fluorescence intensity of TRIM10α expression in (**D**). Data were presented as mean ± SD ($n = 3$ biological replicates). P values are determined by unpaired two-tailed t-test. n.s., $P > 0.05$. (**G**) Coiled-coil domain is required for surface localization of TRIM10α. Flow cytometry analysis showing the expression of TRIM10α in HUDEP-2 cells expressing control vector (gray), Myc-WT-TRIM10α (red), or TRIM10α coiled-coil deletion mutants (green) in (**D**). (**H**) TRIM10α is localized on the cell surface via its coiled-coil and PRY/SPRY domains in non-erythroid cells. Confocal fluorescence images of HEK293T cells expressing control vector, GFP-WT-TRIM10α, or TRIM10α deletion mutants. Nuclei were stained with DAPI (blue). Images are orthogonal projections of Z-stacks. Scale bars, 10 µm. Data information: Unless otherwise stated, data shown are mean ± SD of three biological replicates; statistical significance was determined by unpaired two-tailed t-test. (n.s., $P > 0.05$). Source data are available online for this figure.

 © The Author(s)

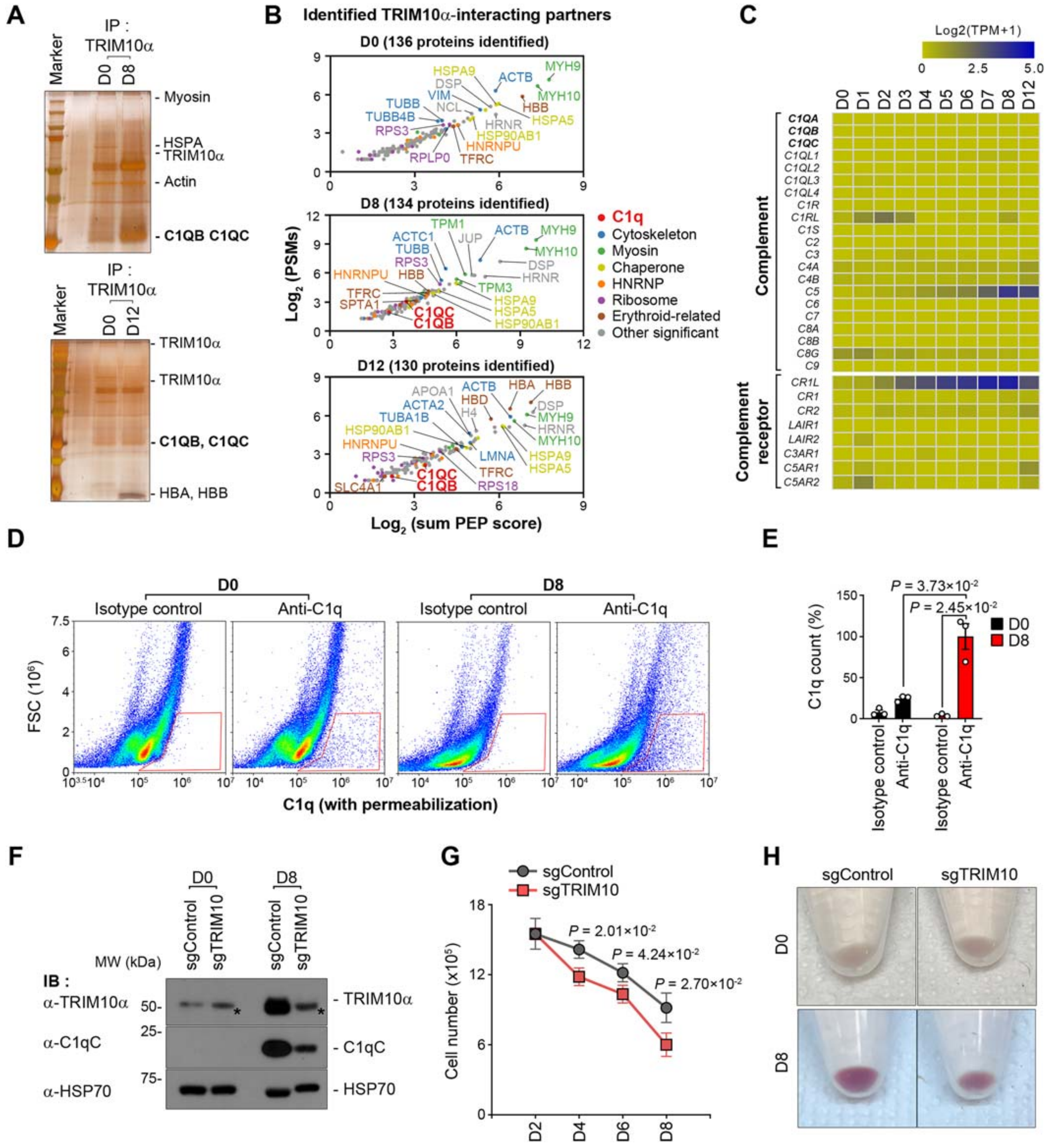
A
IP : TRIM10α
Marker
D0
D8
Myosin
HSPA
TRIM10α
Actin
C1QB C1QC
IP : TRIM10α
Marker
D0
D12
TRIM10α
TRIM10α
C1QB, C1QC
HBA, HBB
B
Identified TRIM10α-interacting partners
D0 (136 proteins identified)
D8 (134 proteins identified)
D12 (130 proteins identified)
Log2 (PSMs)
Log2 (sum PEP score)
C1q
Cytoskeleton
Myosin
Chaperone
HNRNP
Ribosome
Erythroid-related
Other significant
C
Log2(TPM+1)
Complement
Complement receptor
D
D0
D8
Isotype control
Anti-C1q
FSC (10⁶)
C1q (with permeabilization)
E
P = 3.73×10⁻²
P = 2.45×10⁻²
C1q count (%)
D0
D8
Isotype control
Anti-C1q
F
D0
D8
sgControl
sgTRIM10
MW (kDa)
IB :
α-TRIM10α
α-C1qC
α-HSP70
TRIM10α
C1qC
HSP70
G
sgControl
sgTRIM10
Cell number (x10⁵)
P = 2.01×10⁻²
P = 4.24×10⁻²
P = 2.70×10⁻²
D2
D4
D6
D8
H
sgControl
sgTRIM10
D0
D8

Figure EV3. Mass spectrometry analysis of TRIM10α during erythropoiesis.

(**A**) Silver staining and mass spectrometry analysis of TRIM10α-binding proteins purified from HUDEP-2 cells cultured in differentiation medium on day 8 (Top, D8) or day 12 (Bottom, D12). (**B**) Proteins identified by mass spectrometry of TRIM10α-binding partners. Graphs show TRIM10α-interacting proteins identified by mass spectrometry from HUDEP-2 cells cultured in differentiation medium at the indicated times across two biological replicates. A total of 136 proteins at D0, 134 proteins at D8, and 130 proteins at D12 were identified. (**C**) Heatmap showing the relative expression of complements or complement receptors by RNA-seq in HUDEP-2 cells before and after differentiation. Blue and yellow colors represent high and low fold change, respectively. (**D**) C1q is detected in differentiated cells. Flow cytometry analysis of C1q in HUDEP-2 cells before (D0) and after (D8) differentiation under permeabilization conditions. Plots show the number of intracellular $C1q^+$ cells. (**E**) Graphs showing the percentage of counted $C1q^+$ cell numbers in (**D**). Data were presented as mean ± SEM ($n = 3$ biological replicates). *P* values are determined by unpaired two-tailed *t*-test. (**F**) Reduction of TRIM10α expression leads to decreased C1qC. Immunoblots showing C1qC and TRIM10α protein levels in HUDEP-2 cells expressing either a control vector or sgTRIM10 cultured in differentiation medium for the indicated times. HSP70 was used as the loading control. The asterisk (*) marks the non-specific bands. (**G**) Cell number decreases during differentiation under TRIM10-depleted conditions. Graph shows the number of HUDEP-2 cells expressing either a control vector (dark gray) or sgTRIM10 (red) cultured in differentiation medium for the indicated times. Data were presented as mean ± SD ($n = 3$ biological replicates). *P* values are determined by unpaired two-tailed *t*-test. (**H**) Cell redness decreases during differentiation under TRIM10-depleted conditions. Images show the cell pellet redness of HUDEP-2 cells expressing either a control vector or sgTRIM10 cultured in differentiation medium for the indicated times. Data information: Unless otherwise stated, data shown are mean ± SD of three biological replicates; statistical significance was determined by unpaired two-tailed *t*-test. Source data are available online for this figure.

 © The Author(s)

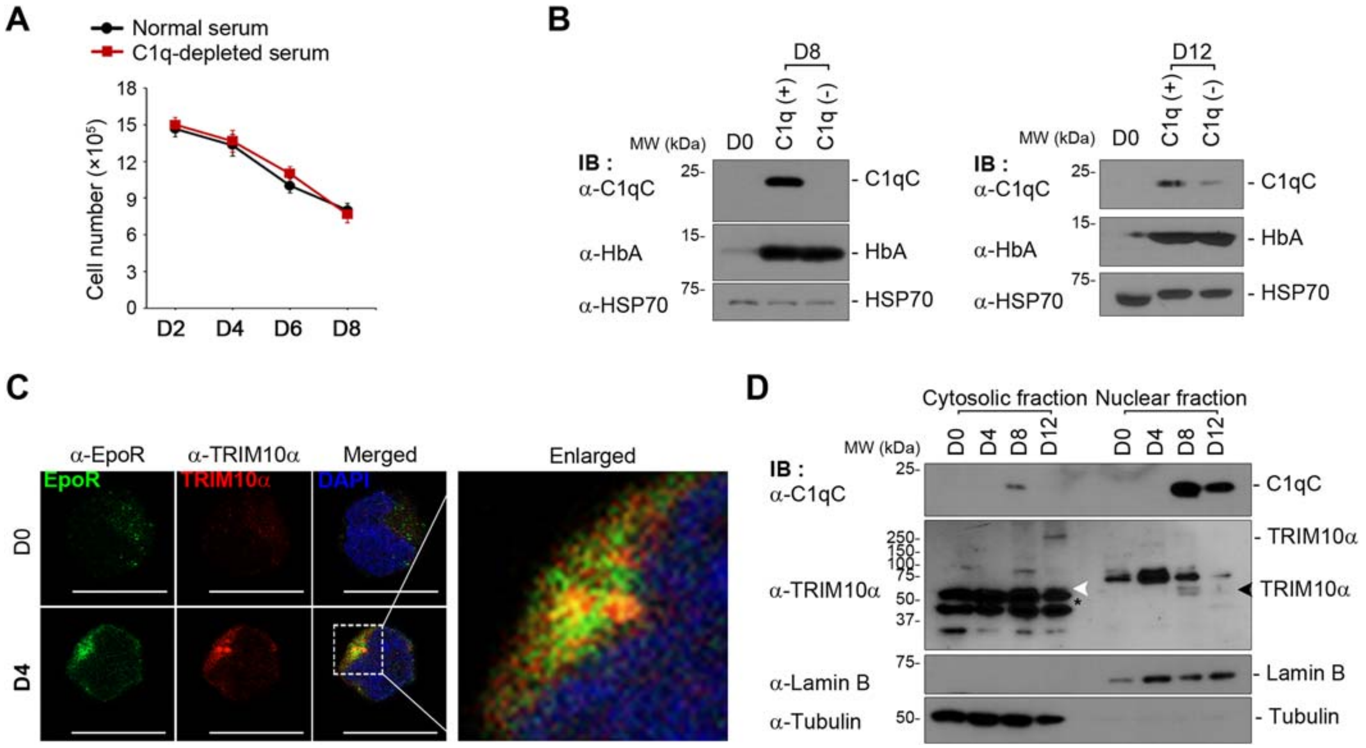

Figure EV4. C1q and TRIM10α are present in both the cytosolic and nuclear fractions.

(**A**) C1q depletion does not affect cell viability or hemoglobin synthesis. Cell numbers under normal or C1q-depleted conditions. Graph shows the number of HUDEP-2 cells counted after differentiation with normal serum (black) or C1q-depleted serum (red) at the indicated times. Data are presented as mean ± SD ($n = 3$ biological replicates). (**B**) Hemoglobin synthesis under normal or C1q-depleted conditions. Immunoblot analysis of C1qC and HbA from whole cell lysates of HUDEP-2 cells before (D0) or after differentiation (D8 or D12) with normal serum or C1q-depleted serum. HSP70 was used as a loading control. (**C**) EpoR colocalizes with TRIM10α. Confocal fluorescence images of HUDEP-2 cells before (D0) and after (D4) differentiation. Cells were stained with anti-EpoR antibody (EpoR, green), anti-TRIM10α antibody (TRIM10α, red), and nuclei were stained with DAPI (blue). Enlarged views of the regions are indicated by white dashed squares. Images are orthogonal projections of Z-stacks. Scale bars, 10 µm. (**D**) Immunoblots showing C1qC and TRIM10α in the cytosolic and nuclear fraction of HUDEP-2 cells cultured in differentiation medium at the indicated times. α-Tubulin was used as the loading control. Arrowheads represent the monomeric TRIM10α. The asterisk (*) marks the non-specific bands. Data information: Data shown are from three biological replicates. Source data are available online for this figure.

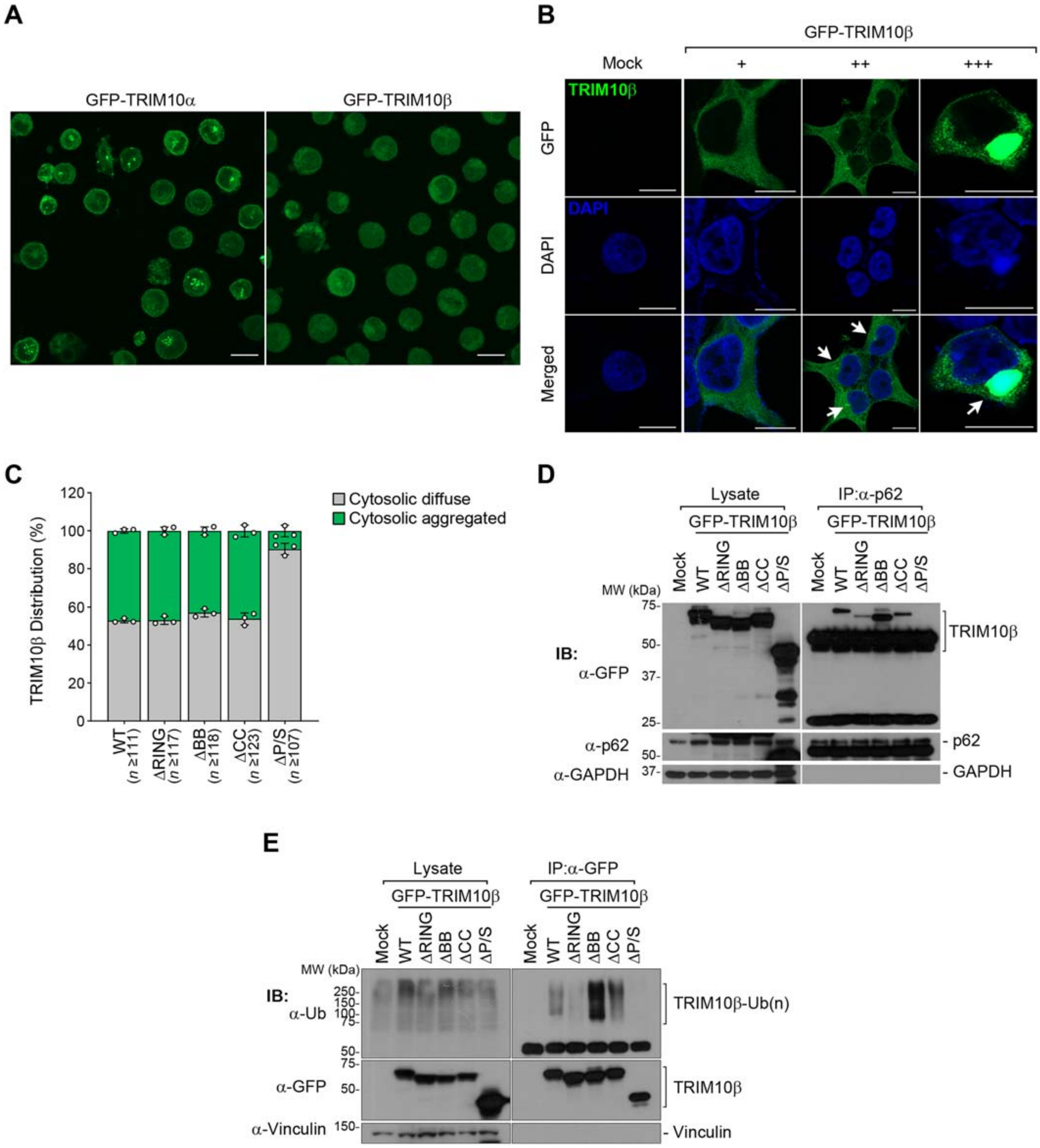
A
GFP-TRIM10α
GFP-TRIM10β
B
GFP-TRIM10β
Mock
+
++
+++
TRIM10β
GFP
DAPI
DAPI
Merged
C
Cytosolic diffuse
Cytosolic aggregated
TRIM10β Distribution (%)
120
100
80
60
40
20
0
WT (n ≥111)
ΔRING (n ≥117)
ΔBB (n ≥118)
ΔCC (n ≥123)
ΔP/S (n ≥107)
D
Lysate
IP:α-p62
GFP-TRIM10β
GFP-TRIM10β
Mock
WT
ΔRING
ΔBB
ΔCC
ΔP/S
MW (kDa)
IB:
α-GFP
α-p62
α-GAPDH
75-
50-
37-
25-
TRIM10β
- p62
- GAPDH
E
Lysate
IP:α-GFP
GFP-TRIM10β
GFP-TRIM10β
Mock
WT
ΔRING
ΔBB
ΔCC
ΔP/S
MW (kDa)
IB:
α-Ub
250-
150-
100-
75-
50-
TRIM10β-Ub(n)
α-GFP
TRIM10β
α-Vinculin
150-
- Vinculin

 © The Author(s)

Figure EV5. TRIM10β forms proteotoxic aggregates.

(**A**) Wide-field fluorescent images of HUDEP-2 cells expressing GFP-TRIM10α or GFP-TRIM10β, showing multiple cells within the same field of view. Nuclei were stained with DAPI (blue). Scale bars, 10 μm. (**B**) The size of aggregates increases with higher levels of ectopic TRIM10β expression. Confocal fluorescence images of HEK293T cells expressing control vector or GFP-TRIM10β. The number of plus signs indicates an increase in ectopic GFP-TRIM10β expression levels. Nuclei were stained with DAPI (blue). White arrows represent the TRIM10β aggregates. Images are orthogonal projections of Z-stacks. Scale bars, 10 μm. (**C**) Distribution of TRIM10β in confocal microscopy images from Fig. 7I. Cells were classified as cytosolic diffuse (gray) or cytosolic aggregated (green). Data were derived from the analysis of multiple cells ($n =$ number of analyzed cells). Data represent mean ± SD of three biological replicates. (**D**) TRIM10β PRY/SPRY domain is required for binding to p62. Immunoblots showing the interaction between p62 and TRIM10β in HEK293T cells expressing control vector, GFP-WT-TRIM10β, or GFP-TRIM10β deletion mutants. GAPDH was used as a loading control. (**E**) TRIM10β self-ubiquitinates through its RING domain. Immunoblots showing TRIM10β ubiquitination in HEK293T cells expressing control vector, GFP-WT-TRIM10β, or GFP-TRIM10β deletion mutants. Vinculin was used as a loading control. Data information: Data shown are mean ± SD of three biological replicates. Source data are available online for this figure.

© The Author(s)
